# Supplementary material for: Commitment to honesty oaths decreases dishonesty, but commitment to another individual does not affect dishonesty
Source: Commun Psychol. 2023 Oct 24;1:27. doi: 10.1038/s44271-023-00028-7 (PMC11332245; doi:10.1038/s44271-023-00028-7)
Supplement: Supplementary file 1 — Supplemental Material [file 44271_2023_28_MOESM1_ESM.pdf]

Commitment to Honesty Oaths Decreases Dishonesty, but Commitment to Another Individual Does Not Affect Dishonesty

Janis H. Zickfeld Karolina Aleksandra Ścigała
Aarhus University

Alexa Weiss
Bielefeld University

John Michael
University of Milan

Panagiotis Mitkidis
Aarhus University

Author Note
Janis H. Zickfeld, Department of Management, Aarhus University Fuglesangsallé 4, 8210 Aarhus, jhzickfeld@gmail.com; Karolina Aleksandra Ścigała, Department of Psychology, Aarhus University; Alexa Weiss, Department of Psychology, Bielefeld University; John Michael, Department of Philosophy, University of Milan; Panagiotis Mitkidis, Department of Management, Aarhus University.

Table of Contents

1. Supplementary Methods 5

1.1 Study 1 5

1.2 Study 2 10

1.3 Study 3 14

1.4 Study 4 & 5 17

1.5 Study 6 23

1.6 Study 7 25

1.7 Live Interaction Details. 27

2. Supplementary Note 1 - Sample Size Determination 28

2.1 Supplementary Note 2 - Sensitivity Analyses. 29

3. Supplementary Note 3 - Designs 29

4. Supplementary Note 4 - Procedures 29

5. Supplementary Note 5 - Additional Measures 30

6. Supplementary Note 6 – Assumptions for main Analyses 31

7. Supplementary Note 7 - Determination of SESOI 33

8. Manipulation Check – Felt Commitment 34

9. Equivalence Tests. 35

10. Supplementary Note 8 – Registered Analyses Study 1 36

10. Sensitivity Analysis Study 1 & 2 39

10.1 Study 1. 39

10.2 Study 2. 41

10.3 Study 7. 42

11. Moderation Analysis 44

11.1 Study 1. 44

11.2 Study 2. 47

11.3 Study 3. 51

11.4 Study 4. 55

11.5 Study 5 59

11.6 Study 6. 64

11.7 Study 7. 66

12. Honesty-Humility 67

13. Trial Effects 70

13.1 Study 1. 72

13.2 Study 2. 75

13.3 Study 3. 76

13.4 Study 4. 77

13.5 Study 5. 78

13.6 Study 7. 80

14. Responsibility Models 82

14.1 Study 1. 83

14.2 Study 2. 84

14.3 Study 3. 84

14.4 Study 4. 85

14.5 Study 5. 86

14.6 Study 6. 86

15. Socioeconomic Status 87

15.1 Study 1. 87

15.2 Study 2. 88

15.3 Study 3. 90

15.4 Study 4. 91

15.5 Study 5 92

15.6 Study 6. 93

15.7 Study 7. 94

16. Gender 96

16.1 Study 1. 97

16.2 Study 2. 98

16.3 Study 3. 99

16.4 Study 4. 100

16.5 Study 5. 101

16.6. Study 6. 103

16.5 Study 7. 104

17. Age 106

17.2 Study 2. 106

17.3 Study 3. 107

17.4 Study 4. 108

17.5 Study 5. 109

17.6 Study 6. 111

17.6 Study 7. 112

18. Commitment & Dishonesty Rating 114

19. Comparison Across Treatments 118

20. Country Differences 119

21. Additional Analyses on Dyads in Study 7 120

22. Dishonesty Analyses 122

Supplementary References 124

1. Supplementary Methods

1.1 Study 1

Method

Participants

Sample Size Justification. Based on a previous meta-analysis[1] effects of minimal group paradigm manipulations on dishonesty (H1) were g = -.24 [-.36, -.12], while effects of oath paradigms on dishonesty (H2) were g = .26 [.15, .37]. Because power calculations for ordinal logistic regressions are complex, we focused on the smallest effect size (H1) and calculated the power for the two-way comparison using the *posamsize* function in the *hmisc* package[2]. We used the frequencies of the distribution of payoffs (die rolls from 1 to 6) based on data by[3]. Focusing on an odds ratio of .647 (transformed from a g = -.24), an alpha level of .05, and a power of .80, we observed a full sample size of 531 participants and around 265 per treatment. Using four treatments would suggest a total sample size of 1060 participants. As

we are planning to perform four repetitions per target in the pilot study, the final power is likely to be higher. Therefore, we focus on a final sample size of 800 participants (200 per condition), resulting in a total of 6400 observations.

Final Sample. We recruited 819 participants located in the UK via Prolific.co for a twenty-minute study.[1] Participants received a base payment of £2 and were able to earn an additional bonus payment of maximally £4 based on their own and another participants' performance (M = 2.43, SD = .55). As registered, we excluded participants that did not understand the mind-shape task, failed an attention check, or were below the age of 18 (n = 0), and participants that did not report the correct minimal group of the other participant (n = 17). When inspecting the sample, we also noticed that a small proportion did not correctly commit to the oath (e.g., by either leaving the text field blank or falsely pasting their participant ID, n = 17), did not state the correct art preference based on the ART (e.g., by naming a different artist, n = 15), or provided duplicates of the same participants based on the ID assigned by the crowdsourcing service (n = 9). Although we did not register to apply these exclusion criteria, they seemed as sensible measures to exclude participants that did not pay enough attention. We provide sensitivity analyses including these cases below.

The final sample consisted of 768 participants (385 men, 378 women, 3 other, 2 prefer not to say; 703 UK nationals, 65 other) ranging from 18 to 80 years of age (M = 41.22, SD = 13.44).

Procedure

In the present study we employed a 4 (social commitment – between: baseline, minimal group (MG), oath, MG+oath) x 2 (target – within: self, other) mixed design. After providing informed consent, participants were randomly allocated to one out of four different treatments (*baseline*: n = 198). Participants in the *oath* (n = 188) and *MG+oath* (n = 188) treatments were asked to commit to an honesty oath directly following the information letter. Participants in all conditions then completed the Artistic Preference Task (ART[4]), which consisted of selecting a painting of five pairs of paintings by the two artists Klee and Kandinsky. Participants in the *MG* (n = 194) and *MG*+oath treatment, were then presented with their artistic preference based of which painter they selected three or more paintings (Kandinsky: n = 254; Klee: n = 128). In order to strengthen their group identification, participants were then asked to type in the name of their preferred painter.

[1]As registered, we paused data collection after reaching ~100 participants and inspected distribution of responses to the mind-shape task in order to validate that cheating (non-normal distribution) was present.

Afterwards, participants read the instructions of the mind-shape task. Comprehension was probed with an item. Participants failing the item ($n = 20$) were shown the instructions once more and could not advance if the failed the comprehension item again. Participants then played two rounds of the mind-shape task. Each round consisted of four trials. The rounds were presented as resulting in an additional payment based on the performance for either the self or another participant from the same experiment. Individuals were told that they did not interact with the other participant, but were only matched after the experiment was terminated. In the *MG* and *MG+oath* treatments, we emphasized that the other participant had the same artistic preference (i.e., Klee or Kandinsky) based on the ART. Participants then completed items regarding the mind-shape task and the other participants and two personality questionnaires assessing honesty-humility and self-control in individualized random order. Then, participants provided information on their gender, age, nationality, and partial postcode. Finally, participants engaged in a similarity rating task using a spatial arrangement method (Q-SpAM[5]).

Materials

Oath Task. Following previous studies[6,7], participants were asked to commit to the statement "Participants in this study commit to the norm of telling the truth. I promise that the information I am providing in this study is true." The first sentence was added in order to emphasize a social norm of honesty among the participants. As signing in online studies is difficult (also considering privacy concerns), participants were asked to write the second part of the statement into a text field[6]. It was not possible for participants to just copy the statement and they had to actively type it in to strengthen commitment. In addition, participants were not forced to type the statement (i.e., they could advance without typing it). This was done as previous research emphasizes that voluntary actions strengthen commitment [8]. In total, 389 participants committed to the oath by copying the statement (95.81% compliance). Two participants did not commit, two just wrote 'Yes', and thirteen participants falsely added their Prolific ID. After applying the exclusion criteria, 378 participants committed to the oath, while two did not (99.47% compliance).

ART Task. In Study 1, we manipulated commitment to the partner by using the ART task[4,9], as done in previous studies (e.g., Chen & Li, 2009; Jiang, 2014; Rong et al., 2016). Participants were presented with five pairs of paintings, each pair including a painting by Paul Klee and Wassily Kandinsky and were asked to select which one they preferred. Paintings were randomly selected for each pair from a pool of 12 paintings by each artist (based on Hong & Ratner, 2021). Avoiding deception, in two treatments (*partner,*

partner+oath), participants were told their actual artistic preference (based on the painter they chose three or more paintings from), in the remaining treatments, the task was completed as a filler task and participants did not know about their actual preference. To manipulate commitment to the partner in the *partner* and *partner+oath* treatments participants were informed that their matched partner shared the same preference as they did prior to the economic game task resulting in payments for the other. Participants did not interact and actual matching was only performed after the study. The ART task has been used in previous studies to investigate dishonesty in relation to commitment [10,12]

Measures

Mind-Shape Task. In order to measure dishonesty behavior, we adapted a modified mind game task[13]. In such mind game paradigms, participants are typically asked to think of a certain outcome, then presented with an actual outcome, and asked whether their imagined outcome matches the actual outcome. As individuals are incentivized if both outcomes match, there exists a propensity to cheat. In addition, cheating is non-detectable as it occurs in private, which is one reason that mind game paradigms might induce higher cheating rates[14]. Here, we introduce the mind-shape task, a mind game task with an ordinal payoff structure. In the first part of the task, participants are presented with six different shapes. In the present study, these were randomly taken from a pool of 11 shapes at each trial. The participant is asked to pick one shape privately by thinking about it or writing it down in private. In the second part, each of the six shapes is randomly associated with a payoff from 0 to 1 (in the present study: 0p, 10p, 20p, 30p, 40p, 50p). The participant then has to indicate which shape they choose. This task allows for cheating as individuals can choose a shape with a higher payoff than original selected one in order to maximize their payoff. As other mind games, the task does not allow for measuring actual cheating at the individual level, but mainly cheating on the average level, depending on the distribution of responses. Expecting each shape having the same probability of being associated with a certain payoff, we can explore whether responses exceed the expected mean value of 3.5 (assuming a scale from 1 to 6).

There are several advantages to this task. First, previous research has found that dishonesty increases when occurring in private [14]. The mind game is completely private and actual selections cannot be recorded. The original mind game [13] features the disadvantage that it only includes a binary outcome (participants either win or not), which reduces overall power of the measure and also makes dishonesty an all or nothing decision (you can either cheat or not). We combine the mind game with a payoff scheme similar to the die-roll task [15]. Importantly, the mind-shape paradigm does not need external applications to mimic a die roll

and relies on basic geometric shapes that are recognized across cultures [16–18]. An overview of the shapes is provided in Supplementary Figure 1.

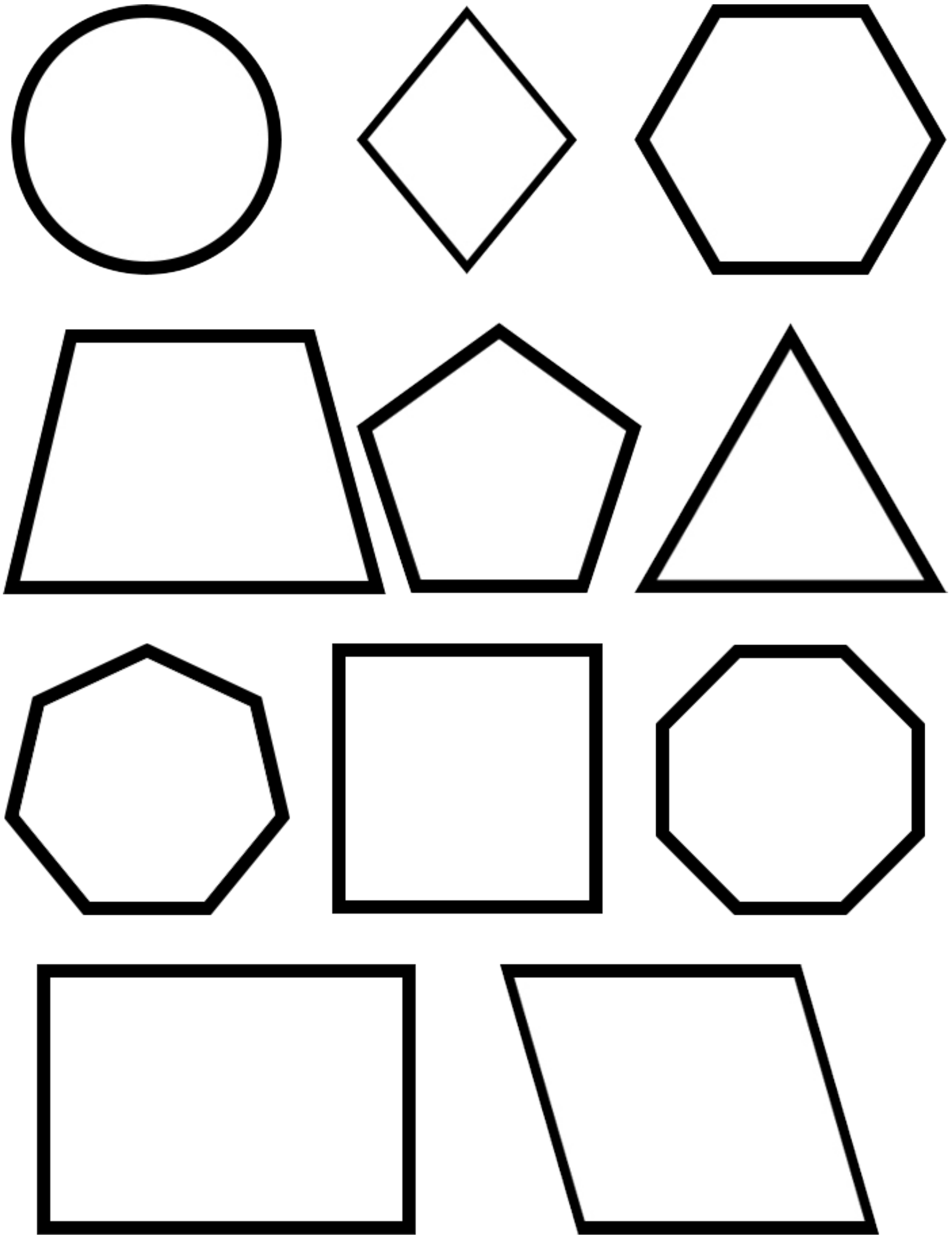

Supplementary Figure 1. Overview of geometric shapes used in the mind-shape task. All shapes feature the same height. In each trial, six shapes were randomly selected from this pool.

Closeness to Other. We measured closeness to the other person, by using the inclusion-of-the-other-in-the-self scale (IOS; [19]). The IOS item depicts seven Venn-like diagrams with two circles representing the self and the other increasing in overlap.

Responsibility. One possible mechanism could be that felt moral responsibilities differs between cheating for others and cheating after taking an oath. We assessed responsibility by asking participants "How much responsibility did you feel for your actions during the mind-shape task" on a 5-point scale from "none at all" to "a great deal".

We also added two attention check questions. One question asked participants to check a certain response option. Another item asked participants to indicate the artistic preference of the other participant (only shown in the *MG* and *MG+oath* treatments).

Personality Measures. We employed the 10-item honesty-humility scale [20] on a 5-point scale from "strongly disagree" to "strongly agree". Similarly, participants completed the 12-item self-control scale [21] on a 5-point scale from "not at all like me" to "very much like me".

Spatial Arrangement Similarity Rating (Q-SpAM). We adapted the Q-SpAM paradigm [5], a spatial arrangement task to study similarity ratings among categories. Participants were asked to arrange the eleven shapes employed in the experiment by dragging them across their entire screen, arrange more similar shapes closer to each other and more dissimilar shapes more distant from each other.

1.2 Study 2

Method

Participants

Sample Size Justification. Based on a previous meta-analysis [1] effects of effort/investment manipulations on dishonesty (H1) were g = -.16 [-.31, -.01], while effects of oath paradigms on dishonesty (H2) were g = .26 [.15, .37]. Because power calculations for ordinal logistic regressions are complex, we focused on the smallest effect size (H1) and calculated the power for the two-way comparison using the *posamsize* function in the *hmisc* package [2]. We used the frequencies of the distribution of payoffs (mind shape from 1 to 6) based on data from our first pilot study. Focusing on an odds ratio of .748 (transformed from a g = -.16), an alpha level of .05, and a power of .80, we observed a full sample size of 1160.5 participants and 580 per treatment. Using four treatments would suggest a total sample size of 2320 participants. Given the nature of the pilot and that each participants provides four data points, we focus on a final sample size of 1000 participants (250 per condition), resulting in a total of 4000 observations.

Final Sample. We recruited 1,587[2] participants located in the UK via the panel company Toluna for a five-minute study. Participants received a base payment of 1200 Toluna points (approx. $0.4) and were able to earn an additional bonus payment of maximally 1200 points based on their own and another participants' performance (*M* = 652.10, *SD* = 204.82). As registered, we excluded participants that were below the age of 18 (*n* = 2), and participants that spent less than one-third of the median of time participants spent on the survey or more than three times the median (*n* = 67). When inspecting the sample, we also noticed that a small proportion did not correctly commit to the oath (e.g., by falsely pasting their participant ID, *n* = 24). Although we did not register to apply this exclusion criterion, it seemed as sensible measures to exclude participants that did not pay enough attention. We provide sensitivity analyses including these cases below.

The final sample consisted of 1,494 participants (594 men, 893 women, 6 other, 1 prefer not to say; 1390 UK nationals, 93 other) ranging from 18 to 91 years of age (*M* = 53.66, *SD* = 15.72).

Procedure

In the present study we employed a 4 (social commitment – between: baseline, partner, oath, partner+oath) x 2 (task – between: mind shape, die roll) design. After providing informed consent, participants completed items on their gender, age, and region. Participants were randomly allocated to one out of four different treatments (*baseline*: *n* = 415; *partner*: *n* = 373). Participants in the *oath* (*n* = 368) and *partner+oath* (*n* = 338) treatments were asked to commit to an honesty oath directly before starting the main task. In addition, participants were randomly allocated to perform one out of two possible tasks (*mind shape*: *n* = 788; *die roll*: *n* = 706).

Afterwards, participants read the instructions of the specific task. Comprehension was probed with an item. Participants failing the item (*n* = 20) were screened out. Participants then played one round of the specific task that consisted of four trials. The task was presented as resulting in an additional payment based on the participant's performance or the participant's and the partner's performance in the *partner* and *partner+oath* treatments. Participants in the *partner* and *partner+oath* treatments completed two items regarding commitment towards the partner before and after the main task. In all treatments, participants completed a

[2] Originally, we recruited 2,251 participants of which 576 were screened out and did not complete the survey and 88 provided partial responses. Participants were screened out if they did not provide informed consent, were excluded because they did not understand the instructions of the task or because they failed an attention check.

personality questionnaire assessing honesty-humility. Finally, participants provided information on their nationality, and socioeconomic status and were debriefed.

Incentive Scheme. Following previous research [22,23], we manipulated social commitment by implementing two different incentive schemes. In each trial, participants could earn between 0 and 300 points (0, 60, 120, 180, 240, 300) dependent on the reported geometric shape or die roll. This resulted in a possible maximum of 1200 bonus points. In the *baseline* and *oath* treatments, participants received a bonus payment based on their reported performance in the specific task. Following, the *individual* treatment in [22], final points were equal to the final payoff ($p_i = \pi_i$). In the *partner* and *partner+oath* treatments, we adapted the *team* condition from [22]. Participants were randomly matched with another participant from the same treatments (either *partner* or *partner+oath*) and the individual payoffs were defined according to the following rule: $\pi_i = 1/2 \cdot (p_i + p_j)$. Payoffs were the same for both participants ($\pi_i = \pi_j$) and participants did not actively interact (i.e., were not able to communicate) but were only randomly matched after the experiment.

Materials

Oath Task. Following previous studies [6,7], participants were asked to commit to the statement "Participants in this study commit to the norm of telling the truth. I promise that the information I am providing in this study is true." The first sentence was added in order to emphasize a social norm of honesty among the participants. As signing in online studies is difficult (also considering privacy concerns), participants were asked to write the second part of the statement into a text field [6]. It was not possible for participants to just copy the statement and they had to actively type it in to strengthen commitment. In addition, participants were not forced to type the statement (i.e., they could advance without typing it). This was done as previous research emphasizes that voluntary actions strengthen commitment [8]. In total, 1,333 participants committed to the oath by copying the statement (94.34% compliance). Fifty-six participants did not commit, fourteen just wrote 'Yes', two stated that they agree, two stated that it is ok, one person wrote their name, and five participants falsely added the survey URL (n = 80 did not commit).

Measures

Mind-Shape Task. In order to measure dishonesty behavior, we adapted a modified mind game task [13]. In such mind game paradigms, participants are typically asked to think of a certain outcome, then presented with an actual outcome, and asked whether their imagined outcome matches the actual outcome. As individuals are incentivized if both outcomes match, there exists a propensity to cheat. In addition, cheating is non-detectable as it occurs in

private, which is one reason that mind game paradigms might induce higher cheating rates [14]. Here, we introduce the mind-shape task, a mind game task with an ordinal payoff structure. In the first part of the task, participants are presented with six different shapes. In the present study, these were randomly taken from a pool of 11 shapes at each trial. The participant is asked to pick one shape privately by thinking about it or writing it down in private. In the second part, each of the six shapes is randomly associated with a payoff from 0 to 300 (in the present study: 0pts, 60pts, 120pts, 180pts, 240pts, 300pts). The participant then has to indicate which shape they choose. This task allows for cheating as individuals can choose a shape with a higher payoff than original selected one in order to maximize their payoff. As other mind games, the task does not allow for measuring actual cheating at the individual level, but mainly cheating on the average level, depending on the distribution of responses. Expecting each shape having the same probability of being associated with a certain payoff, we can explore whether responses exceed the expected mean value of 3.5 (assuming a scale from 1 to 6).

Die Roll. To test the validity of the mind-shape task, a portion of participants completed a die roll task based on previous studies [15,22]. Participants were in each trial ask to privately roll a die by either using an actual die or using an external website. We emphasized that responses from the external website could not be linked to our survey, so die rolls were occurring in private. After rolling the die, participants were asked to report number of pips they rolled with each being associated with a different payoff (1 = 0pts, 2 = 60pts, 3 = 120pts, 4 = 180pts, 5 = 240pts, 6 = 300pts), leaving the possibility for participants to increase their payoff by misreporting the die roll. The die roll paradigm has been employed and validated as a measure of dishonesty across various contexts [14].

Commitment. We measured commitment to the other person in the *partner* and *partner+oath* treatments, by using the inclusion-of-the-other-in-the-self scale (IOS) and an item asking how committed participants felt to their partner on a 7-point scale from 0 – not at all committed to 6 – very much committed, right before and after the dishonesty task. The IOS item depicts seven Venn-like diagrams with two circles representing the self and the other increasing in overlap.

Responsibility. One possible mechanism could be that felt moral responsibilities differs between cheating for others and cheating after taking an oath. We assessed responsibility by

asking participants "How much responsibility did you feel for your actions during the mind-shape task" on a 5-point scale from "none at all" to "a great deal".[3]

Honesty-Humility. We employed the 10-item honesty-humility scale ($\alpha = .74$; [20]) on a 5-point scale from "strongly disagree" to "strongly agree". We also added one attention check item among the honesty-humility items. Participants failing this attention check were screened out ($n = 31$).

1.3 Study 3

Method

Participants

Sample Size Justification. Based on Pilot I and Pilot II the most consistent effect of the oath was OR = .85. Because power calculations for ordinal logistic regressions are complex, we focused on the smallest effect size (H2) and calculated the power for the two-way comparison using the *posamsize* function in the *hmisc* package [2]. We used the frequencies of the distribution of payoffs (mind shape from 1 to 6) based on data from our first pilot study (because this study also was recruited on Prolific, while Pilot II was not). Focusing on an odds ratio of .85, an alpha level of .05, and a power of .80, we observed a full sample size of 3704 participants and 1852 per treatment. Using four treatments would suggest a total sample size of 7408 participants. Given the nature of the pilot and that each participant provides ten data points, we focus on a final sample size of 500 participants (125 per condition), resulting in a total of 5000 observations.

Final Sample. We recruited 509 participants located in the UK via the crowdsourcing website Prolific.co for a ten-minute study. Participants received a base payment of £1 and were able to earn an additional bonus payment of maximally £2 based on their own and another participants' performance (M = £1.19, SD = £.27). As registered, we excluded participants that did not understand the were below the age of 18 ($n = 0$), participants that spent less than one-third of the median of time participants spent on the survey or more than three times the median ($n = 15$), and participants that did not correctly commit to the oath (e.g., by pasting their Prolific ID, $n = 1$). In addition, participants were excluded if they failed a comprehension question about the instructions ($n = 4$) and an attention check embedded among the honesty-humility scale ($n = 1$). Similarly, $n = 4$ participants were screened out because they did not consent participating in the study.

[3] Due to an error this item was only assessed in the *partner* and *partner+oath* treatments.

The final sample consisted of 484 participants (240 men, 242 women, 2 other; 435 UK nationals, 49 other) ranging from 18 to 78 years of age (*M* = 39.81, *SD* = 13.25).

Procedure

In general, Pilot III followed the main procedures of Pilot II with a few exceptions. We again employed a 4 (social commitment – between: baseline, partner, oath, partner+oath) x 2 (task – between: mind shape, die roll) design. After providing informed consent, participants were randomly allocated to one out of four different treatments (*baseline*: *n* = 123; *partner*: *n* = 135). Participants in the *oath* (*n* = 117) and *partner+oath* (*n* = 109) treatments were asked to commit to an honesty oath directly before starting the main task. In addition, participants were randomly allocated to perform one out of two possible tasks (*mind shape*: *n* = 252; *die roll*: *n* = 232).

Afterwards, participants read the instructions of the specific task. Comprehension was probed with an item. Participants failing the item (*n* = 4) were screened out. Participants then played one round of the specific task that consisted of ten trials (instead of four as in Pilot II). The task was presented as resulting in an additional payment based on the participant's performance or the participant's and the partner's performance in the *partner* and *partner+oath* treatments. Participants in the *partner* and *partner+oath* treatments completed two items regarding commitment towards the partner before and after the main task. In all treatments, participants completed a personality questionnaire assessing honesty-humility. Finally, participants provided information on their gender, age, region, nationality, socioeconomic status, partial postcode, and were debriefed.

Incentive Scheme. Following previous research [22,23], we manipulated social commitment by implementing two different incentive schemes. In each trial, participants could earn between 0p and 20p (0p, 4p, 8p, 12p, 16p, 20p) dependent on the reported geometric shape or die roll. This resulted in a possible maximum of £2. In the *baseline* and *oath* treatments, participants received a bonus payment based on their reported performance in the specific task. Following, the *individual* treatment in [22], final points were equal to the final payoff ($p_i = \pi_i$). In the *partner* and *partner+oath* treatments, we adapted the *team* condition from [22]. Participants were randomly matched with another participant from the same treatments (either *partner* or *partner+oath*) and the individual payoffs were defined according to the following rule: $\pi_i = 1/2 \cdot (p_i + p_j)$. Payoffs were the same for both participants ($\pi_i = \pi_j$) and participants did not actively interact (i.e., were not able to communicate) but were only randomly matched after the experiment.

Materials

Oath Task. Following previous studies [6,7], participants were asked to commit to the statement "Participants in this study commit to the norm of telling the truth. I promise that the information I am providing in this study is true." The first sentence was added in order to emphasize a social norm of honesty among the participants. As signing in online studies is difficult (also considering privacy concerns), participants were asked to write the second part of the statement into a text field [6]. It was not possible for participants to just copy the statement and they had to actively type it in to strengthen commitment. In addition, participants were not forced to type the statement (i.e., they could advance without typing it). This was done as previous research emphasizes that voluntary actions strengthen commitment [8]. In total, 482 participants committed to the oath by copying the statement (99.59% compliance). Two participants did not commit and one person indicated their Prolific ID.

Measures

Mind-Shape Task. In order to measure dishonesty behavior, we adapted a modified mind game task (Jiang, 2013). In such mind game paradigms, participants are typically asked to think of a certain outcome, then presented with an actual outcome, and asked whether their imagined outcome matches the actual outcome. As individuals are incentivized if both outcomes match, there exists a propensity to cheat. In addition, cheating is non-detectable as it occurs in private, which is one reason that mind game paradigms might induce higher cheating rates (Gerlach et al., 2019). Here, we introduce the mind-shape task, a mind game task with an ordinal payoff structure. In the first part of the task, participants are presented with six different shapes. In the present study, these were randomly taken from a pool of 11 shapes at each trial. The participant is asked to pick one shape privately by thinking about it or writing it down in private. In the second part, each of the six shapes is randomly associated with a payoff from 0p to 20p (in the present study: 0p, 4p, 8p, 12p, 16p, 20p). The participant then has to indicate which shape they choose. This task allows for cheating as individuals can choose a shape with a higher payoff than original selected one in order to maximize their payoff. As other mind games, the task does not allow for measuring actual cheating at the individual level, but mainly cheating on the average level, depending on the distribution of responses. Expecting each shape having the same probability of being associated with a certain payoff, we can explore whether responses exceed the expected mean value of 3.5 (assuming a scale from 1 to 6).

Die Roll. To test the validity of the mind-shape task, a portion of participants completed a die roll task based on previous studies (Conrads et al., 2013; Fischbacher & Föllmi-Heusi, 2013).

Participants were in each trial ask to privately roll a die by either using an actual die or using an external website. We emphasized that responses from the external website could not be linked to our survey, so die rolls were occurring in private. After rolling the die, participants were asked to report number of pips they rolled with each being associated with a different payoff (1 = 0p, 2 = 4p, 3 = 8p, 4 = 12p, 5 = 16p, 6 = 20p), leaving the possibility for participants to increase their payoff by misreporting the die roll. The die roll paradigm has been employed and validated as a measure of dishonesty across various contexts (Gerlach et al., 2019).

Commitment. We measured commitment to the other person in all treatments, by using the inclusion-of-the-other-in-the-self scale (IOS; Aron et al., 1992) and an item asking how committed participants felt to their partner or to another participant in the study (in the baseline and oath treatments) on a 7-point scale from 0 – not at all committed to 6 – very much committed, right before the dishonesty task. The IOS item depicts seven Venn-like diagrams with two circles representing the self and the other increasing in overlap. We also included the two items after the dishonesty task in the partner and partner+oath treatments.

Responsibility. One possible mechanism could be that felt moral responsibilities differs between cheating for others and cheating after taking an oath. We assessed responsibility by asking participants "How much responsibility did you feel for your actions during the mind-shape task" on a 5-point scale from "none at all" to "a great deal".

Honesty-Humility. We employed the 10-item honesty-humility scale ($\alpha = .74$; Ashton & Lee, 2009) on a 5-point scale from "strongly disagree" to "strongly agree". We also added one attention check item among the honesty-humility items. Participants failing this attention check were screened out ($n = 1$).

1.4 Study 4 & 5

Method

Participants

Sample Size Justification. Based on Pilot III the most consistent effect of the oath was OR = .74. Because power calculations for ordinal logistic regressions are complex, we focused on this smallest effect size (H2) and calculated the power for the two-way comparison using the *posamsize* function in the *hmisc* package (Harell Jr. & Harell Jr., 2019). We used the frequencies of the distribution of payoffs (mind shape from 1 to 6) based on data from Pilot III (which was also recruited on Prolific). Focusing on an odds ratio of .74, an alpha level of

.05, and a power of .80, we observed a full sample size of 1081.4 participants and ~540 per treatment. Using four treatments would suggest a total sample size of 2160 participants. Given that half of participants provide four data points, we will again focus on a somewhat smaller effect size. In order to take advantage of the improved commitment manipulation (live interaction), we employed a sequential analysis for the current data [24]. In a first step, we recruited 400 participants (100 per condition) and tested whether the effect in H1/H2 has reached statistical significance. As this was not the case we recruited another 400 participants (800 in total, 200 per condition) and checked the results again. H1 failed to reach statistical significance and we recruited another 400 participants (1200 in total, 300 per condition). Finally, H1 still showed no statistically significant results and we recruited a final 400 participants (1600 in total, 400 per condition) ending the recruitment process. This final recruitment step resulted in 4000 observations, which is far above the suggested total sample size of 2160 participants. In addition, effects in the range of our smallest effect size of interest (*d* = +/- .15) should reach statistical significance at this stage. We controlled for the Type-1 error rate by adjusting the alpha level at each step based on a spending function using the GroupSeq package in R (see https://osf.io/qtufw/; [25]. We selected 4 interim times, two-sided bounds, alpha level at .05, and a power familiy function. The spending fuction suggested an alpha level of .0125, .0161, .0203, .0248 at the four different times.

Final Sample.

UK Sample. We recruited 1684 participants located in the UK via the crowdsourcing website Prolific.co for a ten-minute study in four different batches as explained above.[4] Participants received a base payment of £1 and were able to earn an additional bonus payment of maximally £2 based on their own and another participants' performance. In total we paid £2126 out of £3142 in possible bonuses (*M* = £1.35, *SD* = £0.56). As registered, we excluded participants that did not understand the instructions (*n* = 39), that were below the age of 18 (*n* = 0), participants that spent less than one-third of the median of time participants spent on the survey or more than three times the median (*n* = 61), did not correctly commit to the oath (e.g., by pasting their Prolific ID, *n* = 2) and failed an attention check embedded among the honesty-humility scale (*n* = 2). In addition, we also excluded partial responses (*n* = 32) and *n* = 42 cases because they presented the same participant retaking the study.[5]

[4] The first two batches were recruited on two subsequent days. The last two batches were recruited three days later on the same day. Participants were able to only participate in one batch.

[5] This was identified by screening participants IDs. Based on feedback one reason for participants retaking the study was a redirection error at the end of the study in batch two and three. This did not affect the main

The final sample consisted of 1541 participants (775 men, 750 women, 9 non-binary, 7 prefer not to say; 1425 UK nationals, 116 other) ranging from 18 to 93 years of age (*M* = 41.24, *SD* = 13.84).

Mexican Sample. We recruited 1077 Mexican participants via Prolific.co in three batches as explained earlier. We stopped during the third wave because we were not able to collect enough participants at the same time, which was essential for the interactive nature of the task leading to a high number of drop-outs. Participants received a base payment of £1 and were able to earn an additional bonus payment of maximally £2 based on their own and another participants' performance. In total we paid £1317.2 (66.06%) out of £1994 in possible bonuses (*M* = £1.32, *SD* = £.53). As registered, we excluded participants that did not understand the instructions (*n* = 33), that were below the age of 18 (*n* = 0), participants that spent less than one-third of the median of time participants spent on the survey or more than three times the median (*n* = 38), did not correctly commit to the oath (e.g., by pasting their Prolific ID, *n* = 1) and failed an attention check embedded among the honesty-humility scale (*n* = 0).

The final sample consisted of 982 participants (478 men, 480 women, 22 non-binary, 2 prefer not to say; 966 Mexican nationals, 16 other) ranging from 18 to 63 years of age (*M* = 25.66, *SD* = 6.49).

Procedure

In general, the study followed the main procedures of Pilot II and III with a few notable exceptions. We employed a 4 (social commitment – between: baseline, partner, oath, partner+oath) x 2 (type of task – between: four trial lottery, one-shot) between subject design. After providing informed consent, participants were randomly allocated to one out of four different treatments (Study 4: *baseline*: *n* = 432, Study 5: *baseline*: *n* = 254; Study 4: *partner*: *n* = 366; Study 5: *partner*: *n* = 231). Participants in the *oath* (Study 4: *n* = 406; Study 5: *n* = 257) and *partner+oath* (Study 4: *n* = 337; Study 5: *n* = 240) treatments were asked to commit to an honesty oath directly before starting the main task. In addition, participants were randomly allocated to perform one out of two possible versions of the mind-shape task determining the bonus payment of participants (Study 4: four trial lottery, *n* = 785, one-shot, *n* = 756; Study 5: four trial lottery, *n* = 503, one-shot, *n* = 479).

---

responses, but we excluded participants if they retook the experiment because they had likely read the debriefing information and to keep responses independent.

Afterwards, participants read the instructions of the specific task. Comprehension was probed with an item. Participants failing the item (Study 4: $n = 37$; Study 5: $n = 33$) were screened out. In the *partner* and *partner+oath* treatments, participants were matched with another partner and engaged in a chat for up to 3-minutes before the main task with the objective of deciding on a group name for the dyad. Participants then played the mind-shape task with either one or four trials. The task was presented as resulting in an additional payment based on the participant's performance or the participant's and the partner's performance in the *partner* and *partner+oath* treatments. Participants in all treatments completed two items regarding commitment towards the partner (*partner, partner+oath*) or someone else from the experiment (*baseline*, *oath*) before and after the main task. In all treatments, participants completed a personality questionnaire assessing honesty-humility. Finally, participants provided information on their gender, age, region, nationality, socioeconomic status, partial postcode, and were debriefed.

Incentive Scheme & Type of Task. Following previous research [22,23] we manipulated social commitment by implementing two different incentive schemes. In each version of the game, participants could earn between £0 and £2 (£0, £0.4, £0.8, £1.2, £1.6, £2) dependent on the reported geometric shape. In the *four trial lottery* version participants completed four trials of the mind-shape task and one responses was randomly chosen to determine the bonus. In the *one-shot* version, participants played one trial of the task, which determined participant's bonus. In the *baseline* and *oath* treatments, participants received a bonus payment based on their reported performance in the specific task. Following, the *individual* treatment in [22] final points were equal to the final payoff ($p_i = \pi_i$). In the *partner* and *partner+oath* treatments, we adapted the *team* condition from [22]. Participants were randomly matched with another participant from the same treatments (either *partner* or *partner+oath*) and the individual payoffs were defined according to the following rule: $\pi_i = 1/2 \cdot (p_i + p_j)$. Payoffs were the same for both participants ($\pi_i = \pi_j$) and participants actively interacted before the main task.

Live Interaction. We implemented a live matching and interaction in the *partner* and *partner+oath* treatments using the SMARTRIQS framework [26]. Participants in these treatments were randomly matched with another participant from one of the same treatments, which determined their partner for the rest of the study.[6] Matching participants could take up to 3 minutes and the survey was terminated if no other participant could be matched at the

[6] Note that participants were not able to tell whether they were in the *partner* or *partner+oath* condition as the only difference between conditions (the oath) was implemented after the partner chat.

same time. After being successfully matched, participants received instructions that they would engage in a chat for up to 3-minutes with their partner and that they could exit the chat before the time was up.[7] In order to strengthen commitment to the partner, participants received the task to come up with a group name during the chat. This procedure has been successfully implemented in previous studies to generate increased commitment among individuals [11]. After completing the chat, participants were asked to write down their group name. This name was used for subsequent instructions when referring to the shared payoff scheme. Participants then completed the mind-shape game. After the game participants were informed about their own, their partner's, and the final (their group's) bonus payment. Participants were not able to communicate during the main task. If participants timed out (e.g., by leaving the survey or not responding) during the first stage of interaction (i.e., the group chat), their partner was not able to proceed with the study, but the survey was terminated. If participants time out during the second stage of interaction (i.e., the mind-shape task) a default response was generated by randomly picking one out of the six possible answers and this was used to determine the group's final bonus. The remaining participant was then informed that their partner timed out and that the final response was randomly generated. Of all initiated dyads few were terminated early: Study 4: 4.37% 16/366; Study 5: 2.5% 6/240.

Oath Task. Following previous studies [6,7], participants were asked to commit to the statement "Participants in this study commit to the norm of telling the truth. I promise that the information I am providing in this study is true." The first sentence was added in order to emphasize a social norm of honesty among the participants. As signing in online studies is difficult (also considering privacy concerns), participants were asked to write the second part of the statement into a text field [6]. It was not possible for participants to just copy the statement and they had to actively type it in to strengthen commitment. In addition, participants were not forced to type the statement (i.e., they could advance without typing it). This was done as previous research emphasizes that voluntary actions strengthen commitment [8]. In Study 4, 741 participants committed to the oath by copying the statement (99.73% compliance). Two participants did not commit. In Study 5, 496 participants committed to the oath by copying the statement (99.80% compliance), one participant did not commit.

[7] Due to a technical error chat duration was not recorded. Based on chatlogs it is highly likely that the majority of dyads exited the chat before the three minutes were up.

Measures

Mind-Shape Task. In order to measure dishonesty behavior, we adapted a modified mind game task [13]. In such mind game paradigms, participants are typically asked to think of a certain outcome, then presented with an actual outcome, and asked whether their imagined outcome matches the actual outcome. As individuals are incentivized if both outcomes match, there exists a propensity to cheat. In addition, cheating is non-detectable as it occurs in private, which is one reason that mind game paradigms might induce higher cheating rates [14]. Here, we introduce the mind-shape task, a mind game task with an ordinal payoff structure. In the first part of the task, participants are presented with six different shapes. In the present study, these were randomly taken from a pool of 11 shapes at each trial. The participant is asked to pick one shape privately by thinking about it or writing it down in private. In the second part, each of the six shapes is randomly associated with a payoff from £0 to £2 (in the present study: £0, £0.4, £0.8, £1.2, £1.6, £2). The participant then has to indicate which shape they choose. This task allows for cheating as individuals can choose a shape with a higher payoff than original selected one in order to maximize their payoff. As other mind games, the task does not allow for measuring actual cheating at the individual level, but mainly cheating on the average level, depending on the distribution of responses. Expecting each shape having the same probability of being associated with a certain payoff, we can explore whether responses exceed the expected mean value of 3.5 (assuming a scale from 1 to 6).

Commitment. We measured commitment to the other person in all treatments, by using the inclusion-of-the-other-in-the-self scale (IOS) and an item asking how committed participants felt to their partner or to another participant in the study (in the baseline and oath treatments) on a 7-point scale from 0 – not at all committed to 6 – very much committed, right before the dishonesty task. The IOS item depicts seven Venn-like diagrams with two circles representing the self and the other increasing in overlap. We also included the two items after the dishonesty task in the partner and partner+oath treatments.

Responsibility. One possible mechanism could be that felt moral responsibilities differs between cheating for others and cheating after taking an oath. We assessed responsibility by asking participants "How much responsibility did you feel for your actions during the mind-shape task" on a 5-point scale from "none at all" to "a great deal".

Honesty-Humility. We employed the 10-item honesty-humility scale (Study 4: α = .74; Study 5: α = .69; [20]) on a 5-point scale from "strongly disagree" to "strongly agree". We also

added one attention check item among the honesty-humility items. Participants failing this attention check were screened out (Study 4: $n = 2$; Study 5: $n = 0$).

1.5 Study 6

Participants

Sample Size Determination. Based on Study 4 the most consistent effect of the oath was OR = .72. Because power calculations for ordinal logistic regressions are complex, we focused on this smallest effect size (H2) and calculated the power for the two-way comparison using the *posamsize* function in the *hmisc* package [2]. We used the frequencies of the distribution of payoffs (mind shape from 1 to 6) based on data from Study 4 (which was also recruited on Prolific, but with an UK sample). Focusing on an odds ratio of .72, an alpha level of .05, and a power of .80, we observed a full sample size of 920.7 participants and ~460 per treatment. Using four treatments would suggest a total sample size of 1840 participants. In order to take advantage of the improved commitment manipulation (live interaction), we will employ a sequential analysis for the current data (Lakens, 2014). In a first step, we will recruit 400 participants (100 per condition) and test whether the effect in H1/H2 has reached statistical significance. If that is not the case we will recruit another 400 participants (800 in total, 200 per condition) and check the results again. If it has failed to reach statistical significance, we will recruit another 400 participants (1200 in total, 300 per condition). We will repeat this process if we don't reach statistical significance and recruit another 400 participants (1600 in total, 400 per condition) when we end the recruitment process. This final recruitment step would result in 1600 observations, which would be close to the suggested sample size. In addition, effects in the range of our smallest effect size of interest (d = +/- .15) should reach statistical significance at that stage. We will control for the Type-1 error rate by adjusting the alpha level at each step based on a spending function using the GroupSeq package in R (see https://osf.io/qtufw/). Selecting 4 interim times, two-sided bounds, alpha level at .05, and a power familiy function. The spending fuction suggested an alpha level of .0125, .0161, .0203, .0248 at the four different times.

Final Sample. Contrary to our registration we stopped recruitment after the second wave, as it was obvious that effects were not in the expected duration and to save resources. We recruited a total of 835 US participants on Prolific.co. Fourty-nine participants did not consent or provided partial responses, 12 participants were faster than three times the median, 18 participants were slower than one-third of the median, one participant indicated an age below 18, none participant failed to provide the correct oath, or correctly answer the attention checks.

The final sample consisted of 755 US participants (369 women, 11 non-binary, 5 not specified) ranging from 18 to 78 years of age ($M = 39.46$, $SD = 12.82$). A total of 722 reported a US nationality.

Design & Procedure

The study featured a 4 (between - social commitment: baseline (self-only), partner (self-and-other), oath, partner+oath) x 2 (nested in partner/partner+oath: between – type of partner manipulation: shared payoff vs. double) design. Participants were randomly assigned to a baseline ($n = 237$), partner ($n = 164$), oath ($n = 191$), or partner+oath ($n = 163$) treatment. In the partner and partner+oath treatments, participants were assigned to either a shared payoff scheme based on Studies 1-5 ($n = 159$) or a double payoff scheme ($n = 168$).

Participants were presented with information about the study and instructions that they will engage in one round of the die-roll game. Participants in the partner and partner+oath task were told that they would be matched with someone from the study and chat with that partner during the die-roll game.

Baseline. In the self-only condition (baseline), participants were told that their own performance will determine their possible bonus payments. Their reward depended directly on their performance (bonus = participant's rewards).

Oath Task. Following previous studies[6,7], participants were asked to commit to the statement "Participants in this study commit to the norm of telling the truth. I promise that the information I am providing in this study is true" before starting the economic game task. As participants completed the study online, they were asked to type in the statement in a box as done in [6]. It was not possible for participants to copy the statement and they had to actively type it. Participants were not forced to type the statement and could advance without completing this aspect (i.e., typing is a voluntary action, 100% of the retained participants committed to this oath).

Apart from the oath, this condition is the same as the baseline condition. Participant's bonus is also determined by their performance (bonus = participant's rewards).

Partner. In the partner treatment, participants were randomly matched in pairs with another partner from the partner or partner+oath condition who completes the same task. Participants were matched live during the experiment and participated in a chat (up to 2 minutes) after they have rolled the die and before reporting their outcome to discuss the task (based on [27]. In addition, participants were randomly assigned to complete one of two variants of the payoff outcome.

Shared-Payoff. The shared-payoff type was the same as in Study 2-5. Participant's and partner's points were summed and divided by two (bonus = (participant's rewards + partner's rewards)/2).

Double. In the double type participant's bonus payment is dependent on their partner's performance. Participants will receive the respective amount if BOTH participants report the SAME outcome (e.g., both participants report a 5). If participants report different numbers they don't receive any bonus payment (following [3]). The main differences with the die roll paradigm in [3] is that participants rolled the die simultaneously and not sequentially. However, they were able to discuss their die-rolls before reporting them.

Partner Matching. After rolling a die on an external website (random.org), participants engaged in an up to 2-minute live chat with their partner to discuss their outcome and collectively decide on what to report. Participants had the incentive to misreport their number as their final payoff depended on their partner's report. Live interaction was implemented via the SMARTRIQS platform [26]. Participants could be matched with anyone assigned to the partner or partner+oath conditions. They did not know about which condition their partner was assigned to though they could communicate about this during the chat if they wantws to share this information (although this was not their task). Matching occured randomly with a person participating at the exact same time. If one of the matched participants timed out or left the survey after the chat task, their response was be the same as their partner's response. They were assigned this response so that the other participant can still finish the study and provide usable data and in order not to punish the participant because the partner did not follow through on the study.

Partner+Oath. The partner+oath condition was identical with the partner condition except that participants are presented with the oath before engaging in the main task.

Measures

Die Roll Task. The die roll task was adapted from previous studies (e.g., [15]). Participants were asked to roll a die in private. They could either do this by rolling an actual die themselves or going to an external website that simulates a die roll. They were asked to report their die roll outcome. Each outcome was associated with a different bonus payoff (1 = £0, 2 = £0.4, 3 = £0.8, 4 = £1.2, 5 = £1.6, 6 = £2).

Participants were shown a practice example and asked a comprehension question about the task. They were only be able to advance if they answered this question correctly. Participants played a one-shot game in all conditions.

IOS. In the partner and partner+oath conditions, participants completed the inclusion-of-the-other-in-the-self scale (IOS), a 7-point scale with Venn diagrams increasing in overlap right before and after the dishonesty task. In the remaining conditions, this was only completed before the experiment asking about their overlap to a random person participating in the experiment.

Perceived Commitment. In the partner and partner+oath conditions, participants completed an item asking about how committed they feel to their partner on a 7-point scale from 0 – not at all committed to 6 – very much committed right before and after the dishonesty task. In the remaining conditions, this was only completed before the experiment asking about their overlap to a random person participating in the experiment.

Responsibility. All participants completed an item asking "How much responsibility did you feel for your actions during the die-roll task?" on a 5-point scale from "none at all" to "a great deal" after the dishonesty task.

Honesty-Humility We used the 10 item honesty-humility scale ($\alpha = .80$; [20]) on a 5-point scale from "strongly disagree" to "strongly agree". We added an attention check item to the honesty-humility items stating "This is an attention check. Please select "3".

Demographics We added items assessing participants' gender, age, nationality (US vs. other), three items on SES, and childhood SES [28], as well as zip-code.

1.6 Study 7

Participants

Sample Size Determination. Across the previous six studies we found an overall effect of OR = .81 [.73, .90] for the individual-oath treatment, which translates to a $d = -.12$. To consider our resources and our smallest effect size of interest of $d = +/- .15$, we calculate our power at $d = -.15$ (OR = .762). We calculated the power for the two-way comparison using the *posamsize* function in the *hmisc* package [2]. We used the frequencies of the distribution of payoffs from [3]. Focusing on an odds ratio of .762, an alpha level of .05, and a power of .80, we observed a full sample size of 1361.9 participants and 680.5 per treatment. Using four treatments would suggest a total sample size of 2722 participants. In order to save potential resources, we will employ a sequential analysis for the current data [24]. In a first step, we will recruit 800 participants (200 per condition) and test whether the effect in H1/H2 has reached statistical significance. If that is not the case we will recruit another 400 participants (1200 in total, 300 per condition) and check the results again. If it has failed to reach statistical significance, we will recruit another 400 participants (1600 in total, 400 per condition). We will repeat this process if we don't reach statistical significance and recruit another 400 participants (2000 in total, 500 per condition) when we end the recruitment process. Importantly, each participant completes 10 rounds of the main DV and we would reach a total of 20000 observations at the final stage. Effects in the range of our smallest effect size of interest ($d = +/- .15$) should reach statistical significance at that stage. We will control for the Type-1 error rate by adjusting the alpha level at each step based on a spending function using the GroupSeq package in R (see https://osf.io/qtufw/). Selecting 4 interim times, two-sided bounds, alpha level at .05, and a power familiy function. The spending fuction suggested an alpha level of .0125, .0161, .0203, .0248 at the four different times.

Final Sample. We stopped data collection at the third stage ($N = 1600$) and recruited a total of $N = 1607$ US participants via Prolific.co. The study was advertised at 10 minutes and participants were paid a base payment of £1.00 and could earn a maximum bonus of

£1.80 dependent on their performance in the task. We excluded participants that failed the repeated comprehension check (*n* = 21), were faster than 1/3 of the median time (*n* = 12), did not correctly commit to the oath (e.g., by typing a different text or just writing “Yes” or their ID number; *n* = 13), duplicate IDs (*n* = 1), and participants that were not matched with a partner or where the partner left the game during the task (*n* = 21).

This results in a final sample of 1540 participants (763 men, 748 women, 23 non-binary, 6 prefer not to say) ranging from 18 to 78 years of age (*M* = 41.12, *SD* = 13.27). A total of 1494 indicated a US nationality (*n* = 46 *other*).

Design & Procedure

The study featured a 2 (between: commitment to individual – no partner vs. partner) x 2 (between: commitment to oath – no oath vs. oath) between design. Participants were randomly assigned to the baseline (*n* = 419), partner (*n* = 345), oath (*n* = 424), or partner+oath (*n* = 352) treatment. Participants in the partner and partner+oath treatments were matched with a partner and took the role of Player A or Player B. Participants in the baseline and oath treatments took both roles. As the task featured 10 rounds, participants in the partner and partner+oath provided 10 datapoints each, and participants in the baseline and oath treatments 20 datapoints each (*n* = 23830 total observations). In the partner treatment, *n* = 178 were assigned to role of Player A and *n* = 167 assigned to the role of Player B. In the partner+oath treatment, *n* = 172 were assigned role of Player A and *n* = 180 role of Player B. A total of *n* = 82 dyads consisted of participants that were both allocated to the partner treatment, *n* = 85 dyads consisted of participants where both were allocated to the partner+oath treatment, and *n* = 190 dyads consisted of participants in which both were allocated to different treatments (partner or partner+oath). Of these, *n* = 99 included a dyad with Player A in the partner and Player B in the partner+oath treatment and *n* = 91 a dyad with Player A in the partner+oath treatment and Player B in the partner treatment.

Participants were presented with information about the study and instructions that they will engage in ten rounds of the sequential die-roll game based on Weisel and Shalvi (2015). If they failed one of the three comprehension questions they were presented with the instructions again and able to complete the comprehension items. In each round two dice are rolled and participants can earn points if both reported die rolls match (1 point for double 1, 2 points for double 2, 3 points for double 3 and so on). In the partner and partner+oath treatments, participants were matched with another participant from either the partner or partner+oath treatment. They were randomly assigned to first (Player A) or second (Player B) mover. Player A always reported first, this was sent to Player B, who then reported their die roll. Finally, both players were informed of the outcome and proceeded to the next round. In the baseline and oath treatments, participants took the roles of both Player A and B and reported to die rolls per round. Before the task, participants completed the commitment and IOS item (with regard to their partner in the partner and partner+oath treatments and with regard to another person from the study in the baseline and oath treatments). In the oath and partner+oath treatments participants were then presented with the oath and asked to commit to it by typing it into a text box. They then engaged in a practice trial. After the practice trial each participant performed ten rounds of the sequential die roll task. At the end of the die roll task one round was randomly selected to determine the bonus payoff of the participant or dyad. The main differences between our setup and the task by [3] is that the current task lasted for 10 rounds (instead of 20) and we used a different payoff scheme. Apart from that, the main setup was mostly identical. After the sequential die roll task, participants in the partner and partner+oath treatments were asked to complete the commitment and IOS items once more, were presented with the honesty-humility scale, completed demographic items and were debriefed.

Measures

Sequential Die Roll Task. The sequential die roll task was adapted from previous studies (e.g., [3,29]). Participants were asked to roll a die in private. They could either do this by rolling an actual die themselves or going to an external website that simulates a die roll. They were asked to report their die roll outcome. In the baseline and oath treatments, participants were asked to roll a die twice per round and report the outcomes sequentially. Here, bonus payoffs depended on whether the participant reported the same outcome ($p_i = p_j$). In this case, the associated outcome was equal to the final bonus payoff ($p_i = \pi_i$). If participants reported different outcomes ($p_i \neq p_j$), they did not receive any bonus payoff ($\pi_i = 0$) for this round. This was repeated for ten rounds and at the end one round was randomly chosen for payment. In the partner and partner+oath the setup was the same except that participants reported only one die roll. As Player A they reported first. As Player B they reported second. If participants reported the same outcome ($p_i = p_j$), they received points based on the reported outcome ($p_i = \pi_i$). If participants reported different outcomes ($p_i \neq p_j$), they did not receive any bonus payoff ($\pi_i = 0$) for this round. Both participants in a dyad received the same bonus outcome at the end of the study. Payoffs were based on the selected round and whether a double was reported or not (0 – no double = £0; 1 = £0.3, 2 = £0.6, 3 = £0.9, 4 = £1.2, 5 = £1.5, 6 = £1.8).

IOS. In the partner and partner+oath conditions, participants completed the inclusion-of-the-other-in-the-self scale (IOS), a 7-point scale with Venn diagrams increasing in overlap right before and after the dishonesty task. In the remaining conditions, this was only completed before the experiment asking about their overlap to a random person participating in the experiment.

Perceived Commitment. In the partner and partner+oath conditions, participants completed an item asking about how committed they feel to their partner on a 7-point scale from 0 – not at all committed to 6 – very much committed right before and after the dishonesty task. In the remaining conditions, this was only completed before the experiment asking about their overlap to a random person participating in the experiment.

Honesty-Humility We used the 4 item honesty-humility scale [30] on a 5-point scale from "strongly disagree" to "strongly agree" (α = .59). We added an attention check item to the honesty-humility items stating "This is an attention check. Please select "3".

Demographics We added items assessing participants' gender, age, nationality (US vs. other), and three items on SES, and childhood SES [28].

1.7 Live Interaction Details.

In Studies 4-7, we implemented a live matching and interaction in the *partner* and *partner+oath* treatments using the SMARTRIQS framework [26]. Participants in these treatments were randomly matched with another participant from one of the same treatments, which determined their partner for the rest of the study.[8] The matching procedure could take up to two minutes, during which participants were asked to wait in a "waiting room", and the survey was terminated if no other participant could be matched at the same time. After being successfully matched, participants received instructions that they would engage in a chat for up to 3-minutes (Studies 4-5) or 2-minutes (Study 6) with their partner and that they could exit the chat before the time was up.[9] In Studies 4 and 5, in order to strengthen commitment

[8] Note that participants were not able to tell whether they were in the *partner* or *partner+oath* condition in Studies 4-5 as the only difference between conditions (the oath) was implemented after the partner chat. In Study 6, inspecting chat logs verified that no participant communicated this.

[9] Due to a technical error, chat duration was not recorded. Based on chat logs it is highly likely that the majority of dyads exited the chat before the three or two minutes were up.

to the partner (see e.g., [11]), participants were given the task to come up with a group name during the chat and write it down after the chat. This name was used for subsequent instructions when referring to the shared payoff scheme. If participants timed out (e.g., by leaving the survey or not responding) during the first stage of interaction (i.e., the group chat), their partner was not able to proceed with the study, but the survey was terminated. If participants time out during the second stage of interaction (i.e., the economic game task) a default response was generated by randomly picking one out of the six possible answers and this was used to determine the group's final bonus. The remaining participant was then informed that their partner timed out and that the final response was randomly generated. Of all initiated dyads a small number was terminated at the first or second stage for the final sample (Study 4: 4.37% 16/366; Study 5: 2.5% 6/240; Study 6: 2.14% 7/327). The complete participants of these dyads were still included in the final analyses. In Study 7, if a participant's partner timed out during the experiment the experiment was stopped for the remaining partner. Due to the length of the setup, it did not make sense to give the remaining participants a chance to proceed with the study. In total 4.29% 16/373 dyads timed out at a certain stage. These groups were excluded from the final analyses.

2. Supplementary Note 1 - Sample Size Determination

For Study 1, we based the sample size on the smallest effect size (the effect of social commitment to individuals on honesty, $g = -.22$) as reported in the previous meta-analysis by [1]. In Study 2, we focused on the meta-analytic effect from this meta-analysis for the specific paradigm used (for investment/effort tasks, $g = -.19$). Study 3, focused on the oath effect size obtained in Study 1 and 2. In Study 4 and 5 we used the effect size for the oath treatment from Study 3. And finally for Study 6, we employed the effect size for the oath treatment from Study 4. Power calculations were performed focusing on an ordinal response using the *posamsize* function of the *hmisc* package (version 4.4-1; [2]). Relative frequencies of responses were based on previous studies as detailed in Table 1. Based on these results, suggested sample sizes ranged between 1060 and 7408 participants when considering four treatments in total. Due to resource constraints and the fact that except for Study 6 all studies employed repeated measurements and therefore an increased number of observations, we registered a sample size of 800 in Study 1, 1000 in Study 2, and 500 in Study 3. For Studies 4-6, we registered a sequential analysis approach in order to save resources [24]. Based on the expected effect size, we set a maximum sample size at 1600 and registered four sequential analyses steps (i.e., analyzing the data at 400, 800, 1200, and 1600 participants). As prespecified, recruitment was stopped once the main effects were statistically significant. In order to control for the Type-I-error rate (Lakens, 2014), we adjusted the alpha level at each step based on calculations using the *GroupSeq* package (version 1.4.0; [31]; see Table 1 for more detailed information). In Study 4, we collected the full number of 1600 participants. In Study 5 and 6 we stopped data collections preliminarily, deviating from our registered plans. In Study 5, we stopped during the third wave because we were not able to collect enough participants at the same time, which was essential for the interactive nature of the task leading to a high number of drop-outs. In Study 6, we stopped after the second wave (i.e., 800 participants) although the main effects were not statistically significant. However, we realized that the effects were either tiny or in the opposite direction compared to the previous studies. We think that these changes were sensible and do not alter the overall patterns and findings in any substantial ways. In Study 7, we stopped data collection after 1600 participants, as our main effects were statistically significant according to our preregistered analysis plan.

2.1 Supplementary Note 2 - Sensitivity Analyses.

As we recruited less participants than were suggested by our sample size calculations for all studies, we conducted sensitivity power analyses, testing what minimum effect we could obtain with 90% or 95% power given the specific sample size and alpha level (.05). We performed these analyses on a linear multilevel model for Studies 1-5 and 7 using the *simr* and *lme4* packages [32,33], as sensitivity analyses for multilevel ordinal logistic regression models are not trivial. Our estimates might therefore not fully represent the sensitivity of our actual model, but they provide a specific idea of what effects sizes we could detect with the current model and power. For study 6, we conducted a sensitivity analysis based on a *t*-test using the *pwr* package [34]. For all sensitivity analyses we focused on the effect of the honesty oath (compared to the baseline). An overview is presented in Supplementary Table 1.

Supplementary Table 1. Overview of minimal detectable effect size of the seven different studies given 90% or 95% power.

Study	N	n observations	Observed effect (OR)	Minimal effect size with 90% power			Minimal effect size with 95% power		
				d	OR	logOR	d	OR	logOR
1	770	3080	.80	-.195	.70	-.35	-.215	.68	-.39
2	1494	5976	.84	-.12	.80	-.22	-.1348	.78	-.25
3	484	4840	.74	-.19	.71	-.35	-.21	.68	-.38
4	1541	3896	.72	-.166	.74	-.30	-.18	.72	-.33
5	982	2491	.76	-.191	.71	-.35	-.2047	.69	-.37
6	755	755	1.16	-.30	.58	-.54	-.34	.54	-.62
7[a]	1193	20360	.86	-.092	.85	-.17	-.103	.83	-.187

[a]Values for die roll reports (including only Player A for the partner and partner+oath treatments). For double reports (including only Player B for the partner and partner+oath treatments) results are: N = 1190, *n* = 11900, Observed effect (OR) = .72, 90% power: *d* = -.20, 95% power: *d* = -.24.

3. Supplementary Note 3 - Designs

Study 1 featured a 4 (social commitment - between) x 2 (target - within: self, other) mixed design. Participants were randomly assigned to one of the four social commitment treatments and then completed the same economic game four times for both the self and another randomly selected participant from the same study whom they did not interact with. Studies 2 and 3 included a 4 (social commitment – between) x 2 (task – between: mind shape, die roll) between design. While Study 2 included four rounds of the task, participants completed ten rounds in Study 3, as we were interested in exploring the effect over time. Studies 4 and 5 employed a 4 (social commitment – between) x 2 (length of task – between: four trial lottery, one-shot) between subject design. Study 6 used a 4 (social commitment – between) x 2 (type of payoff – between: shared payoff, double) nested design. The type of payoff was nested in the partner and partner+oath treatments.

4. Supplementary Note 4 - Procedures

Participants always received information about the study and their rights and provided informed consent. Afterwards, they were randomly allocated to one of the four social commitment treatments (see Table 3 for cell sizes). In Study 1, participants in the *oath* and *partner+oath* treatments were then presented with the oath manipulation and then participants in all treatments completed the Artistic Preference Task (ART [4]). In all studies,

participants then received instructions about the specific economic game task including an item probing for understanding of the task.[10] In Studies 4 and 5 in the *partner* and *partner+oath* treatments, participants were afterwards matched with another partner and engaged in a chat for up to 3-minutes before the main task with the objective of deciding on a group name for the dyad – as task that has been successfully implemented in previous studies to induce commitment (e.g., [11]). In all except Study 1, participants then completed IOS and commitment items to assess their commitments towards the matched partner. In Study 2, these measures were only included in the *partner* and *partner+oath* treatments. In Studies 2-6, participants in the *oath* and *partner+oath* treatments were at this stage presented with the oath manipulation. In all studies, participants then completed the main economic game task. The type of task, number of rounds, and how the final bonus payment was calculated differed across studies as summarized earlier and in Table 2. In Study 6, participants completed the die roll paradigm [15] by first rolling a die in private and then completing a chat with up to 2-minutes with their matched partner in the *partner* and *partner+oath* conditions to discuss their final report, as the individual report influenced the group payoff. In all studies participants then completed items to assess commitment to the partner, an item on felt responsibility and a short questionnaire on trait honesty-humility.[11] Participants were then thanked and debriefed.

5. Supplementary Note 5 - Additional Measures

Felt Commitment. We measured commitment to the other partner (in the *partner* and *parter+oath* treatments) or someone from the experiment that participants were not matched with (in the *baseline* and *oath* treatments), by using the inclusion-of-the-other-in-the-self scale (IOS; [19]) and an item asking how committed participants felt to their partner on a 7-point scale from 0 – not at all committed to 6 – very much committed, right before and after the dishonesty task. The IOS item depicts seven Venn-like diagrams with two circles representing the self and the other increasing in overlap. In Study 1, the item focused on the *other* target across all treatments and was only presented after the game task. In Study 2, these items were only used in the *partner* and *partner+oath* treatments.
Honesty-Humility. We employed the 10-item Honesty-Humility scale (see Table 2 for individual reliabilities; [20]) on a 5-point scale from "strongly disagree" to "strongly agree". We also added one attention check item among the honesty-humility items. Participants failing this attention check were screened out (see Table 1).[12] In Study 7, participants completed the 4-item Honesty-Humility scale [30].
Felt Responsibility. One possible mechanism could be that felt moral responsibilities differs between cheating for others and cheating after taking an oath. We assessed responsibility by asking participants "How much responsibility did you feel for your actions during the [xx] task" on a 5-point scale from "none at all" to "a great deal".[13] This item was not included in Study 7 based on the fact that it did not show any consistent effects across the other studies.

---

[10]In case of choosing the wrong answer, participants were shown the instructions once more and had another opportunity to respond to the probe item.

[11]In Study 1, participants also completed a questionnaire on trait self-control, but this measure was dropped in the later studies as it did not show any relationship with dishonesty.

[12]In Study 1, participants also completed the 12-item self-control scale (〈 = .87; Tangney et al., 2004) on a 5-point scale from "not at all like me" to "very much like me" and a measure to rate the similarity of the shapes employed in the mind shape task. Across all studies participants also indicated their felt responsibility for their actions. Detailed descriptions are provided in the Supplementary Material Section 5.

[13]Due to an error this item was only assessed in the *partner* and *partner+oath* treatments in Study 2.

Self-Control. In Study 1, participants completed the 12-item self-control scale [21] on a 5-point scale from "not at all like me" to "very much like me".

Spatial Arrangement Similarity Rating (Q-SpAM). We adapted the Q-SpAM paradigm [5], a spatial arrangement task to study similarity ratings among mind shapes in Study 1. Participants were asked to arrange the eleven shapes employed in the experiment by dragging them across their entire screen, arrange more similar shapes closer to each other and more dissimilar shapes more distant from each other.

6. Supplementary Note 6 – Assumptions for main Analyses

In general, there are several assumptions when running an ordinal logistic regression, with the. The first assumption is that the dependent variable is measured at an ordinal level. The second assumption is that the independent variables are either categorical, ordinal, or continuous. The third assumption is that no multi-collinearity exists among independent variables. And the fourth assumption is the assumption of proportional odds, that the independent variable has a similar effect on the different levels of the dependent variable [35]. The first three assumptions were always met. We tested the fourth assumption using the Brant test employing the brant package in R. As the Brant test hasn't been implemented for multilevel ordinal logistic models in R, we needed to refit the main models ignoring the multilevel structure with the *polr* command in the MASS package. We only included treatment as the independent variable. In all studies the tests were not statistically significant, meaning that the parallel regression or proportional odds assumption held.

Supplementary Table 2.

Test for	X2	df	*p*
Study 1			
Omnibus	17.72	12	.12
Treatment2	7.32	4	.12
Treatment3	4.16	4	.39
Treatment4	5.04	4	.28
Study 2			
Omnibus	8.08	12	.78
Treatment2	1.57	4	.81
Treatment3	2	4	.74
Treatment4	3.76	4	.44
Study 3			
Omnibus	12.44	12	.41
Treatment2	1.83	4	.77
Treatment3	6.12	4	.19
Treatment4	1.33	4	.86
Study 4			

Omnibus	6.28	12	.90
Treatment2	4.59	4	.33
Treatment3	2.68	4	.61
Treatment4	.77	4	.94
Study 5			
Omnibus	10.51	12	.57
Treatment2	6.83	4	.15
Treatment3	5.39	4	.25
Treatment4	2.87	4	.58
Study 6			
Omnibus	9.98	12	.62
Treatment2	3.09	4	.54
Treatment3	4.83	4	.31
Treatment4	3.01	4	.56
Study 7			
Omnibus	14.56	12	.27
Treatment2	3.27	4	.51
Treatment3	6.17	4	.19
Treatment4	5.98	4	.20

Note. Treatment2 = Partner, Treatment3 = Oath, Treatment4 = Partner+Oath.

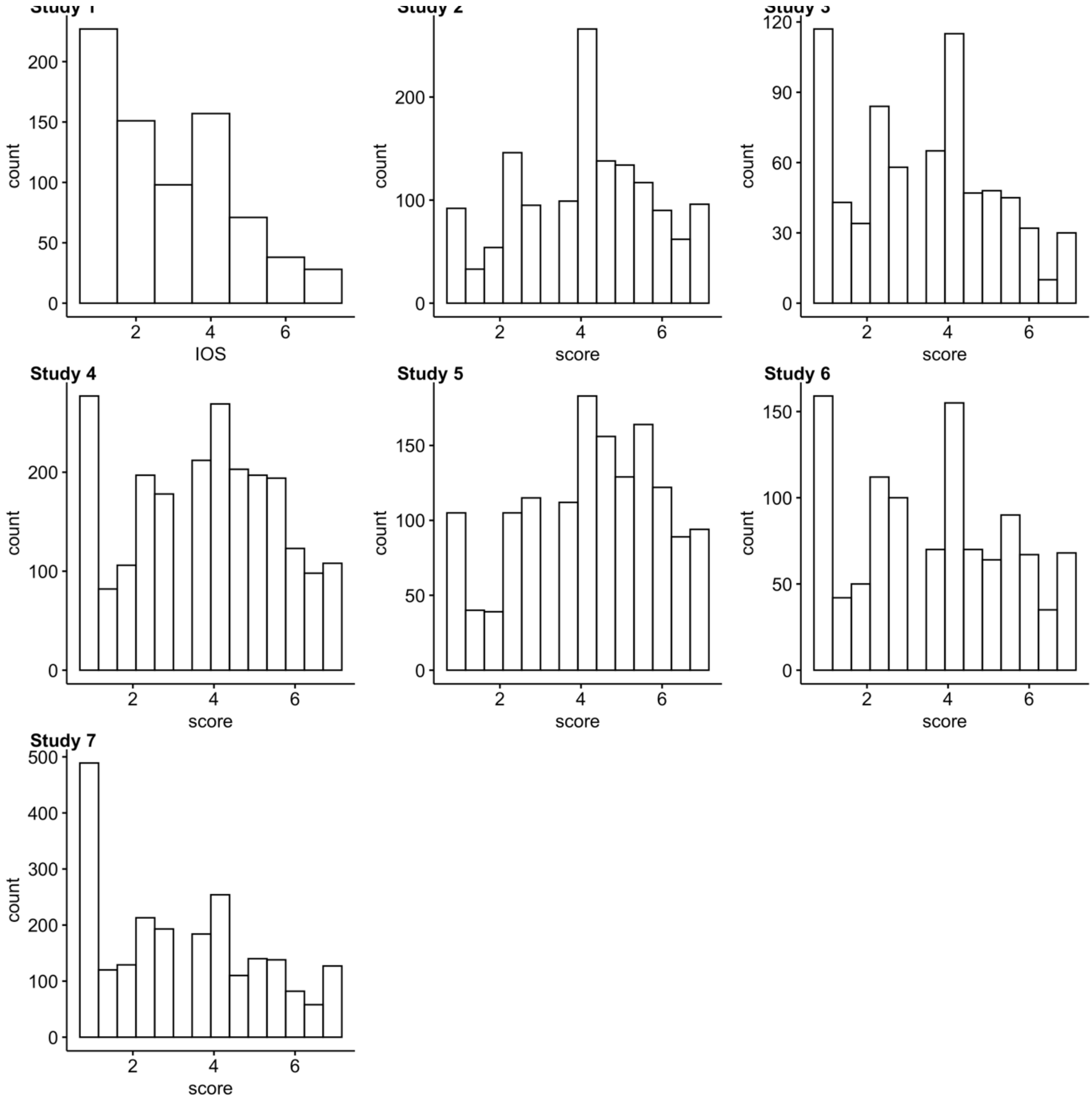

Supplementary Figure 2. Overview of distributions of commitment to the partner rating (IOS item in Study 1) across the seven studies.

7. Supplementary Note 7 - Determination of SESOI

Based on recent meta-analyses [1,36–38] common effect sizes in dishonesty literature are around $d = 0.22$ and 0.88 and *small* effect sizes in social psychology are around $d = 0.15$ [39]. Based on these findings, we set the SESOI at $d = .15$, which translates to an OR of 1.313.

8. Manipulation Check – Felt Commitment

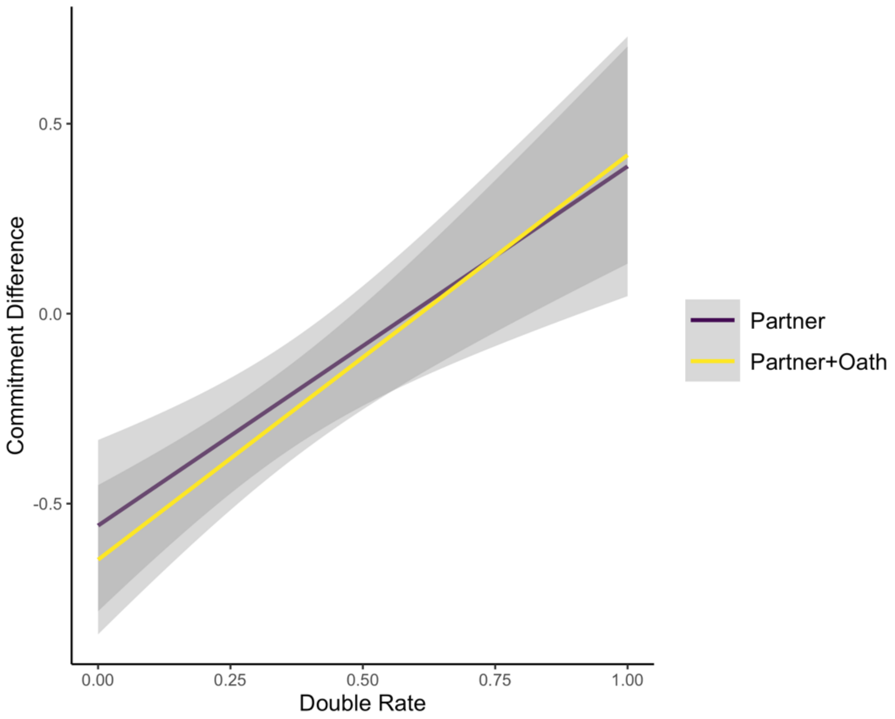

Supplementary Figure 3. Overview of relationship between commitment difference (commitment_post – commitment_pre) and double rate (i.e., how many doubles were reported across the ten rounds) for the partner and partner+oath treatments in Study 7.

For the difference between commitment before the task and after the task, we observed a significant moderation by double rate in Study 7, $F(1,694) = 39.88$, $p < .001$. We observed that commitment increased after the task for higher frequency of doubles in the game ($r = .23$ [.16, .30]).

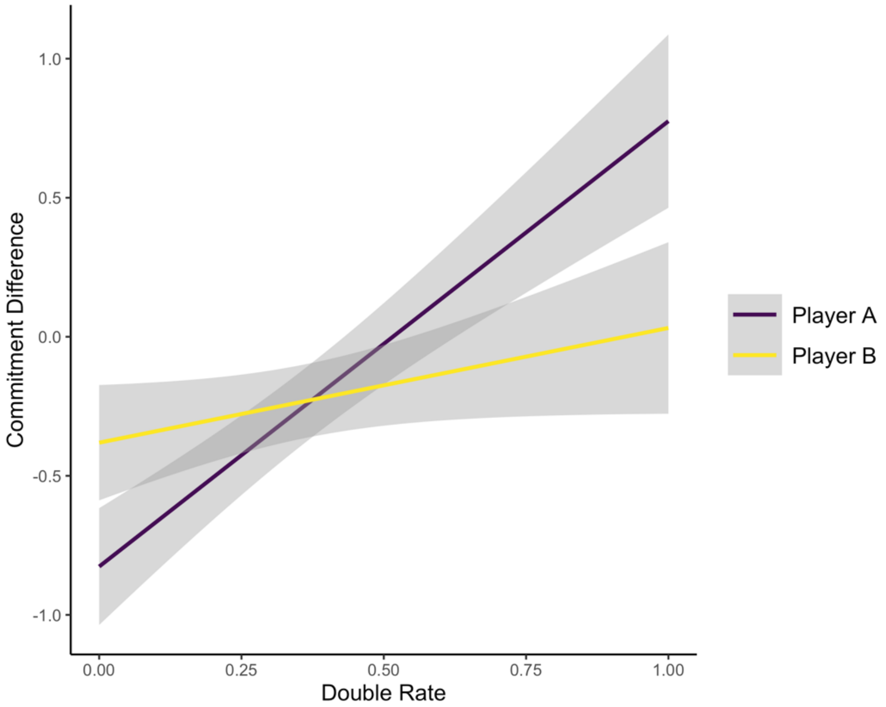

Supplementary Figure 4. Overview of relationship between commitment difference (commitment_post – commitment_pre) and double rate (i.e., how many doubles were reported across the ten rounds) for the role of Player A and Player B in Study 7.

We further explored this relationship with regard to whether participants had the role of Player A or Player B. We observed a stronger correlation for the relationship between commitment difference and double rate for Player A (r = .36) compared to Player B (r = .10) see Figure 4.

9. Equivalence Tests.

a) Equivalence of Partner Effects

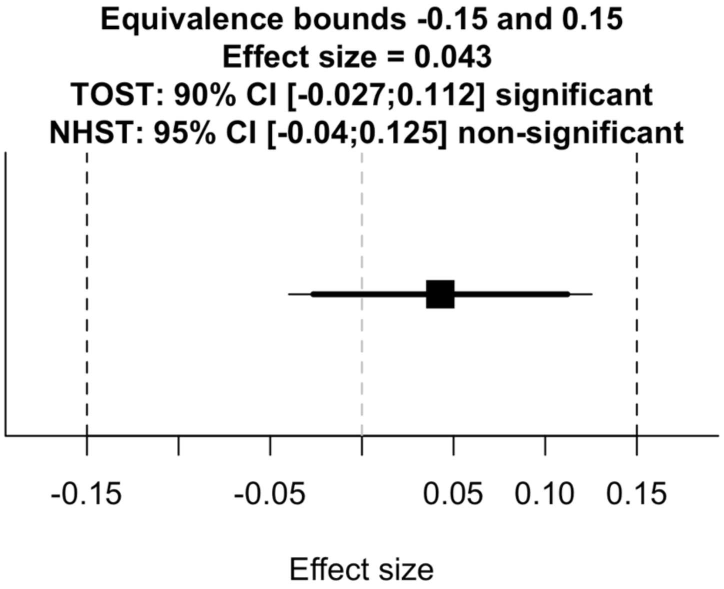

b) Equivalence of Oath Effects

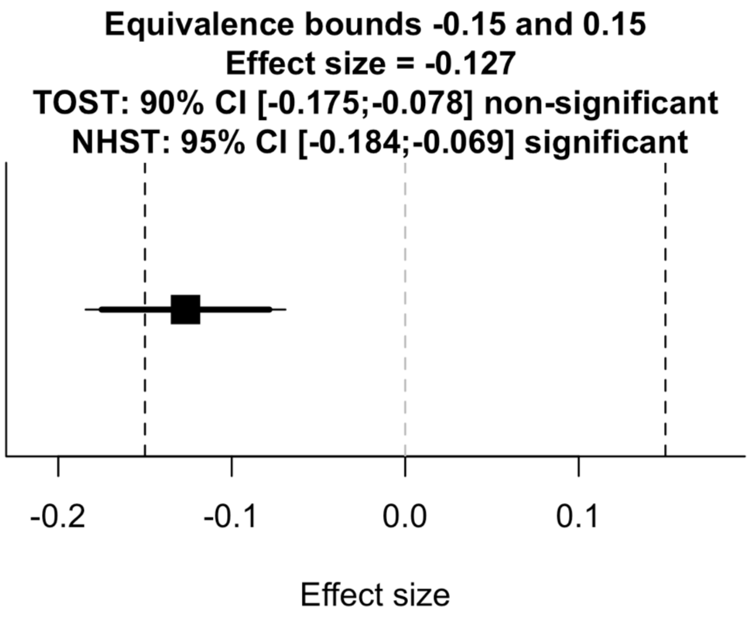

c) Equivalence Test of Partner+Oath Effect

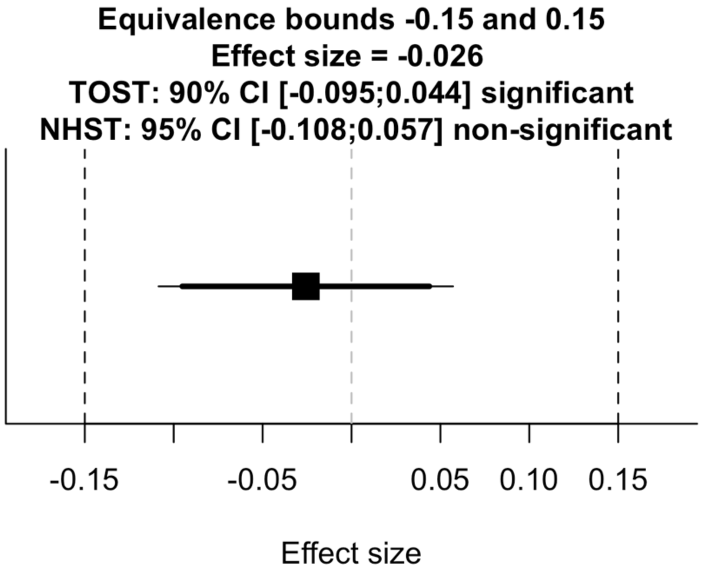

Supplementary Figure 5. Overview of equivalence test for the partner (a), oath (b) and parter+oath (c) effects.

10. Supplementary Note 8 – Registered Analyses Study 1

We registered different analyses in Study 1 mainly due to the slightly different focus when conducting the study. Here, we present these originally preregistered analyses.

Note, that we registered a multilevel model for the manipulation check originally, but this was a typo and the model misspecified, as participants only completed one IOS rating.

The original hypotheses were:

Effects across targets:

H1. Social commitment to other individuals (compared to the baseline) increases dishonest behavior.
H2. Social commitment to an oath (compared to the baseline) decreases dishonest behavior.
H3. Combining social commitment to individuals and to an oath (compared to the baseline) does neither increase nor decrease dishonest behavior (
null effect
).
Effects for specific targets:
H4: Prosocial cheating (i.e., cheating for the partner) increases in the minimal group paradigm
condition compared to the baseline condition.
H5: Prosocial cheating (i.e., cheating for the partner) decreases in the minimal group and oath treatment compared to the minimal group condition.
H6: Signing an oath (both in the oath and MG & oath treatments) will reduce cheating for the self in comparison to the baseline.

Analyses:
H1-H3 (Supplementary Table S10.0.1): Multilevel ordinal logistic model with the mind shape score (i.e., amount of money
earned per trial) as DV and commitment treatment as IV. We will focus on the main effect and follow up with planned comparisons, comparing each experimental treatment to the baseline.
H4-H7 (Supplementary Table S10.0.2): Multilevel ordinal logistic model with mind game score as DV and commitment,
target conditions, and their interaction as IVs. We will focus on the interaction effect and follow up with planned comparisons. We compare ingroup target MGP treatment with baseline (H4), ingroup target MGP and oath with MGP (H5), self target oath and MGP and oath with baseline (H6)

Supplementary Table 3. Overview of the originally registrered model (including both self and other target ratings) in Study 1.

	**Dependent variable**		
Predictors	*Odds Ratios*	*CI*	*p*
1|2	0.09	.07 - .10	**<0.001**
2|3	0.24	.21 - .29	**<0.001**
3|4	0.52	.45 - .61	**<0.001**

4|5	1.15	.99 – 1.34	**0.037**
5|6	3.09	2.65 – 3.61	**<0.001**
Treatment [Partner]	1.09	.88 – 1.34	0.435
Treatment [Oath]	.85	.68 – 1.05	0.123
Treatment [Partner+Oath]	1.06	.86 – 1.31	0.575
Observations	6160		

Treatment uses the baseline as the comparison.

Supplementary Table 4. Overview of the originally registrered model (including both self and other target ratings) in Study 1 with treatment and target.

Dependent variable

Predictors	*Odds Ratios*	*CI*	*p*
1|2	0.07	.06 - .09	**<0.001**
2|3	0.21	.17 - .25	**<0.001**
3|4	.44	.37 - .53	**<0.001**
4|5	.98	.82 – 1.67	**0.402**
5|6	2.65	2.22 – 3.17	**<0.001**

Treatment [Partner]	1.11	.86 – 1.42	0.418
Treatment [Oath]	.90	.70 – 1.15	0.385
Treatment [Partner+Oath]	1.02	.80 – 1.31	0.855
Target [Other]	.72	.60 - .86	**<.001**
Treatment[Partner]:Target[Other]	.97	.75 – 1.24	.794
Treatment[Oath]:Target[Other]	.89	.69 – 1.15	.367
Treatment[Partner+Oath]:Target[Other]	1.08	.84 – 1.39	.560
Observations	6160		

Treatment uses the baseline as the comparison.

10. Sensitivity Analysis Study 1 & 2

10.1 Study 1.

Supplementary Table 5. Overview of main model using the preregistered filter in Study 1.

	**Dependent variable**		
Predictors	*Odds Ratios*	*CI*	*p*
1|2	0.11	0.09 – 0.13	**<0.001**
2|3	0.31	0.26 – 0.37	**<0.001**

3|4	0.62	0.52 – 0.74	**<0.001**
4|5	1.34	1.13 – 1.59	**0.001**
5|6	3.55	2.98 – 4.25	**<0.001**
Treatment [Partner]	1.04	0.83 – 1.32	0.719
Treatment [Oath]	0.85	0.67 – 1.07	0.168
Treatment [Partner+Oath]	1.14	0.90 – 1.44	0.282
N $_{as.factor}$	804		
Observations	3216		

Note. Analyses only on mind shape ratings for the other target (not self). Treatment uses the baseline as the comparison.

Supplementary Table 6. Overview of main model using the self-target in Study 1.

	**Dependent variable**		
Predictors	*Odds Ratios*	*CI*	*p*
1|2	0.06	0.05 – 0.08	**<0.001**
2|3	0.19	0.16 – 0.24	**<0.001**
3|4	0.46	0.38 – 0.55	**<0.001**

4|5	1.01	.84 – 1.22	
5|6	2.74	2.27 – 3.30	**<0.001**
Treatment [Partner]	1.12	0.87 – 1.44	0.389
Treatment [Oath]	0.91	0.70 – 1.17	0.456
Treatment [Partner+Oath]	1.03	0.80 – 1.33	0.802

Note. Analyses only on mind shape ratings for the self. Treatment uses the baseline as the comparison.

10.2 Study 2.

Supplementary Table 7. Overview of main model using the preregistered filter in Study 2.

	**Dependent variable**		
Predictors	*Odds Ratios*	*CI*	*p*
1|2	0.11	0.09 – 0.12	**<0.001**
2|3	0.34	0.31 – 0.37	**<0.001**
3|4	0.73	0.67 – 0.81	**<0.001**
4|5	1.67	1.52 – 1.84	**<0.001**
5|6	4.38	3.94 – 4.86	**<0.001**
Treatment [Partner]	0.98	0.86 – 1.12	0.785

Treatment [Oath]	0.83	0.73 – 0.95	**0.007**
Treatment [Partner+Oath]	0.93	0.81 – 1.07	0.311
N as.factor	1518		
Observations	6072		

Note. Treatment uses the baseline as the comparison.

10.3 Study 7.

Here, we provide analyses focusing on die roll report as the outcome variable (instead of reported double).

Supplementary Table 8. Overview of model including task report (die roll report) as the outcome variable focusing on all participants. Note, that a higher die roll report does not automatically indicate cheating for Player B in the group treatments as their objective is to match Player A's report. Therefore, this model should be interpreted with caution and the model including Player A reports only is more informative.

	**Rating**		
Predictors	*Odds Ratios*	*CI*	*p*
1|2	0.12	0.11 – 0.14	**<0.001**
2|3	0.31	0.29 – 0.34	**<0.001**
3|4	0.66	0.60 – 0.72	**<0.001**
4|5	1.34	1.23 – 1.46	**<0.001**
5|6	3.45	3.16 – 3.78	**<0.001**
Partner	0.98	0.85 – 1.12	0.731
Oath	0.82	0.72 – 0.92	**0.001**
Partner+Oath	0.92	0.80 – 1.05	0.199
**Random Effects**			
σ^2	3.29		
$\tau_{00\ ID\_i}$	0.66		
ICC	0.17		
N_{ID_i}	1540		

Observations	23830
Marginal R^2 / Conditional R^2	0.002 / 0.168

Supplementary Table 9. Overview of model including task report (die roll report) as the outcome variable focusing on Player A for the *partner* and *partner+oath* treatments.

	Rating		
Predictors	*Odds Ratios*	*CI*	*p*
1|2	0.12	0.11 – 0.14	**<0.001**
2|3	0.31	0.28 – 0.34	**<0.001**
3|4	0.65	0.59 – 0.72	**<0.001**
4|5	1.33	1.21 – 1.47	**<0.001**
5|6	3.42	3.11 – 3.77	**<0.001**
Partner	1.15	0.96 – 1.37	0.134
Oath	0.82	0.72 – 0.93	**0.002**
Partner+Oath	0.99	0.83 – 1.19	0.916
**Random Effects**			
σ^2	3.29		
$\tau_{00\ ID\_i}$	0.77		
ICC	0.19		
N $_{ID\_i}$	1193		
Observations	20360		
Marginal R^2 / Conditional R^2	0.003 / 0.193		

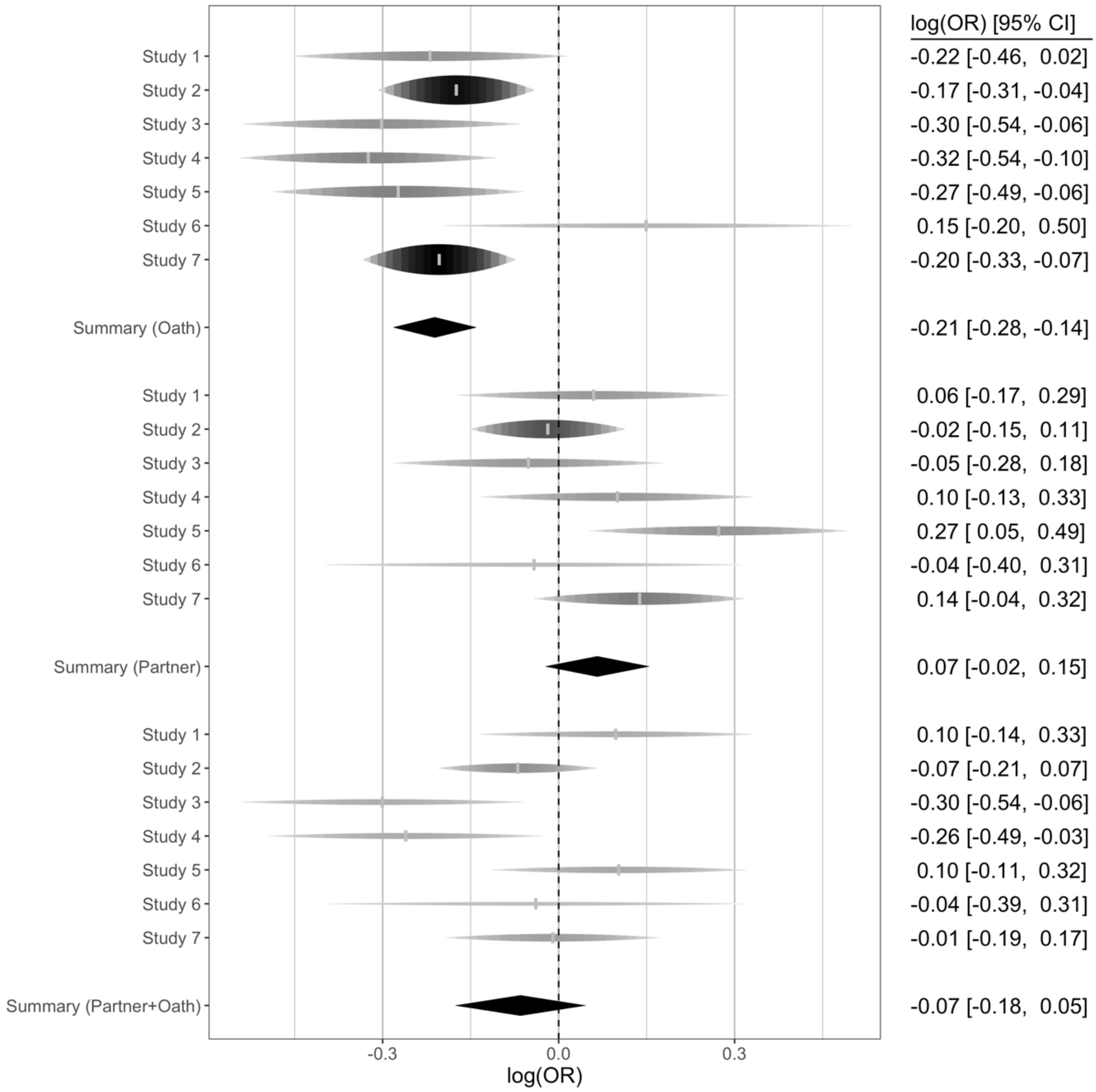

Supplementary Figure 6. Overview of effect sizes using reported die roll as the outcome in Study 7 (for Player A only in the partner and partner+oath treatment). Partner: OR = 1.07 [.98, 1.16]; Oath: OR = .81 [.75, .88]; Partner+Oath: OR = .94 [.86, 1.02].

11. Moderation Analysis

11.1 Study 1.

Supplementary Table 10. Overview of main model including type of target (self: -.5, vs. other: .5) in Study 1.

	Dependent variable		
Predictors	*Odds Ratios*	*CI*	*p*

1|2	0.07	0.06 – 0.09	**<0.001**
2|3	0.21	0.17 – 0.25	**<0.001**
3|4	0.44	0.37 – 0.53	**<0.001**
4|5	0.98	0.82 – 1.17	0.804
5|6	2.65	2.22 – 3.17	**<0.001**
Treatment [Partner]	1.11	0.86 – 1.42	0.418
Treatment [Oath]	0.90	0.70 – 1.15	0.385
Treatment [Partner + Oath]	1.02	0.80 – 1.31	0.855
Target [Other]	0.72	0.60 – 0.86	**<0.001**
Treatment [Partner] * Target [Other]	0.97	0.75 – 1.24	0.794
Treatment [Oath] * Target [Other]	0.89	0.69 – 1.15	0.367
Treatment [Partner+Oath] * Target [Other]	1.08	0.84 – 1.39	0.560
$N_{as.factor}$	770		
Observations	6160		

Note. Treatment uses the baseline as comparison, target uses self as the comparison.

Supplementary Table 11. Overview of the main model including type of target (self: -.5, vs. other: .5) and honesty-humility (centered) in Study 1.

	**Dependent variable**		
Predictors	*Odds Ratios*	*CI*	*p*
1|2	0.07	0.06 – 0.09	**<0.001**
2|3	0.21	0.17 – 0.25	**<0.001**
3|4	0.44	0.37 – 0.53	**<0.001**
4|5	0.97	0.82 – 1.16	0.756
5|6	2.64	2.21 – 3.15	**<0.001**
Treatment [Partner]	1.10	0.86 – 1.41	0.433
Treatment [Oath]	0.88	0.69 – 1.13	0.327
Treatment [Partner+Oath]	1.03	0.80 – 1.32	0.811
Target [Other]	0.72	0.60 – 0.86	**<0.001**
Honesty-Humility (HH)	0.85	0.64 – 1.12	0.250
Treatment [Partner] * Target [Other]	0.96	0.75 – 1.24	0.777

Treatment [Oath] * Target [Other]	0.89	0.69 – 1.15	0.377
Treatment [Partner+Oath] * Target [Other]	1.07	0.83 – 1.38	0.591
Treatment [Partner] * HH	0.70	0.47 – 1.07	0.097
Treatment [Oath] * HH	1.04	0.70 – 1.54	0.846
Treatment [Partner+Oath] * HH	0.89	0.59 – 1.33	0.554
Target [Other] * HH	1.00	0.75 – 1.32	0.988
(Treatment [Partner] * Target [Other]) * HH	1.32	0.86 – 2.01	0.205
(Treatment [Oath] * Target [Other]) * HH	1.02	0.69 – 1.53	0.906
(Treatment [Partner+Oath] * Target [Other]) * HH	1.12	0.74 – 1.70	0.587
$N_{as.factor}$	770		
Observations	6160		

Note. Treatment uses the baseline as comparison, target uses self as the comparison.

11.2 Study 2.

Supplementary Table 12. Overview of main model including type of task (mind shape: 1, vs. die roll: 2) in Study 2.

	Dependent variable		
Predictors	*Odds Ratios*	*CI*	*p*
1|2	0.11	0.10 – 0.13	**<0.001**
2|3	0.36	0.32 – 0.41	**<0.001**
3|4	0.79	0.69 – 0.89	**<0.001**
4|5	1.77	1.56 – 2.02	**<0.001**
5|6	4.66	4.07 – 5.35	**<0.001**
Treatment [Partner]	1.03	0.86 – 1.24	0.761
Treatment [Oath]	0.83	0.69 – 1.00	0.052
Treatment [Partner+Oath]	0.94	0.77 – 1.13	0.491
Task [Die Roll]	1.15	0.96 – 1.38	0.131
Treatment [Partner] * Task [DR]	0.90	0.69 – 1.18	0.454
Treatment [Oath] * Task [DR]	1.02	0.78 – 1.33	0.907
Treatment [Partner+Oath] * Task [DR]	0.99	0.75 – 1.30	0.929
$N_{as.factor}$	1494		
Observations	5976		

Note. Treatment uses baseline as comparison and task uses mind-shape task. DR = die roll.

Supplementary Table 13. Overview of main model including type of task (mind shape: 1, vs. die roll: 2) and honesty-humility (centered) in Study 2.

	Dependent variable		
Predictors	*Odds Ratios*	*CI*	*p*
1|2	0.11	0.10 – 0.13	**<0.001**
2|3	0.36	0.32 – 0.41	**<0.001**
3|4	0.79	0.69 – 0.89	**<0.001**
4|5	1.77	1.56 – 2.02	**<0.001**
5|6	4.66	4.07 – 5.34	**<0.001**
Treatment [Partner]	1.03	0.86 – 1.24	0.737
Treatment [Oath]	0.81	0.67 – 0.97	**0.025**
Treatment [Partner+Oath]	0.94	0.77 – 1.13	0.491
Task [Die Roll]	1.15	0.95 – 1.38	0.147
Honesty-Humility (HH)	0.93	0.76 – 1.13	0.462
Treatment [Partner] * Task [DR]	0.90	0.69 – 1.17	0.429
Treatment [Oath] * Task [DR]	1.05	0.80 – 1.38	0.702
Treatment [Partner+Oath] * Task [DR]	1.00	0.76 – 1.31	0.983

Treatment [Partner] * HH	0.96	0.71 – 1.31	0.813
Treatment [Oath] * HH	1.33	0.96 – 1.82	0.083
Treatment [Partner+Oath] * HH	1.05	0.77 – 1.44	0.753
Task [DR] * HH	1.01	0.76 – 1.36	0.921
(Treatment [Partner] * Task [DR]) * HH	0.98	0.63 – 1.53	0.943
(Treatment [Oath] * Task [DR]) * HH	0.80	0.51 – 1.25	0.322
(Treatment [Partner+Oath] * Task [DR]) * HH	1.20	0.76 – 1.90	0.442
$N_{as.factor}$	1494		
Observations	5976		

Note. Treatment uses baseline as comparison and task uses mind-shape task. DR = die roll.

Supplementary Table 14. Overview of the main model including honesty-humility (centered) in Study 2.

	**Dependent variable**		
Predictors	*Odds Ratios*	*CI*	*p*
1|2	0.11	0.10 – 0.12	**<0.001**
2|3	0.34	0.31 – 0.38	**<0.001**

3|4	0.74	0.67 – 0.81	**<0.001**
4|5	1.66	1.51 – 1.83	**<0.001**
5|6	4.38	3.94 – 4.86	**<0.001**
Treatment [Partner]	0.98	0.86 – 1.12	0.792
Treatment [Oath]	0.84	0.73 – 0.96	**0.011**
Treatment [Partner+Oath]	0.94	0.82 – 1.07	0.344
HH	0.93	0.80 – 1.07	0.313
Treatment [Partner] * HH	0.96	0.77 – 1.20	0.726
Treatment [Oath] * HH	1.15	0.92 – 1.44	0.211
Treatment [Partner+Oath] * HH	1.15	0.91 – 1.45	0.234
$N_{as.factor}$	1494		
Observations	5976		

11.3 Study 3.

Supplementary Table 15. Overview of main model including type of task (mind shape: 1, vs. die roll: 2) in Study 3.

	**Dependent variable**		
Predictors	*Odds Ratios*	*CI*	*p*

1|2	0.05	0.03 – 0.08	**<0.001**
2|3	0.13	0.08 – 0.22	**<0.001**
3|4	0.29	0.17 – 0.49	**<0.001**
4|5	0.64	0.37 – 1.08	0.095
5|6	1.64	0.96 – 2.79	0.069
Treatment [Partner]	0.71	0.35 – 1.44	0.339
Treatment [Oath]	0.61	0.28 – 1.33	0.214
Treatment [Partner+Oath]	0.60	0.28 – 1.26	0.178
Task [Die Roll]	0.70	0.50 – 0.97	**0.033**
Treatment [Partner] * Task [DR]	1.20	0.76 – 1.90	0.441
Treatment [Oath] * Task [DR]	1.14	0.71 – 1.82	0.600
Treatment [Partner+Oath] * Task [DR]	1.14	0.70 – 1.84	0.603
$N_{as.factor}$	484		
Observations	4840		

Supplementary Table 16. Overview of the main model including type of task (mind shape: 1, vs. die roll: 2) and honesty-humility (centered) in Study 3.

	**Dependent variable**		
Predictors	*Odds Ratios*	*CI*	*p*
1|2	0.04	0.03 – 0.07	**<0.001**
2|3	0.12	0.07 – 0.20	**<0.001**
3|4	0.27	0.16 – 0.45	**<0.001**
4|5	0.59	0.35 – 0.99	**0.045**
5|6	1.52	0.91 – 2.54	0.112
Treatment [Partner]	0.72	0.36 – 1.44	0.354
Treatment [Oath]	0.65	0.31 – 1.37	0.255
Treatment [Partner+Oath]	0.56	0.27 – 1.15	0.114
Task [Die Roll]	0.67	0.48 – 0.92	**0.012**
Honesty-Humility (HH)	1.00	0.45 – 2.21	0.997
Treatment [Partner] * Task [DR]	1.14	0.73 – 1.78	0.570
Treatment [Oath] * Task [DR]	1.12	0.70 – 1.77	0.640
Treatment [Partner+Oath] * Task [DR]	1.19	0.75 – 1.89	0.471

Treatment [Partner] * HH	2.05	0.69 – 6.06	0.193
Treatment [Oath] * HH	0.24	0.07 – 0.81	**0.022**
Treatment [Partner+Oath] * HH	0.79	0.26 – 2.38	0.675
Task [DR] * HH	0.73	0.45 – 1.21	0.224
(Treatment [Partner] * Task [DR]) * HH	0.64	0.32 – 1.31	0.222
(Treatment [Oath] * Task [DR]) * HH	3.05	1.41 – 6.58	**0.005**
(Treatment [Partner+Oath] * Task [DR]) * HH	1.45	0.71 – 2.95	0.304
$N_{as.factor}$	484		
Observations	4840		

Supplementary Table 17. Overview of the main model including honesty-humility (centered) in Study 3.

	**Dependent variable**		
Predictors	*Odds Ratios*	*CI*	*p*
1|2	0.08	0.07 – 0.10	**<0.001**
2|3	0.22	0.19 – 0.27	**<0.001**

3|4	0.49	0.41 – 0.58	**<0.001**
4|5	1.09	0.92 – 1.28	0.330
5|6	2.80	2.36 – 3.31	**<0.001**
Treatment [Partner]	0.92	0.73 – 1.15	0.459
Treatment [Oath]	0.74	0.58 – 0.93	**0.011**
Treatment [Partner+Oath]	0.73	0.58 – 0.93	**0.010**
HH	0.65	0.50 – 0.84	**0.001**
Treatment [Partner] * HH	1.14	0.80 – 1.63	0.476
Treatment [Oath] * HH	1.27	0.86 – 1.88	0.234
Treatment [Partner+Oath] * HH	1.35	0.94 – 1.94	0.110
$N_{\text{as.factor}}$	484		
Observations	4840		

11.4 Study 4.

Supplementary Table 18. Overview of main model including type of rounds (four rounds: 1, one-shot: 2) in Study 4.

	**Dependent variable**		
Predictors	*Odds Ratios*	*CI*	*p*

1|2	0.06	0.04 – 0.10	**<0.001**
2|3	0.17	0.11 – 0.28	**<0.001**
3|4	0.40	0.25 – 0.64	**<0.001**
4|5	0.95	0.59 – 1.52	0.829
5|6	2.85	1.78 – 4.56	**<0.001**
Treatment [Partner]	1.19	0.59 – 2.41	0.626
Treatment [Oath]	0.71	0.37 – 1.39	0.320
Treatment [Partner+Oath]	0.64	0.32 – 1.29	0.214
Type [One-Shot]	1.28	0.91 – 1.81	0.154
Treatment [Partner] * Type	0.94	0.57 – 1.55	0.795
Treatment [Oath] * Type	1.02	0.62 – 1.66	0.951
Treatment [Partner+Oath] * Type	1.16	0.69 – 1.95	0.576
$N_{as.factor}$	1541		
Observations	3896		

Note. Comparison for treatment is baseline, comparison for type is four trials type.

Supplementary Table 19. Overview of the main model including treatment, type (1 = four rounds, 2 = one-shot), and honesty-humility (centered) and its interactions in Study 4.

	Dependent variable		
Predictors	*Odds Ratios*	*CI*	*p*
1|2	0.06	0.04 – 0.10	**<0.001**
2|3	0.18	0.11 – 0.28	**<0.001**
3|4	0.41	0.26 – 0.65	**<0.001**
4|5	0.96	0.61 – 1.53	0.877
5|6	2.90	1.82 – 4.62	**<0.001**
Treatment [Partner]	1.31	0.65 – 2.64	0.449
Treatment [Oath]	0.68	0.35 – 1.32	0.260
Treatment [Partner+Oath]	0.63	0.31 – 1.26	0.189
Type [One-Shot]	1.29	0.92 – 1.82	0.139
Honesty-Humility (HH)	0.67	0.32 – 1.44	0.310
Treatment [Partner] * Type [OS]	0.89	0.54 – 1.47	0.657
Treatment [Oath] * Type [OS]	1.06	0.65 – 1.73	0.825

Treatment [Partner+Oath] * Type [OS]	1.17	0.70 – 1.97	0.544
Treatment [Partner] * HH	0.85	0.28 – 2.55	0.769
Treatment [Oath] * HH	1.31	0.44 – 3.91	0.629
Treatment [Partner+Oath] * HH	1.47	0.47 – 4.57	0.507
Type [OS] * HH	1.07	0.62 – 1.85	0.812
(Treatment [Partner] * Type [OS]) * HH	1.14	0.52 – 2.49	0.741
(Treatment [Oath] * Type [OS]) * HH	0.80	0.36 – 1.80	0.591
(Treatment [Partner+Oath] * Type [OS]) * HH	0.62	0.27 – 1.43	0.262
$N_{as.factor}$	1541		
Observations	3896		

Supplementary Table 20. Overview of the main model including honesty-humility in Study 4.

	**Dependent variable**		
Predictors	*Odds Ratios*	*CI*	*p*
1|2	0.04	0.03 – 0.05	**<0.001**

2|3	0.13	0.11 – 0.15	**<0.001**
3|4	0.29	0.25 – 0.35	**<0.001**
4|5	0.69	0.59 – 0.81	**<0.001**
5|6	2.08	1.77 – 2.44	**<0.001**
Treatment [Partner]	1.14	0.90 – 1.44	0.274
Treatment [Oath]	0.73	0.58 – 0.90	**0.004**
Treatment [Partner+Oath]	0.76	0.60 – 0.96	**0.021**
HH	0.74	0.57 – 0.95	**0.019**
Treatment [Partner] * HH	1.00	0.70 – 1.45	0.982
Treatment [Oath] * HH	1.00	0.70 – 1.44	0.983
Treatment [Partner+Oath] * HH	0.80	0.55 – 1.17	0.255
N $_{as.factor}$	1541		
Observations	3896		

11.5 Study 5

Supplementary Table 21. Overview of main model including type of rounds (four rounds: 1, one-shot: 2) in Study 5.

	Dependent variable		
Predictors	*Odds Ratios*	*CI*	*p*
1|2	0.10	0.06 – 0.17	**<0.001**
2|3	0.29	0.18 – 0.46	**<0.001**
3|4	0.64	0.40 – 1.03	0.067
4|5	1.52	0.95 – 2.44	0.083
5|6	4.10	2.55 – 6.61	**<0.001**
Treatment [Partner]	1.07	0.54 – 2.11	0.846
Treatment [Oath]	0.80	0.41 – 1.54	0.504
Treatment [Partner+Oath]	1.14	0.58 – 2.24	0.707
Type [One-Shot]	1.33	0.92 – 1.92	0.124
Treatment [Partner] * Type	1.18	0.70 – 2.00	0.537
Treatment [Oath] * Type	0.96	0.58 – 1.60	0.874
Treatment [Partner+Oath] * Type	0.98	0.58 – 1.66	0.944
$N_{as.factor}$	982		

Observations	2491

Supplementary Table 22. Overview of the main model including treatment, type (1 = four rounds, 2 = one-shot), and honesty-humility (centered) and its interactions in Study 5.

	**Dependent variable**		
Predictors	*Odds Ratios*	*CI*	*p*
1|2	0.10	0.06 – 0.17	**<0.001**
2|3	0.29	0.18 – 0.46	**<0.001**
3|4	0.65	0.41 – 1.05	0.076
4|5	1.54	0.96 – 2.47	0.074
5|6	4.16	2.58 – 6.69	**<0.001**
Treatment [Partner]	1.11	0.56 – 2.19	0.763
Treatment [Oath]	0.81	0.42 – 1.56	0.520
Treatment [Partner+Oath]	1.15	0.58 – 2.26	0.684
Type [One-Shot]	1.35	0.93 – 1.94	0.111
Honesty-Humility (HH)	1.14	0.51 – 2.55	0.747
Treatment [Partner] * Type [OS]	1.15	0.68 – 1.94	0.614

Treatment [Oath] * Type [OS]	0.95	0.57 – 1.59	0.850
Treatment [Partner+Oath] * Type [OS]	0.97	0.57 – 1.65	0.917
Treatment [Partner] * HH	1.59	0.49 – 5.19	0.442
Treatment [Oath] * HH	0.60	0.20 – 1.83	0.369
Treatment [Partner+Oath] * HH	0.80	0.26 – 2.52	0.706
Type [OS] * HH	0.80	0.42 – 1.51	0.486
(Treatment [Partner] * Type [OS]) * HH	0.80	0.32 – 1.99	0.625
(Treatment [Oath] * Type [OS]) * HH	1.46	0.59 – 3.59	0.411
(Treatment [Partner+Oath] * Type [OS]) * HH	1.39	0.56 – 3.48	0.478
$N_{as.factor}$	982		
Observations	2491		

Supplementary Table 23. Overview of the main model including honesty-humility in Study 5.

Dependent variable			
Predictors	*Odds Ratios*	*CI*	*p*

1|2	0.07	0.06 – 0.09	**<0.001**
2|3	0.20	0.17 – 0.24	**<0.001**
3|4	0.46	0.39 – 0.54	**<0.001**
4|5	1.07	0.92 – 1.25	0.384
5|6	2.89	2.45 – 3.40	**<0.001**
Treatment [Partner]	1.32	1.06 – 1.64	**0.014**
Treatment [Oath]	0.76	0.62 – 0.94	**0.012**
Treatment [Partner+Oath]	1.11	0.89 – 1.38	0.351
HH	0.88	0.68 – 1.13	0.306
Treatment [Partner] * HH	1.15	0.79 – 1.68	0.468
Treatment [Oath] * HH	0.93	0.66 – 1.32	0.682
Treatment [Partner+Oath] * HH	1.17	0.82 – 1.67	0.386
$N_{as.factor}$	982		
Observations	2491		

11.6 Study 6.

Supplementary Table 24. Overview of the main model including treatment and type (as nested in treatment) in Study 6.

	**Rating**		
Predictors	*Odds Ratios*	*CI*	*p*
1|2	0.05	0.03 – 0.07	**<0.001**
2|3	0.12	0.09 – 0.16	**<0.001**
3|4	0.26	0.20 – 0.34	**<0.001**
4|5	0.63	0.49 – 0.79	**<0.001**
5|6	1.68	1.32 – 2.13	**<0.001**
Treatment [Partner - Shared]	0.91	0.59 – 1.43	0.687
Treatment [Partner - Double]	1.01	0.64 – 1.60	0.962
Treatment [Oath]	1.16	0.82 – 1.65	0.400
Treatment [Partner+Oath - Shared]	0.68	0.43 – 1.07	0.095
Treatment [Partner+Oath - Double]	1.32	0.85 – 2.05	0.223
Observations	755		

R^2 Nagelkerke	0.010

Note. Comparison condition is baseline treatment.

Supplementary Table 25. Overview of the main model including treatment and interaction with honesty-humility (centered) in Study 6.

	**Rating**		
Predictors	*Odds Ratios*	*CI*	*p*
1|2	0.04	0.03 – 0.07	**<0.001**
2|3	0.11	0.08 – 0.15	**<0.001**
3|4	0.25	0.19 – 0.32	**<0.001**
4|5	0.60	0.47 – 0.76	**<0.001**
5|6	1.62	1.28 – 2.06	**<0.001**
Treatment [Partner]	0.92	0.64 – 1.32	0.661
Treatment [Oath]	1.12	0.79 – 1.59	0.539
Treatment [Partner+Oath]	0.91	0.64 – 1.30	0.614
HH	0.58	0.41 – 0.80	**0.001**
Treatment [Partner] * HH	1.24	0.76 – 2.02	0.397
Treatment [Oath] * HH	1.96	1.20 – 3.21	**0.007**

Treatment [Partner+Oath] * HH	1.11	0.66 – 1.87	0.683
Observations	755		
R^2 Nagelkerke	0.028		

11.7 Study 7.

Supplementary Table 26. Overview of the main model including treatment and interaction with honesty-humility (centered) in Study 7. Outcome refers to reported ratings (Player A only).

	**Rating**		
Predictors	*Odds Ratios*	*CI*	*p*
1|2	0.13	0.11 – 0.14	**<0.001**
2|3	0.32	0.29 – 0.35	**<0.001**
3|4	0.66	0.61 – 0.73	**<0.001**
4|5	1.36	1.24 – 1.49	**<0.001**
5|6	3.48	3.17 – 3.83	**<0.001**
Treatment [Partner]	1.17	0.98 – 1.39	0.089
Treatment [Oath]	0.84	0.74 – 0.96	**0.009**
Treatment [Partner+Oath]	0.99	0.83 – 1.19	0.943
HH c	0.76	0.67 – 0.85	**<0.001**
Treatment [Partner] × HH c	1.09	0.87 – 1.37	0.460
Treatment [Oath] × HH c	1.09	0.91 – 1.30	0.340
Treatment [Partner+Oath] × HH c	0.94	0.75 – 1.18	0.594
**Random Effects**			
σ^2	3.29		
$\tau_{00\ ID\_i}$	0.74		
ICC	0.18		
N_{ID_i}	1193		
Observations	20360		
Marginal R^2 / Conditional R^2	0.012 / 0.193		

Supplementary Table 27. Overview of the main model including treatment and interaction with honesty-humility (centered) in Study 7. Outcome refers to reported doubles.

	Double		
Predictors	*Odds Ratios*	*CI*	*p*
(Intercept)	0.37	0.31 – 0.43	**<0.001**
Treatment [Partner]	1.34	1.00 – 1.79	**0.048**
Treatment [Oath]	0.78	0.62 – 0.97	**0.025**
Treatment [Partner+Oath]	1.37	1.03 – 1.83	**0.029**
HH c	0.59	0.48 – 0.73	**<0.001**
Treatment [Partner] × HH c	0.76	0.52 – 1.12	0.164
Treatment [Oath] × HH c	1.06	0.78 – 1.43	0.711
Treatment [Partner+Oath] × HH c	0.66	0.44 – 0.99	**0.043**
**Random Effects**			
σ^2	3.29		
$\tau_{00\ ID}$	1.90		
ICC	0.37		
N ID	1190		
Observations	11900		
Marginal R^2 / Conditional R^2	0.048 / 0.396		

12. Honesty-Humility

Moderation by Trait Honesty-Humility. We also investigated whether dishonest behavior was influenced by participant's general disposition to act honestly. Across all studies, we observed that trait honesty-humility correlated negatively with dishonest behavior (*r* = -.11 [-.15, -.06], see Supplementary Figure 8 for a detailed overview). However, we did not observe any consistent pattern that this relationship differed across the different treatments (see Supplementary Figure S12.1 and Supplementary Material 11). Overall, the effect was smallest in the *oath* treatment (*r* = -.06 [-.12, .00]).

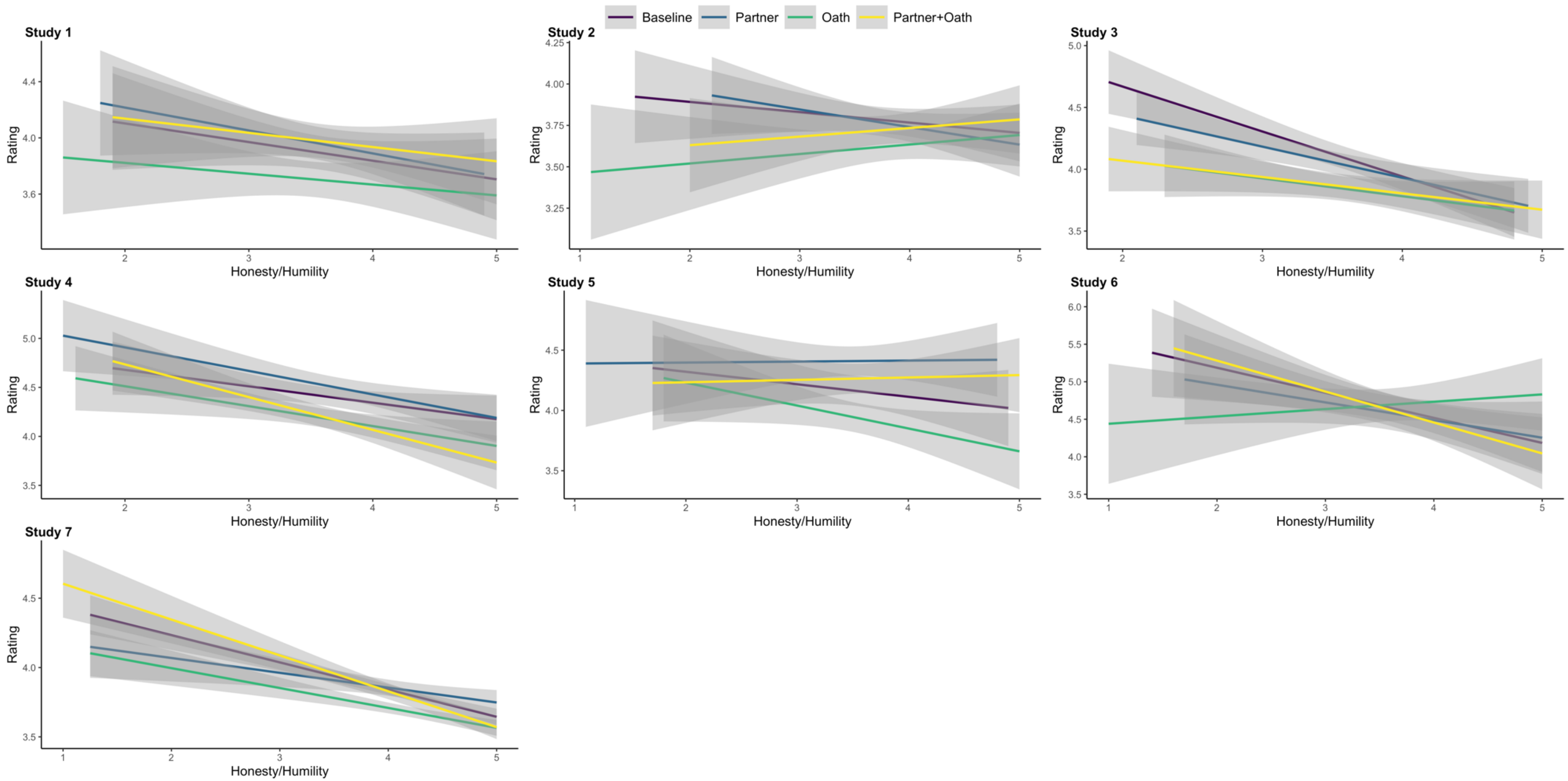

Supplementary Figure 7. Overview of relationship between honesty-humility and ratings across the different treatments and studies. Shaded areas provide 95% confidence intervals.

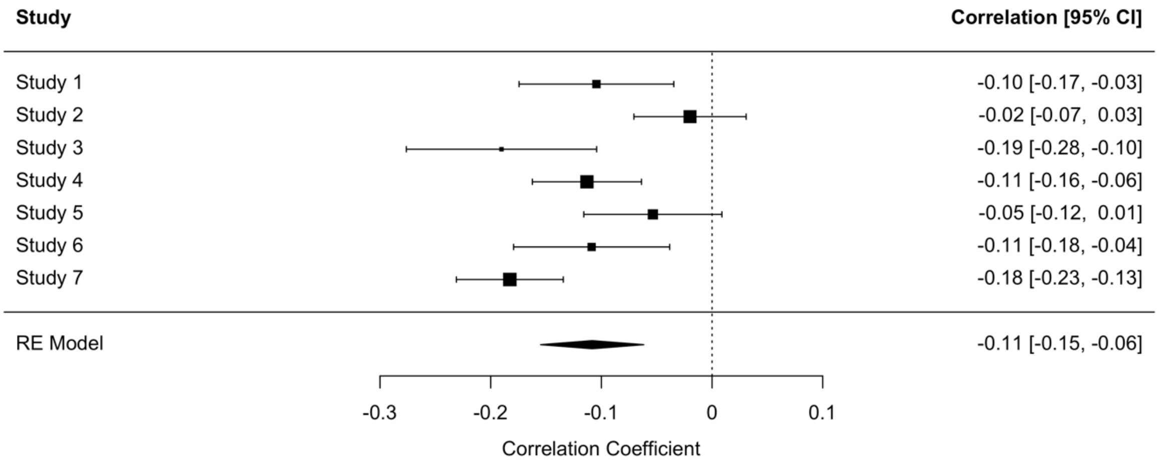

Supplementary Figure 8. Random effects meta-analysis of the correlation between honesty-humility and performance across the seven studies. Parenthesis indicate 95% confidence intervals.

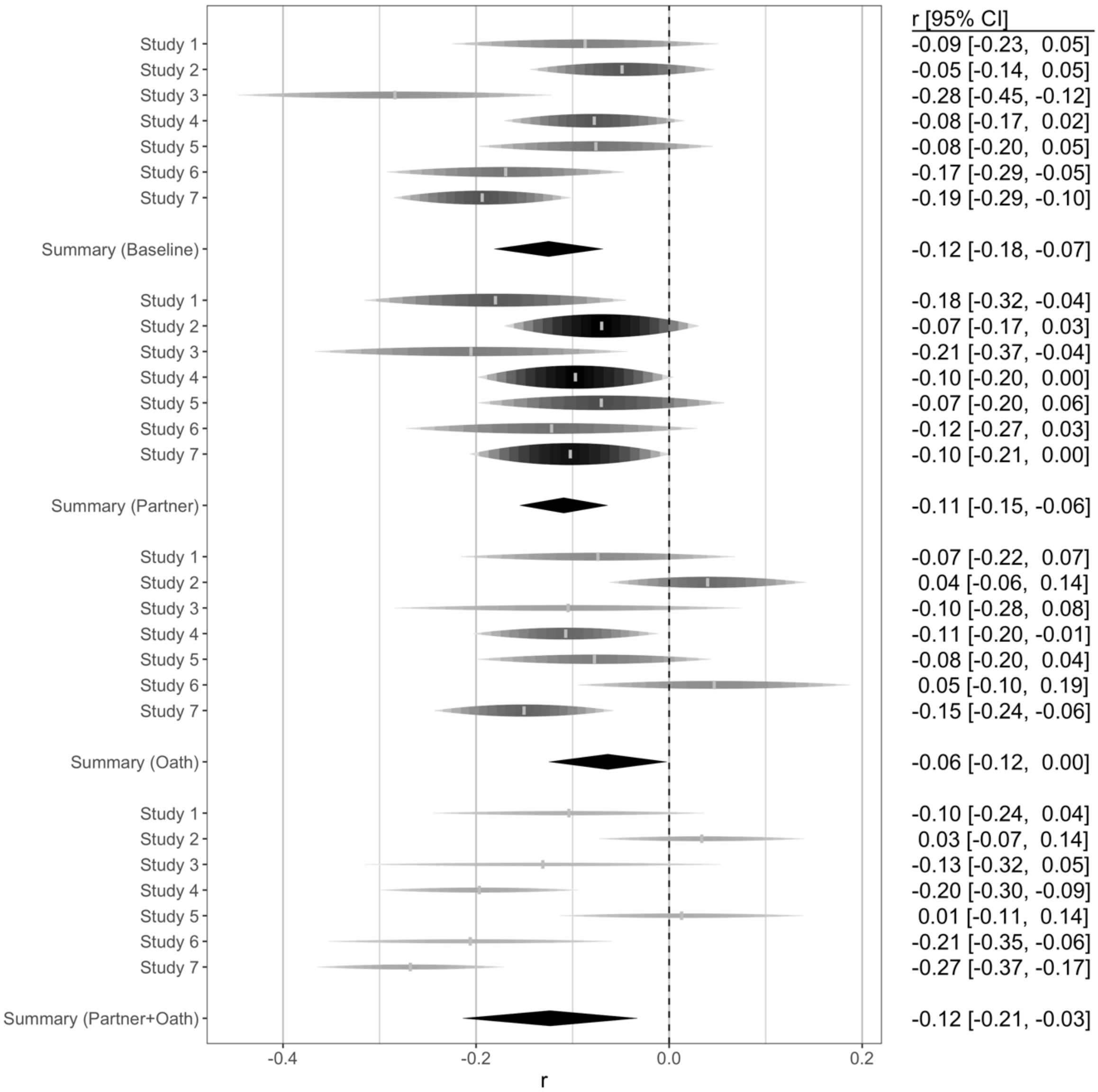

Supplementary Figure 9. Random effects meta-analysis of the correlation between honesty-humility and performance per treatment across the seven studies. Parenthesis indicate 95% confidence intervals.

13. Trial Effects

Development over time. We also explored a possible effect over trials in Studies 1-5. We observed no statistically significant effects with time in any of the studies and there were no specific consistent effects over time apparent in any of the studies. A detailed overview is presented in the Supplementary Figure 10.

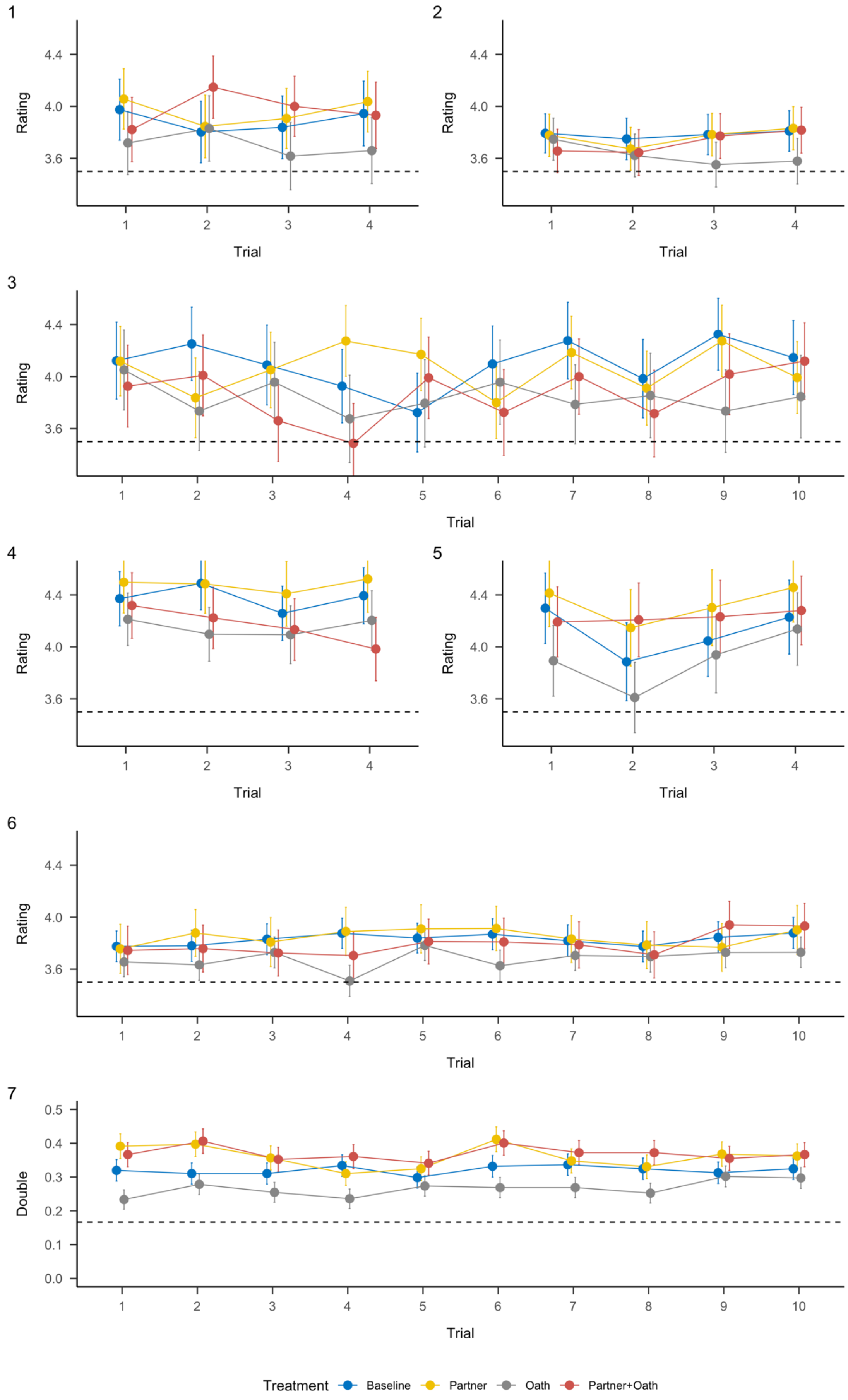
1
2
3
4
5
6
7
Rating
Double
Trial
4.4
4.0
3.6
0.5
0.4
0.3
0.2
0.1
0.0
1
2
3
4
5
6
7
8
9
10
Treatment
Baseline
Partner
Oath
Partner+Oath

Supplementary Figure 10. Overview of development of ratings over trials across Studies 1-5 and 7. Error bars represent 95% confidence intervals. Dotted horizontal line signifies level of full honesty.6 = Study 7 reported die roll ratings (for Player A only in the partner and partner + oath treatments); 7 = Study 7 reported doubles (for group in partner and partner + oath treatments).

13.1 Study 1.

Supplementary Table 28. Overview of model including treatment, target, and trial order in Study 1.

	**Dependent variable**		
Predictors	*Odds Ratios*	*CI*	*p*
1|2	0.07	0.05 – 0.10	**<0.001**
2|3	0.20	0.14 – 0.28	**<0.001**
3|4	0.42	0.30 – 0.59	**<0.001**
4|5	0.94	0.67 – 1.31	0.695
5|6	2.54	1.81 – 3.54	**<0.001**
Treatment [Partner]	1.14	0.71 – 1.82	0.582
Treatment [Oath]	0.84	0.52 – 1.35	0.471
Treatment [Partner+Oath]	1.01	0.63 – 1.61	0.980
Target [Other]	0.67	0.43 – 1.04	0.075
Trial Order (TO)	0.98	0.88 – 1.10	0.758

Treatment [Partner] * Target [Other]	0.98	0.53 – 1.82	0.950
Treatment [Oath] * Target [Other]	1.07	0.57 – 2.01	0.823
Treatment [Partner+Oath] * Target [Other]	1.07	0.58 – 2.00	0.824
Treatment [Partner] * TO	0.99	0.84 – 1.16	0.882
Treatment [Oath] * TO	1.03	0.87 – 1.21	0.756
Treatment [Partner+Oath] * TO	1.01	0.86 – 1.18	0.936
Target [Other] * TO	1.03	0.88 – 1.21	0.730
(Treatment [Partner] * Target [Other]) * TO	0.99	0.79 – 1.25	0.962
(Treatment [Oath] * Target [Other]) * TO	0.93	0.74 – 1.17	0.515
(Treatment [Partner+Oath] * Target [Other]) * TO	1.00	0.80 – 1.26	0.984
$N_{as.factor}$	770		
Observations	6160		

Note. Treatment uses the baseline as comparison, target uses self as the comparison.

Supplementary Table 29. Overview of model including treatment and trial order in Study 1.

	**Dependent variable**		
Predictors	*Odds Ratios*	*CI*	*p*
1|2	0.11	0.08 – 0.15	**<0.001**
2|3	0.30	0.22 – 0.42	**<0.001**
3|4	0.61	0.44 – 0.85	**0.003**
4|5	1.34	0.97 – 1.86	0.075
5|6	3.57	2.57 – 4.96	**<0.001**
Treatment [Partner]	1.08	0.68 – 1.71	0.741
Treatment [Oath]	0.88	0.56 – 1.40	0.598
Treatment [Partner+Oath]	1.06	0.66 – 1.68	0.817
Order Trial (OT)	1.00	0.89 – 1.12	0.988
Treatment [Partner] * OT	0.99	0.85 – 1.16	0.930
Treatment [Oath] * OT	0.96	0.82 – 1.13	0.642

Treatment [Partner+Oath] * OT	1.02	0.87 – 1.19	0.835
N $_{as.factor}$	770		
Observations	3080		

Note. Treatment uses the baseline as comparison.

13.2 Study 2.

Supplementary Table 30. Overview of model including treatment and trial order in Study 2.

	**Dependent variable**		
Predictors	*Odds Ratios*	*CI*	*p*
1|2	0.11	0.09 – 0.14	**<0.001**
2|3	0.35	0.28 – 0.43	**<0.001**
3|4	0.76	0.61 – 0.93	**0.010**
4|5	1.71	1.38 – 2.11	**<0.001**
5|6	4.49	3.62 – 5.58	**<0.001**
Treatment [Partner]	0.93	0.69 – 1.27	0.655
Treatment [Oath]	1.01	0.75 – 1.38	0.935
Treatment [Partner+Oath]	0.81	0.59 – 1.10	0.182

Order Trial (OT)	1.01	0.94 – 1.09	0.771
Treatment [Partner] * OT	1.02	0.91 – 1.14	0.715
Treatment [Oath] * OT	0.93	0.83 – 1.04	0.181
Treatment [Partner+Oath] * OT	1.06	0.95 – 1.19	0.316
$N_{as.factor}$	1494		
Observations	5976		

13.3 Study 3.

Supplementary Table 31. Overview of model including treatment and trial order in Study 3.

	**Dependent variable**		
Predictors	*Odds Ratios*	*CI*	*p*
1|2	0.09	0.07 – 0.11	**<0.001**
2|3	0.25	0.19 – 0.32	**<0.001**
3|4	0.54	0.42 – 0.69	**<0.001**
4|5	1.19	0.92 – 1.54	0.179
5|6	3.07	2.37 – 3.97	**<0.001**

Treatment [Partner]	1.02	0.72 – 1.45	0.911
Treatment [Oath]	0.84	0.58 – 1.21	0.341
Treatment [Partner+Oath]	0.70	0.49 – 1.02	0.061
Order Trial (OT)	1.01	0.98 – 1.05	0.408
Treatment [Partner] * OT	0.99	0.94 – 1.04	0.595
Treatment [Oath] * OT	0.98	0.93 – 1.03	0.374
Treatment [Partner+Oath] * OT	1.01	0.96 – 1.06	0.715
N as.factor	484		
Observations	4840		

13.4 Study 4.

Supplementary Table 32. Overview of model including treatment and trial order in Study 4.

	**Dependent variable**		
Predictors	*Odds Ratios*	*CI*	*p*
1|2	0.04	0.03 – 0.05	**<0.001**

2|3	0.11	0.09 – 0.15	**<0.001**
3|4	0.27	0.20 – 0.35	**<0.001**
4|5	0.63	0.49 – 0.82	**<0.001**
5|6	1.89	1.46 – 2.45	**<0.001**
Treatment [Partner]	1.02	0.70 – 1.50	0.919
Treatment [Oath]	0.68	0.47 – 0.98	**0.037**
Treatment [Partner+Oath]	1.01	0.69 – 1.49	0.945
Order Trial (OT)	0.96	0.87 – 1.06	0.392
Treatment [Partner] * OT	1.04	0.89 – 1.21	0.617
Treatment [Oath] * OT	1.03	0.90 – 1.18	0.677
Treatment [Partner+Oath] * OT	0.88	0.76 – 1.02	0.087
$N_{as.factor}$	1541		
Observations	3896		

13.5 Study 5.

Supplementary Table 33. Overview of model including treatment and trial order in Study 5.

	**Dependent variable**		
Predictors	*Odds Ratios*	*CI*	*p*
1|2	0.06	0.05 – 0.09	**<0.001**
2|3	0.18	0.13 – 0.25	**<0.001**
3|4	0.41	0.30 – 0.55	**<0.001**
4|5	0.96	0.71 – 1.30	0.803
5|6	2.60	1.91 – 3.53	**<0.001**
Treatment [Partner]	1.30	0.84 – 2.00	0.240
Treatment [Oath]	0.60	0.40 – 0.92	**0.019**
Treatment [Partner+Oath]	1.03	0.67 – 1.59	0.890
Order Trial (OT)	0.95	0.84 – 1.07	0.428
Treatment [Partner] * OT	1.01	0.85 – 1.20	0.948
Treatment [Oath] * OT	1.11	0.94 – 1.32	0.216
Treatment [Partner+Oath] * OT	1.03	0.87 – 1.23	0.701

$N_{as.factor}$	982
Observations	2491

13.6 Study 7.

Supplementary Table 34. Overview of model including treatment and round in Study 7. Response refers to reported die roll ratings (for Player A only in the partner and partner+oath treatments).

		**Rating**	
Predictors	*Odds Ratios*	*CI*	*p*
1|2	0.13	0.11 – 0.15	**<0.001**
2|3	0.33	0.29 – 0.37	**<0.001**
3|4	0.68	0.60 – 0.77	**<0.001**
4|5	1.39	1.23 – 1.57	**<0.001**
5|6	3.57	3.16 – 4.03	**<0.001**
Treatment [Partner]	1.27	0.99 – 1.64	0.065
Treatment [Oath]	0.80	0.68 – 0.95	**0.009**
Treatment [Partner+Oath]	0.96	0.75 – 1.24	0.774
Round	1.01	0.99 – 1.02	0.281
Treatment [Partner] × Round	0.98	0.95 – 1.01	0.267
Treatment [Oath] × Round	1.00	0.98 – 1.02	0.711
Treatment [Partner+Oath] × Round	1.00	0.97 – 1.04	0.764
**Random Effects**			
σ^2	3.29		
$\tau_{00\ ID\_i}$	0.77		
ICC	0.19		
N_{ID_i}	1193		
Observations	20360		
Marginal R^2 / Conditional R^2	0.004 / 0.193		

Supplementary Table 35. Overview of model including treatment and round in Study 7. Response refers to reported doubles (for whole group in terms of partner and partner+oath treatment).

	**Double**		
Predictors	*Odds Ratios*	*CI*	*p*
(Intercept)	0.36	0.29 – 0.45	**<0.001**
Treatment [Partner]	1.49	0.99 – 2.24	0.053
Treatment [Oath]	0.62	0.45 – 0.85	**0.003**
Treatment [Partner+Oath]	1.34	0.90 – 1.99	0.148
Round	1.01	0.98 – 1.03	0.622
Treatment [Partner] × Round	0.98	0.93 – 1.03	0.476
Treatment [Oath] × Round	1.03	0.99 – 1.07	0.169
Treatment [Partner+Oath] × Round	0.99	0.94 – 1.03	0.562
**Random Effects**			
σ^2	3.29		
$\tau_{00\ ID}$	2.13		
ICC	0.39		
N_{ID}	1190		
Observations	11900		
Marginal R^2 / Conditional R^2	0.011 / 0.400		

14. Responsibility Models

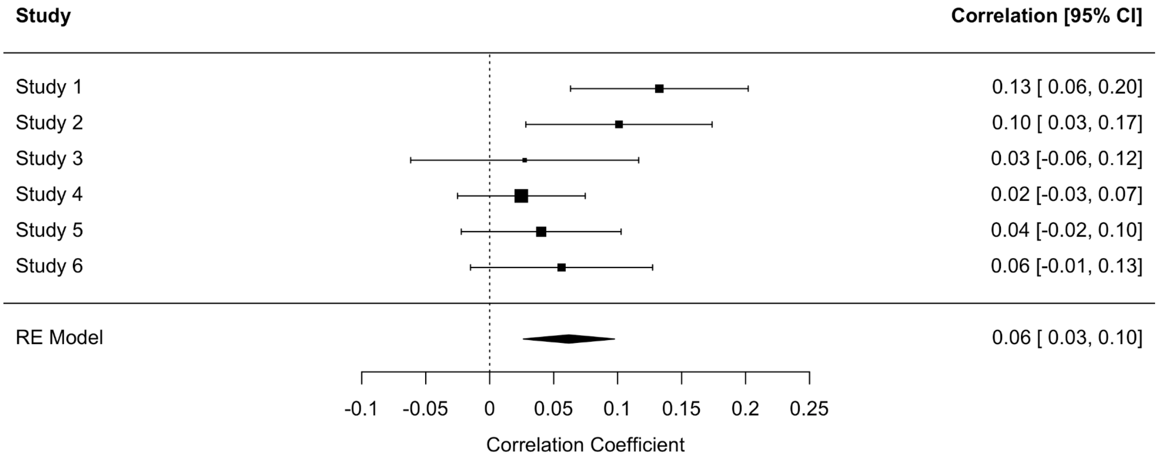

Supplementary Figure 11. Random effects meta-analysis of the correlation between felt responsibility and rating across the six studies. Parenthesis indicate 95% confidence intervals.

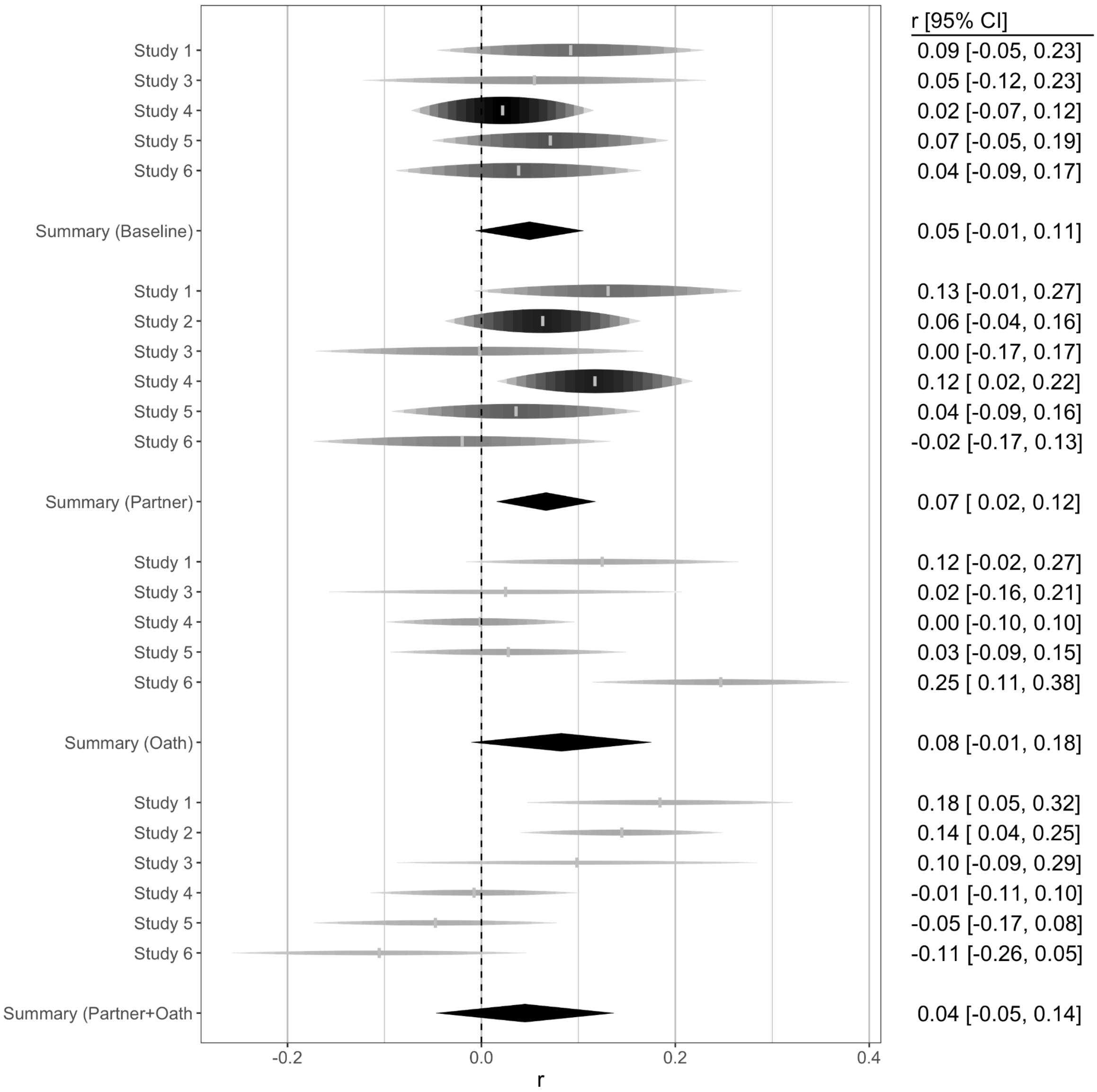

Supplementary Figure 12. Random effects meta-analysis of the correlation between felt responsibility and rating per treatment across the six studies. Parenthesis indicate 95% confidence intervals.

14.1 Study 1.

Supplementary Table 36. Overview of model with responsibility and treatment in Study 1.

	Responsibility				
Predictors	*Estimates*	*std. Beta*	*CI*	*standardized CI*	*p*
(Intercept)	2.98	-0.07	2.82 – 3.14	-0.21 – 0.07	**<0.001**

Treatment [Partner]	0.13	0.11	-0.11 – 0.36	-0.09 – 0.31	0.282
Treatment [Oath]	0.10	0.08	-0.14 – 0.34	-0.12 – 0.28	0.406
Treatment [Partner+Oath]	0.13	0.11	-0.11 – 0.36	-0.09 – 0.31	0.295
Observations	770				
R^2 / R^2 adjusted	0.002 / -0.002				

14.2 Study 2.

Supplementary Table 37. Overview of responsibility by treatment in Study 2.

	Responsibility				
Predictors	*Estimates*	*std. Beta*	*CI*	*standardized CI*	*p*
(Intercept)	3.29	-0.02	3.18 – 3.41	-0.13 – 0.08	**<0.001**
Treatment [Partner+Oath]	0.06	0.05	-0.11 – 0.23	-0.10 – 0.20	0.511
Observations	711				
R^2 / R^2 adjusted	0.001 / -0.001				

Note. Comparison is the partner treatment as baseline and oath were not recorded in this study.

14.3 Study 3.

Supplementary Table 38. Overview of model with responsibility and treatment in Study 3.

	Responsibility				
Predictors	*Estimates*	*std. Beta*	*CI*	*standardized CI*	*p*

(Intercept)	3.48	0.03	3.25 – 3.71	-0.14 – 0.21	**<0.001**
Treatment [Partner]	-0.29	-0.23	-0.61 – 0.02	-0.47 – 0.02	0.069
Treatment [Oath]	0.13	0.10	-0.20 – 0.46	-0.15 – 0.35	0.448
Treatment [Partner+Oath]	0.03	0.03	-0.30 – 0.37	-0.23 – 0.28	0.842
Observations	484				
R^2 / R^2 adjusted	0.016 / 0.009				

Note. Baseline treatment used as comparison.

14.4 Study 4.

Supplementary Table 39. Overview of model with responsibility and treatment in Study 4.

	**Responsibility**				
Predictors	*Estimates*	*std. Beta*	*CI*	*standardized CI*	*p*
(Intercept)	3.57	-0.09	3.46 – 3.68	-0.18 – 0.00	**<0.001**
Treatment [Partner]	0.06	0.06	-0.09 – 0.22	-0.08 – 0.20	0.423
Treatment [Oath]	0.23	0.20	0.08 – 0.39	0.07 – 0.34	**0.003**
Treatment [Partner+Oath]	0.11	0.10	-0.05 – 0.28	-0.04 – 0.24	0.170
Observations	1541				
R^2 / R^2 adjusted	0.006 / 0.004				

Note. Baseline treatment used as comparison.

14.5 Study 5.

Supplementary Table 40. Model with responsibility and treatment in Study 5.

	Responsibility				
Predictors	*Estimates*	*std. Beta*	*CI*	*standardized CI*	*p*
(Intercept)	3.64	-0.18	3.51 – 3.76	-0.30 – -0.06	<0.001
Treatment [Partner]	0.33	0.32	0.15 – 0.51	0.14 – 0.49	<0.001
Treatment [Oath]	0.07	0.07	-0.11 – 0.25	-0.10 – 0.24	0.436
Treatment [Partner+Oath]	0.36	0.35	0.18 – 0.54	0.17 – 0.52	<0.001
Observations	982				
R^2 / R^2 adjusted	0.023 / 0.020				

Note. Baseline treatment used as comparison.

14.6 Study 6.

Supplementary Table 41. Model with responsibility and treatment in Study 6.

	Responsibility				
Predictors	*Estimates*	*std. Beta*	*CI*	*standardized CI*	*p*
(Intercept)	3.65	-0.02	3.48 – 3.82	-0.15 – 0.10	**<0.001**
Treatment [Partner]	0.01	0.01	-0.25 – 0.28	-0.19 – 0.21	0.911
Treatment [Oath]	0.03	0.02	-0.22 – 0.28	-0.17 – 0.21	0.841

Treatment [Partner+Oath]	0.10	0.08	-0.16 – 0.36	-0.12 – 0.28	0.459
Observations	755				
R^2 / R^2 adjusted	0.001 / -0.003				

Note. Baseline treatment used as comparison.

15. Socioeconomic Status

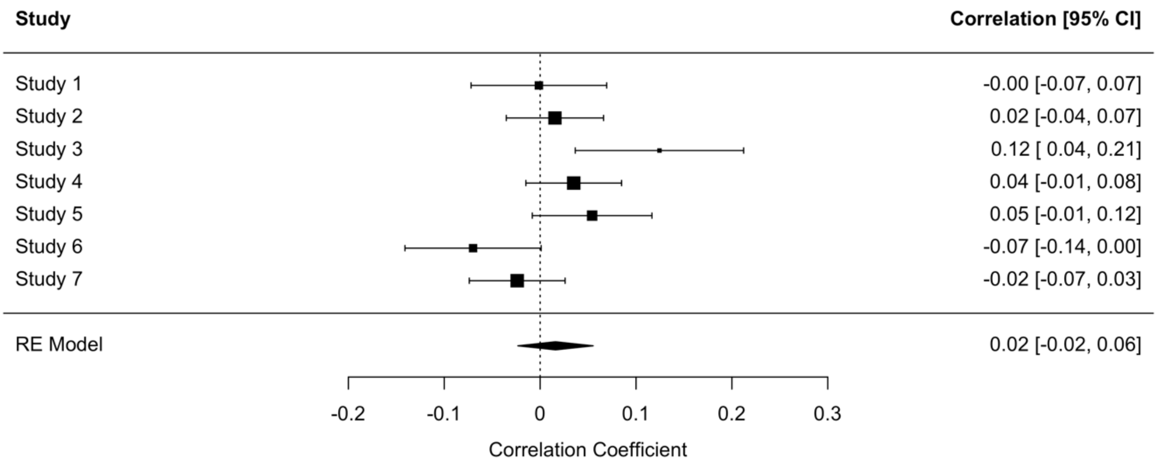

Supplementary Figure 13. Random effects meta-analysis of the correlation between socioeconomic status and rating across the six studies. Parenthesis indicate 95% confidence intervals.

15.1 Study 1.

Supplementary Table 42. Overview of the main model including treatment and its interaction with socioeconomic status (centered) in Study 1.

Dependent variable

Predictors	*Odds Ratios*	*CI*	*p*
1|2	0.11	0.09 – 0.13	**<0.001**
2|3	0.31	0.26 – 0.36	**<0.001**

3|4	0.62	0.52 – 0.73	**<0.001**
4|5	1.35	1.14 – 1.60	**0.001**
5|6	3.60	3.01 – 4.29	**<0.001**
Treatment [Partner]	1.07	0.85 – 1.36	0.545
Treatment [Oath]	0.80	0.63 – 1.02	0.067
Treatment [Partner+Oath]	1.11	0.87 – 1.40	0.400
Socioeconomic Status (SES)	1.04	0.94 – 1.14	0.488
Treatment [Partner] * SES	1.03	0.89 – 1.19	0.692
Treatment [Oath] * SES	0.98	0.85 – 1.13	0.824
Treatment [Partner + Oath] * SES	0.94	0.81 – 1.08	0.395
$N_{as.factor}$	769		
Observations	3076		

15.2 Study 2.

Supplementary Table 43. Overview of the main model including treatment and its interaction with socioeconomic status (centered) in Study 2.

	**Dependent variable**		
Predictors	*Odds Ratios*	*CI*	*p*

1|2	0.11	0.10 – 0.12	**<0.001**
2|3	0.34	0.31 – 0.38	**<0.001**
3|4	0.74	0.67 – 0.81	**<0.001**
4|5	1.66	1.51 – 1.83	**<0.001**
5|6	4.36	3.93 – 4.85	**<0.001**
Treatment [Partner]	0.98	0.86 – 1.12	0.794
Treatment [Oath]	0.84	0.74 – 0.96	**0.013**
Treatment [Partner+Oath]	0.93	0.81 – 1.07	0.292
Socioeconomic Status (SES)	1.00	0.94 – 1.05	0.871
Treatment [Partner] * SES	0.99	0.91 – 1.08	0.848
Treatment [Oath] * SES	1.02	0.94 – 1.12	0.576
Treatment [Partner + Oath] * SES	1.04	0.95 – 1.13	0.399
N as.factor	1494		
Observations	5976		

15.3 Study 3.

Supplementary Table 44. Overview of the main model including treatment and its interaction with socioeconomic status (centered) in Study 3.

	**Dependent variable**		
Predictors	*Odds Ratios*	*CI*	*p*
1|2	0.08	0.07 – 0.10	**<0.001**
2|3	0.23	0.19 – 0.27	**<0.001**
3|4	0.49	0.42 – 0.58	**<0.001**
4|5	1.10	0.93 – 1.30	0.289
5|6	2.82	2.38 – 3.35	**<0.001**
Treatment [Partner]	0.94	0.75 – 1.18	0.612
Treatment [Oath]	0.74	0.58 – 0.94	**0.013**
Treatment [Partner+Oath]	0.74	0.58 – 0.94	**0.012**
Socioeconomic Status (SES)	1.05	0.94 – 1.17	0.360
Treatment [Partner] * SES	1.00	0.86 – 1.16	0.951
Treatment [Oath] * SES	1.06	0.91 – 1.24	0.443
Treatment [Partner + Oath]	1.05	0.90 – 1.24	0.518

* SES	
N as.factor	484
Observations	4840

Note.

15.4 Study 4.

Supplementary Table 45. Overview of the main model including treatment and its interaction with socioeconomic status (centered) in Study 4.

	**Dependent variable**		
Predictors	*Odds Ratios*	*CI*	*p*
1|2	0.04	0.03 – 0.05	**<0.001**
2|3	0.12	0.10 – 0.15	**<0.001**
3|4	0.29	0.24 – 0.34	**<0.001**
4|5	0.68	0.58 – 0.80	**<0.001**
5|6	2.05	1.74 – 2.40	**<0.001**
Treatment [Partner]	1.10	0.87 – 1.39	0.438
Treatment [Oath]	0.72	0.58 – 0.90	**0.003**
Treatment [Partner+Oath]	0.76	0.60 – 0.96	**0.021**

Socioeconomic Status (SES)	1.09	0.99 – 1.21	0.082
Treatment [Partner] * SES	0.95	0.82 – 1.10	0.462
Treatment [Oath] * SES	0.93	0.81 – 1.07	0.301
Treatment [Partner + Oath] * SES	0.95	0.82 – 1.11	0.536
N as.factor	1540		
Observations	3892		

15.5 Study 5

Supplementary Table 46. Overview of the main model including treatment and its interaction with socioeconomic status (centered) in Study 5.

	**Dependent variable**		
Predictors	*Odds Ratios*	*CI*	*p*
1|2	0.07	0.06 – 0.09	**<0.001**
2|3	0.20	0.17 – 0.24	**<0.001**
3|4	0.45	0.38 – 0.53	**<0.001**
4|5	1.06	0.90 – 1.24	0.488
5|6	2.85	2.42 – 3.36	**<0.001**

Treatment [Partner]	1.30	1.04 – 1.62	**0.020**
Treatment [Oath]	0.75	0.61 – 0.93	**0.009**
Treatment [Partner+Oath]	1.09	0.87 – 1.35	0.459
Socioeconomic Status (SES)	1.10	0.99 – 1.22	0.070
Treatment [Partner] * SES	0.92	0.80 – 1.07	0.300
Treatment [Oath] * SES	0.92	0.80 – 1.07	0.306
Treatment [Partner + Oath] * SES	1.00	0.87 – 1.16	0.997
$N_{as.factor}$	982		
Observations	2491		

15.6 Study 6.

Supplementary Table 47. Overview of the main model including treatment and its interaction with socioeconomic status (centered) in Study 6.

	**Rating**		
Predictors	*Odds Ratios*	*CI*	*p*
1|2	0.05	0.03 – 0.07	**<0.001**

2|3	0.12	0.09 – 0.16	**<0.001**
3|4	0.26	0.20 – 0.34	**<0.001**
4|5	0.63	0.49 – 0.79	**<0.001**
5|6	1.67	1.32 – 2.13	**<0.001**
Treatment [Partner]	0.96	0.67 – 1.37	0.806
Treatment [Oath]	1.16	0.82 – 1.65	0.404
Treatment [Partner+Oath]	0.96	0.67 – 1.37	0.815
Socioeconomic Status (SES)	0.99	0.87 – 1.13	0.933
Treatment [Partner] * SES	0.90	0.74 – 1.10	0.323
Treatment [Oath] * SES	0.87	0.71 – 1.08	0.205
Treatment [Partner + Oath] * SES	0.98	0.79 – 1.22	0.879
Observations	755		
R^2 Nagelkerke	0.009		

15.7 Study 7.

Supplementary Table 48. Overview of the main model including treatment and its interaction with socioeconomic status (centered) in Study 7.

	Rating		
Predictors	*Odds Ratios*	*CI*	*p*
1|2	0.12	0.11 – 0.14	**<0.001**
2|3	0.32	0.29 – 0.35	**<0.001**
3|4	0.66	0.60 – 0.72	**<0.001**
4|5	1.34	1.22 – 1.48	**<0.001**
5|6	3.45	3.14 – 3.80	**<0.001**
Treatment [Oath]	0.82	0.72 – 0.94	**0.003**
Treatment [Partner]	1.16	0.97 – 1.39	0.100
Treatment [Partner+Oath]	1.00	0.83 – 1.19	0.961
Socioeconomic Status (SES)	0.99	0.95 – 1.04	0.801
Treatment [Oath] * SES	1.03	0.96 – 1.10	0.377
Treatment [Partner] * SES	0.94	0.84 – 1.04	0.202
Treatment [Partner + Oath] * SES	1.04	0.94 – 1.14	0.481
**Random Effects**			
σ^2	3.29		
τ_{00} ID_i	0.76		
ICC	0.19		
N ID_i	1191		
Observations	20320		
Marginal R^2 / Conditional R^2	0.004 / 0.191		

Supplementary Table 49. Overview of the main model including treatment and its interaction with socioeconomic status (centered) in Study 7. Model for reported double (per participant or group).

	Double		
Predictors	*Odds Ratios*	*CI*	*p*
(Intercept)	0.38	0.32 – 0.44	**<0.001**

Treatment [Partner]	1.36	1.01 – 1.84	**0.045**
Treatment [Oath]	0.73	0.58 – 0.92	**0.007**
Treatment [Partner+Oath]	1.24	0.92 – 1.66	0.157
Socioeconomic Status (SES)	0.93	0.85 – 1.01	0.092
Treatment [Partner] * SES	1.02	0.87 – 1.21	0.800
Treatment [Oath] * SES	1.12	0.99 – 1.26	0.067
Treatment [Partner + Oath] * SES	1.01	0.86 – 1.18	0.949
**Random Effects**			
σ^2	3.29		
$\tau_{00\ ID}$	2.10		
ICC	0.39		
N_{ID}	1188		
Observations	11880		
Marginal R^2 / Conditional R^2	0.012 / 0.397		

16. Gender

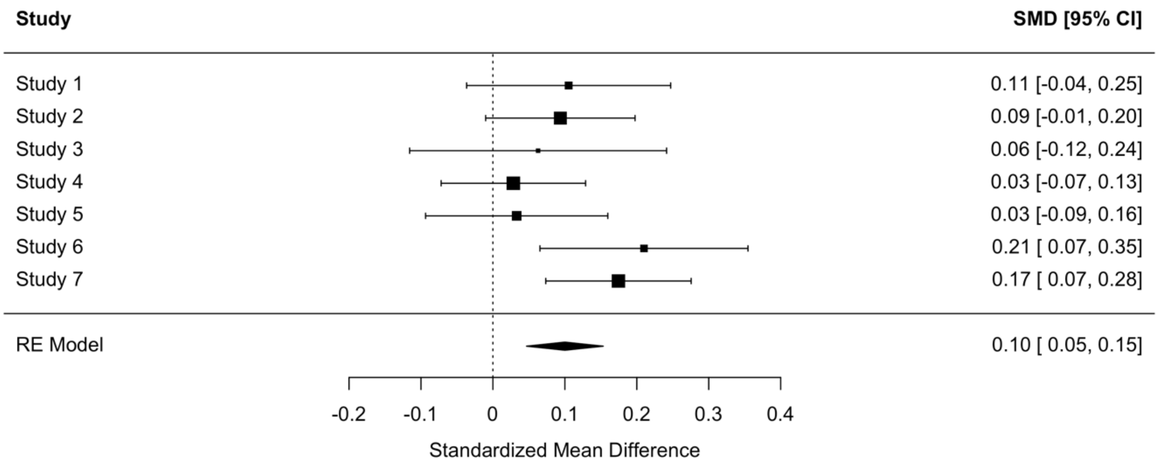

Supplementary Figure 14. Random-effects model of difference between men and women for ratings across the different studies. A positive effect symbolizes on average higher ratings for men.

16.1 Study 1.

Supplementary Table 50. Overview of the main model including treatment and interaction with gender (men = 1, women 2) in Study 1.

	**Dependent variable**		
Predictors	*Odds Ratios*	*CI*	*p*
1|2	0.10	0.08 – 0.13	**<0.001**
2|3	0.27	0.22 – 0.35	**<0.001**
3|4	0.55	0.44 – 0.70	**<0.001**
4|5	1.21	0.96 – 1.52	0.111
5|6	3.21	2.53 – 4.06	**<0.001**
Treatment [Partner]	0.97	0.70 – 1.34	0.837
Treatment [Oath]	0.77	0.55 – 1.07	0.119
Treatment [Partner+Oath]	1.03	0.74 – 1.44	0.853
Gender (1 = men, 2 = women)	0.79	0.57 – 1.10	0.167
Treatment [Partner] * Gender	1.24	0.78 – 1.98	0.358
Treatment [Oath] * Gender	1.10	0.69 – 1.77	0.686

Treatment [Partner+Oath] * Gender	1.14	0.71 – 1.82	0.588
N as.factor	765		
Observations	3060		

16.2 Study 2.

Supplementary Table 51. Overview of the main model including treatment and interaction with gender (men = 1, women = 2) in Study 2.

	**Dependent variable**		
Predictors	*Odds Ratios*	*CI*	*p*
1|2	0.09	0.08 – 0.11	**<0.001**
2|3	0.30	0.26 – 0.35	**<0.001**
3|4	0.65	0.56 – 0.75	**<0.001**
4|5	1.46	1.26 – 1.69	**<0.001**
5|6	3.84	3.28 – 4.48	**<0.001**
Treatment [Partner]	1.00	0.81 – 1.24	1.000
Treatment [Oath]	0.76	0.62 – 0.94	**0.012**
Treatment [Partner+Oath]	0.72	0.58 – 0.90	**0.003**
Gender (1 = men, 2 = women)	0.81	0.67 – 0.98	**0.028**

Treatment [Partner] * Gender	0.95	0.73 – 1.25	0.721
Treatment [Oath] * Gender	1.16	0.88 – 1.52	0.298
Treatment [Partner+Oath] * Gender	1.52	1.15 – 2.01	**0.003**
N as.factor	1494		
Observations	5976		

16.3 Study 3.

Supplementary Table 52. Overview of the main model including treatment and interaction with gender (men = 1, women = 2) in Study 3.

	Dependent variable		
Predictors	*Odds Ratios*	*CI*	*p*
1|2	0.08	0.06 – 0.10	**<0.001**
2|3	0.22	0.17 – 0.28	**<0.001**
3|4	0.48	0.38 – 0.62	**<0.001**
4|5	1.07	0.83 – 1.37	0.600
5|6	2.74	2.13 – 3.51	**<0.001**
Treatment [Partner]	0.96	0.69 – 1.34	0.831
Treatment [Oath]	0.84	0.60 – 1.18	0.316

Treatment [Partner+Oath]	0.68	0.48 – 0.95	**0.026**
Gender (1 = men, 2 = women)	0.95	0.68 – 1.32	0.742
Treatment [Partner] * Gender	0.94	0.60 – 1.49	0.801
Treatment [Oath] * Gender	0.75	0.47 – 1.21	0.240
Treatment [Partner+Oath] * Gender	1.20	0.74 – 1.94	0.457
N as.factor	482		
Observations	4820		

16.4 Study 4.

Supplementary Table 53. Overview of the main model including treatment and interaction with gender (men = 1, women = 2) in Study 4.

	**Dependent variable**		
Predictors	*Odds Ratios*	*CI*	*p*
1|2	0.04	0.03 – 0.05	**<0.001**
2|3	0.13	0.10 – 0.16	**<0.001**
3|4	0.29	0.23 – 0.36	**<0.001**
4|5	0.69	0.55 – 0.85	**0.001**

5|6	2.04	1.64 – 2.54	**<0.001**
Treatment [Partner]	1.19	0.86 – 1.66	0.288
Treatment [Oath]	0.73	0.54 – 0.99	**0.044**
Treatment [Partner+Oath]	0.75	0.55 – 1.04	0.086
Gender (1 = men, 2 = women)	0.98	0.72 – 1.34	0.911
Treatment [Partner] * Gender	0.89	0.56 – 1.42	0.624
Treatment [Oath] * Gender	0.98	0.63 – 1.52	0.918
Treatment [Partner+Oath] * Gender	1.03	0.64 – 1.64	0.912
$N_{as.factor}$	1525		
Observations	3847		

16.5 Study 5.

Supplementary Table 54. Overview of the main model including treatment and interaction with gender (men = 1, women = 2) in Study 5.

	**Dependent variable**		
Predictors	*Odds Ratios*	*CI*	*p*

1|2	0.07	0.05 – 0.09	**<0.001**
2|3	0.19	0.15 – 0.25	**<0.001**
3|4	0.43	0.34 – 0.55	**<0.001**
4|5	1.02	0.81 – 1.29	0.850
5|6	2.77	2.19 – 3.51	**<0.001**
Treatment [Partner]	1.36	0.99 – 1.87	0.057
Treatment [Oath]	0.69	0.50 – 0.94	**0.019**
Treatment [Partner+Oath]	1.06	0.77 – 1.45	0.739
Gender (1 = men, 2 = women)	0.93	0.68 – 1.26	0.634
Treatment [Partner] * Gender	0.94	0.60 – 1.47	0.791
Treatment [Oath] * Gender	1.12	0.73 – 1.73	0.598
Treatment [Partner+Oath] * Gender	1.09	0.70 – 1.69	0.703
$N_{as.factor}$	958		
Observations	2434		

16.6. Study 6.

Supplementary Table 55. Overview of the main model including treatment and interaction with gender (men = 1, women = 2) in Study 6.

	**Rating**		
Predictors	*Odds Ratios*	*CI*	*p*
1|2	0.04	0.02 – 0.06	**<0.001**
2|3	0.10	0.07 – 0.14	**<0.001**
3|4	0.21	0.15 – 0.30	**<0.001**
4|5	0.51	0.36 – 0.71	**<0.001**
5|6	1.36	0.97 – 1.90	0.073
Treatment [Partner]	0.67	0.39 – 1.15	0.150
Treatment [Oath]	1.12	0.68 – 1.85	0.662
Treatment [Partner+Oath]	1.06	0.65 – 1.74	0.801
Gender (1 = men, 2 = women)	0.64	0.40 – 1.01	0.058
Treatment [Partner] * Gender	2.09	1.01 – 4.33	**0.048**

Treatment [Oath] * Gender	1.02	0.50 – 2.07	0.959
Treatment [Partner+Oath] * Gender	0.71	0.35 – 1.47	0.360
Observations	739		
R^2 Nagelkerke	0.022		

16.5 Study 7.

Supplementary Table 56. Overview of the main model including treatment and interaction with gender (men = 1, women = 2) in Study 7. For die roll rating (for pattner and partner+oath only for Player A).

	Rating		
Predictors	*Odds Ratios*	*CI*	*p*
1|2	0.11	0.09 – 0.13	**<0.001**
2|3	0.28	0.24 – 0.32	**<0.001**
3|4	0.58	0.50 – 0.66	**<0.001**
4|5	1.18	1.02 – 1.35	**0.021**
5|6	3.02	2.62 – 3.47	**<0.001**
Treatment [Oath]	0.78	0.64 – 0.94	**0.009**
Treatment [Partner]	1.08	0.84 – 1.38	0.551
Treatment [Partner+Oath]	0.99	0.76 – 1.29	0.921
Gender (1 = men, 2 = women)	0.79	0.65 – 0.95	**0.014**
Treatment [Oath] * Gender	1.09	0.84 – 1.42	0.519
Treatment [Partner] * Gender	1.05	0.72 – 1.51	0.806
Treatment [Partner+Oath] * Gender	1.00	0.69 – 1.44	0.993
**Random Effects**			
σ^2	3.29		

$\tau_{00\ ID\_i}$	0.77
ICC	0.19
N_{ID_i}	1170
Observations	19950
Marginal R^2 / Conditional R^2	0.006 / 0.194

Supplementary Table 57. Overview of the main model including treatment and interaction with gender (men = 1, women = 2) in Study 7. For reported doubles.

	**Double**		
Predictors	*Odds Ratios*	*CI*	*p*
(Intercept)	0.48	0.38 – 0.61	**<0.001**
Treatment [Partner]	1.59	1.05 – 2.41	**0.030**
Treatment [Oath]	0.64	0.46 – 0.89	**0.008**
Treatment [Partner+Oath]	1.19	0.78 – 1.81	0.426
Gender (1 = men, 2 = women)	0.62	0.45 – 0.86	**0.004**
Treatment [Partner] * Gender	0.69	0.38 – 1.27	0.233
Treatment [Oath] * Gender	1.23	0.77 – 1.94	0.383
Treatment [Partner+Oath] * Gender	1.00	0.55 – 1.80	0.991
**Random Effects**			
σ^2	3.29		
$\tau_{00\ ID}$	2.06		
ICC	0.39		
N_{ID}	1166		
Observations	11660		
Marginal R^2 / Conditional R^2	0.021 / 0.399		

17. Age

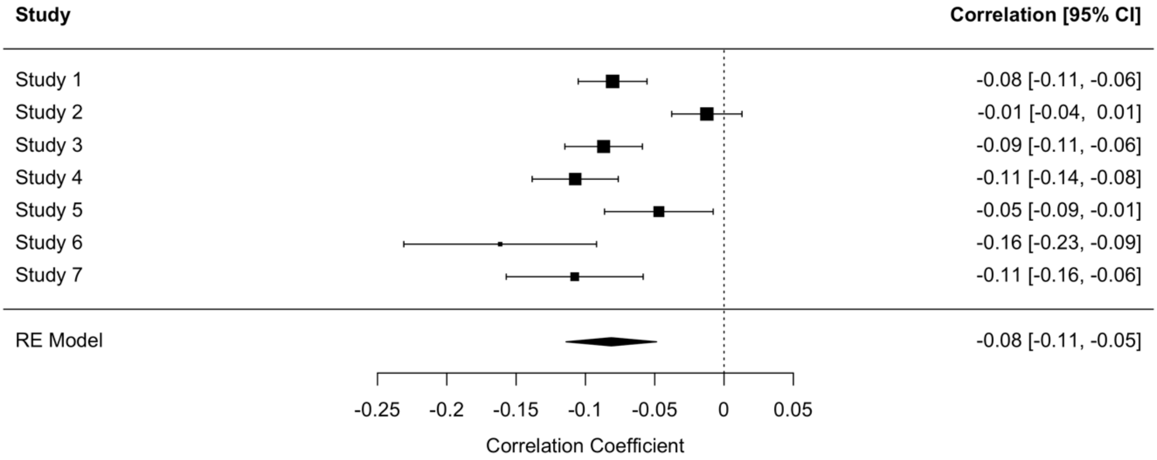

Supplementary Figure 15. Random-effects model of correlation between age and rating across the seven studies.

17.2 Study 2.

Supplementary Table 58. Overview of the main model including age (centered) in Study 2.

Dependent variable

Predictors	*Odds Ratios*	*CI*	*p*
1|2	0.11	0.10 – 0.12	**<0.001**
2|3	0.34	0.31 – 0.38	**<0.001**
3|4	0.74	0.67 – 0.81	**<0.001**
4|5	1.66	1.51 – 1.83	**<0.001**
5|6	4.36	3.93 – 4.85	**<0.001**
Treatment [Partner]	0.98	0.86 – 1.12	0.788

Treatment [Oath]	0.84	0.73 – 0.96	**0.011**
Treatment [Partner+Oath]	0.93	0.81 – 1.07	0.311
Age	1.00	0.99 – 1.01	0.940
Treatment [Partner] * Age	1.00	0.99 – 1.01	0.976
Treatment [Oath] * Age	1.00	0.99 – 1.01	0.689
Treatment [Partner+Oath] * Age	1.00	0.99 – 1.01	0.572
$N_{as.factor}$	1494		
Observations	5976		

17.3 Study 3.

Supplementary Table 59. Overview of the main model including age (centered) in Study 3.

	**Dependent variable**		
Predictors	*Odds Ratios*	*CI*	*p*
1|2	0.08	0.07 – 0.10	**<0.001**
2|3	0.22	0.19 – 0.27	**<0.001**
3|4	0.49	0.41 – 0.58	**<0.001**

4|5	1.09	0.92 – 1.28	0.325
5|6	2.80	2.36 – 3.32	**<0.001**
Treatment [Partner]	0.92	0.74 – 1.16	0.493
Treatment [Oath]	0.74	0.59 – 0.94	**0.012**
Treatment [Partner+Oath]	0.73	0.58 – 0.93	**0.010**
Age	0.98	0.97 – 0.99	**0.001**
Treatment [Partner] * Age	1.01	0.99 – 1.02	0.411
Treatment [Oath] * Age	1.01	0.99 – 1.03	0.394
Treatment [Partner+Oath] * Age	1.01	0.99 – 1.03	0.169
$N_{as.factor}$	484		
Observations	4840		

17.4 Study 4.

Supplementary Table 60. Overview of the main model including age (centered) in Study 4.

	**Dependent variable**		
Predictors	*Odds Ratios*	*CI*	*p*
1|2	0.04	0.03 – 0.05	**<0.001**

2|3	0.13	0.11 – 0.15	**<0.001**
3|4	0.29	0.25 – 0.34	**<0.001**
4|5	0.69	0.59 – 0.81	**<0.001**
5|6	2.07	1.77 – 2.43	**<0.001**
Treatment [Partner]	1.12	0.89 – 1.41	0.344
Treatment [Oath]	0.72	0.58 – 0.89	**0.003**
Treatment [Partner+Oath]	0.77	0.61 – 0.97	**0.026**
Age	0.98	0.97 – 0.99	**<0.001**
Treatment [Partner] * Age	1.00	0.99 – 1.02	0.687
Treatment [Oath] * Age	1.00	0.99 – 1.02	0.743
Treatment [Partner+Oath] * Age	1.01	0.99 – 1.03	0.336
$N_{as.factor}$	1541		
Observations	3896		

17.5 Study 5.

Supplementary Table 61. Overview of the main model including age (centered) in Study 5.

Dependent variable

Predictors	*Odds Ratios*	*CI*	*p*
1|2	0.07	0.06 – 0.09	**<0.001**
2|3	0.20	0.17 – 0.24	**<0.001**
3|4	0.46	0.39 – 0.53	**<0.001**
4|5	1.07	0.92 – 1.25	0.389
5|6	2.88	2.45 – 3.39	**<0.001**
Treatment [Partner]	1.31	1.05 – 1.63	**0.016**
Treatment [Oath]	0.76	0.61 – 0.94	**0.011**
Treatment [Partner+Oath]	1.10	0.89 – 1.37	0.364
Age	1.00	0.98 – 1.03	0.800
Treatment [Partner] * Age	0.99	0.96 – 1.03	0.618
Treatment [Oath] * Age	0.97	0.94 – 1.00	0.069
Treatment [Partner+Oath] * Age	0.98	0.95 – 1.01	0.260
$N_{as.factor}$	982		
Observations	2491		

17.6 Study 6.

Supplementary Table 62. Overview of the main model including age (centered) in Study 6.

	**Rating**		
Predictors	*Odds Ratios*	*CI*	*p*
1|2	0.05	0.03 – 0.07	**<0.001**
2|3	0.12	0.09 – 0.16	**<0.001**
3|4	0.26	0.20 – 0.33	**<0.001**
4|5	0.62	0.49 – 0.79	**<0.001**
5|6	1.70	1.33 – 2.16	**<0.001**
Treatment [Partner]	0.97	0.68 – 1.38	0.851
Treatment [Oath]	1.16	0.82 – 1.64	0.409
Treatment [Partner+Oath]	0.97	0.68 – 1.38	0.857
Age	0.97	0.95 – 0.99	**<0.001**
Treatment [Partner] * Age	1.01	0.98 – 1.04	0.372
Treatment [Oath] * Age	1.01	0.98 – 1.03	0.673
Treatment [Partner+Oath] * Age	1.02	0.99 – 1.05	0.189

Observations	755
R^2 Nagelkerke	0.034

17.6 Study 7.

Supplementary Table 63. Overview of the main model including age (centered) in Study 7. Outcome variable is reported ratings in the die roll game (for partner and partner+oath treatments only for Player A).

	**Rating**		
Predictors	*Odds Ratios*	*CI*	*p*
1|2	0.13	0.11 – 0.14	**<0.001**
2|3	0.32	0.29 – 0.35	**<0.001**
3|4	0.66	0.60 – 0.73	**<0.001**
4|5	1.35	1.23 – 1.48	**<0.001**
5|6	3.47	3.15 – 3.82	**<0.001**
Treatment [Partner]	1.18	0.99 – 1.42	0.066
Treatment [Oath]	0.83	0.73 – 0.95	**0.006**
Treatment [Partner+Oath]	1.00	0.84 – 1.20	0.966
Age	0.99	0.98 – 1.00	**0.010**
Treatment [Partner] * Age	1.00	0.98 – 1.01	0.643
Treatment [Oath] * Age	1.00	0.99 – 1.01	0.730
Treatment [Partner+Oath] * Age	1.01	1.00 – 1.02	0.156
**Random Effects**			
σ^2	3.29		
$\tau_{00\ ID\_i}$	0.76		
ICC	0.19		
N_{ID_i}	1193		
Observations	20360		
Marginal R^2 / Conditional R^2	0.006 / 0.193		

Supplementary Table 64. Overview of the main model including age (centered) in Study 7. Outcome variable is reported doubles in the die roll game.

	Double		
Predictors	*Odds Ratios*	*CI*	*p*
(Intercept)	0.37	0.32 – 0.44	**<0.001**
Treatment [Partner]	1.41	1.05 – 1.91	**0.024**
Treatment [Oath]	0.75	0.60 – 0.94	**0.014**
Treatment [Partner+Oath]	1.26	0.94 – 1.69	0.117
Age	0.99	0.97 – 1.00	**0.027**
Treatment [Partner] * Age	0.99	0.97 – 1.01	0.469
Treatment [Oath] * Age	1.00	0.98 – 1.01	0.638
Treatment [Partner+Oath] * Age	1.01	0.99 – 1.03	0.419
**Random Effects**			
σ^2	3.29		
τ_{00} ID	2.08		
ICC	0.39		
N ID	1190		
Observations	11900		
Marginal R^2 / Conditional R^2	0.018 / 0.398		

18. Commitment & Dishonesty Rating

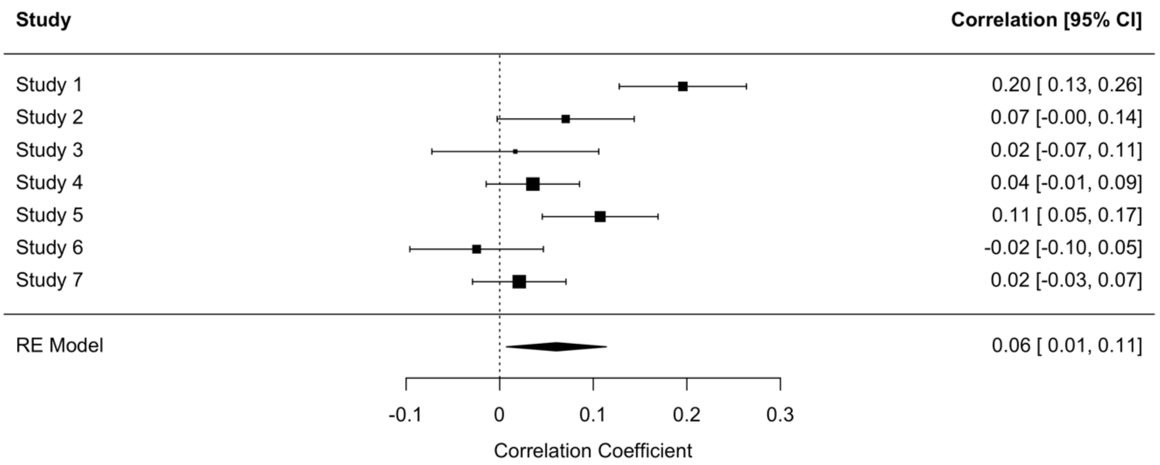

Supplementary Figure 16. Overview of correlations between commitment (pre) and task score across the seven studies.

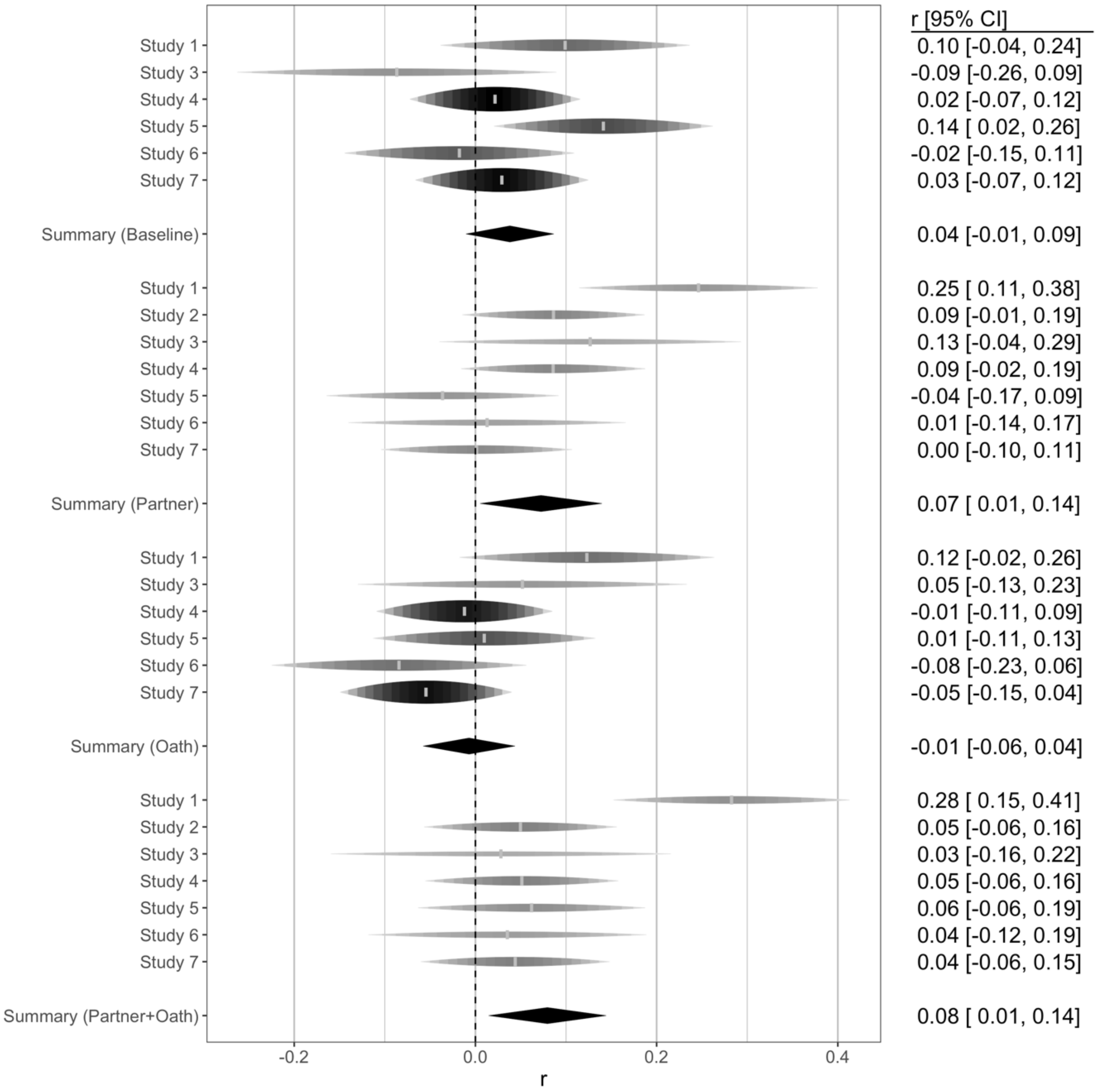

Supplementary Figure 17. Overview of correlations between commitment (pre) and task score per treatment across the seven studies.

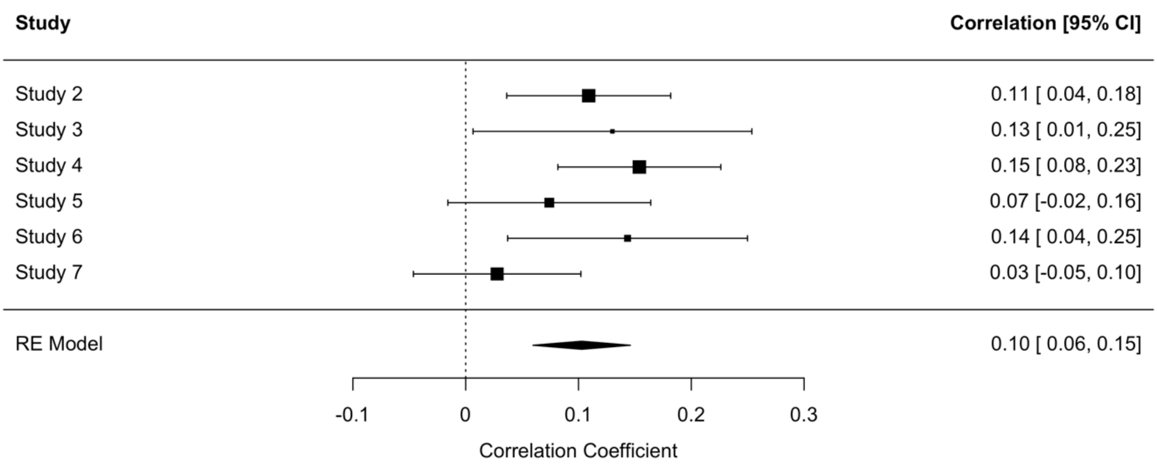

Supplementary Figure 18. Overview of correlations between commitment (post) and task score across the seven studies.

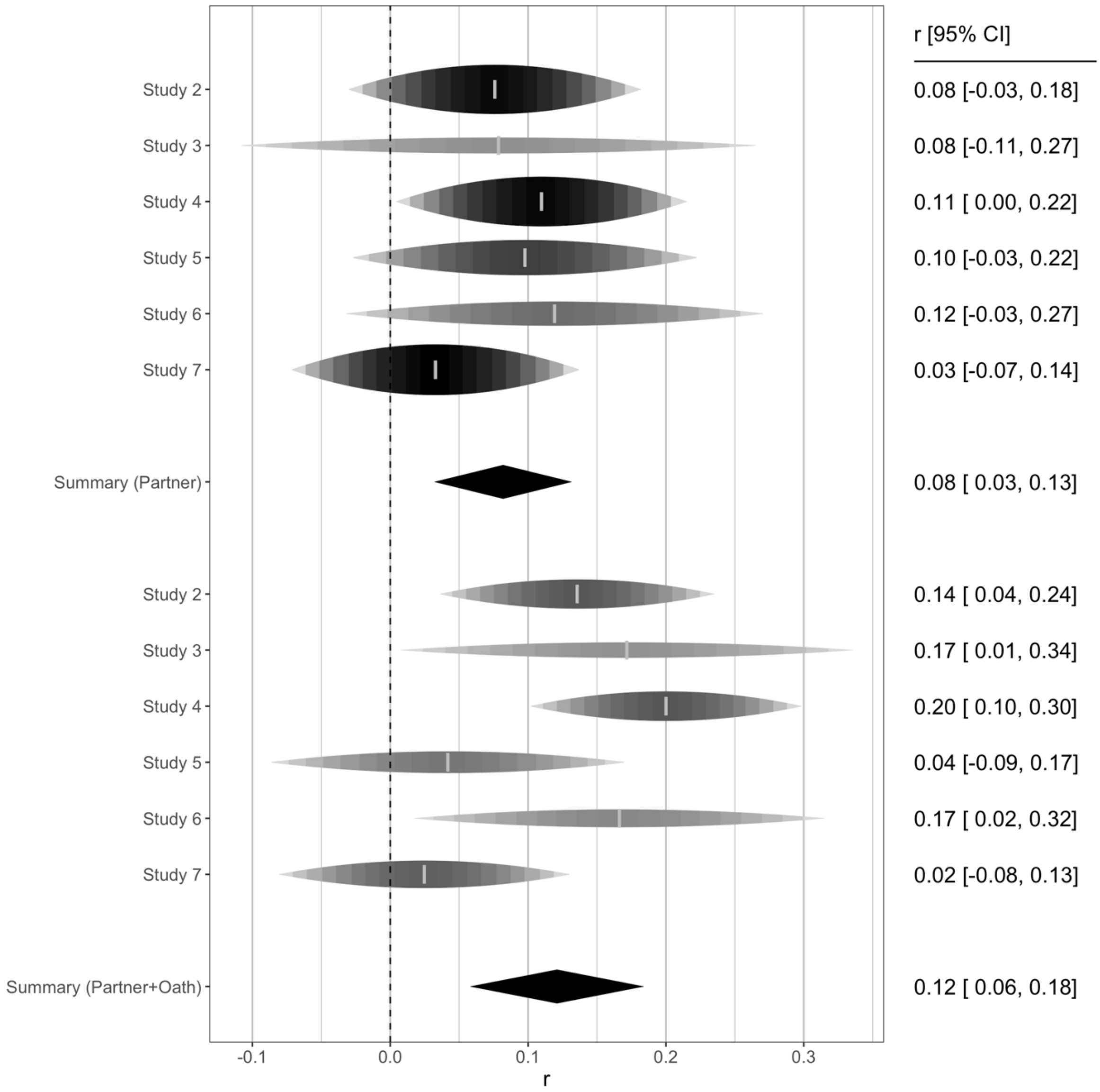

Supplementary Figure 19. Overview of correlations between commitment (post) and task score per treatment across the seven studies.

19. Comparison Across Treatments

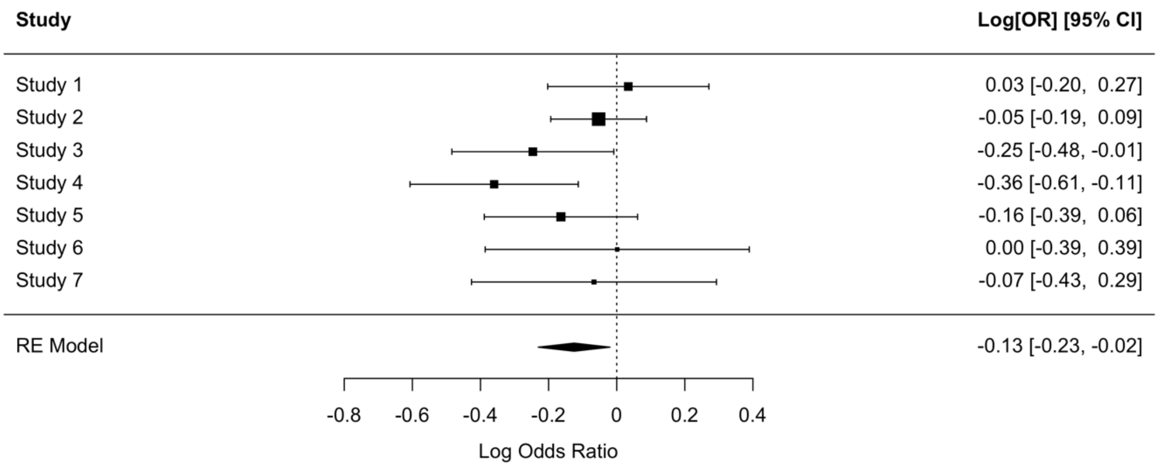

Supplementary Figure 20. Overview of comparing game scores between partner+oath and partner treatments. The effect suggests more honesty in the partner+oath treatment.

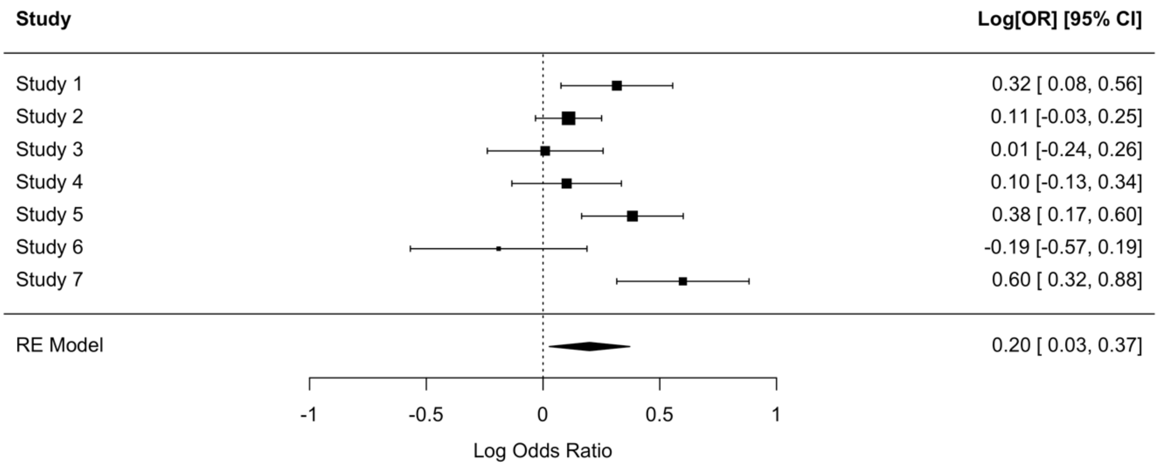

Supplementary Figure 21. Overview of comparing game scores between partner+oath and oath treatments. The effect suggests more dishonesty in the partner+oath treatment.

20. Country Differences

Supplementary Table 65. Overview of country-level indicators across the three sampled populations. Values are adapted from [40]. For all details see: https://osf.io/ub9tp/

Domain	Indicator	Mexico	UK	USA	Source
	Cultural Tightness Looseness (CTL)	1.69	1.77	1.82	[41]
Demographic	Historical Heterogeneity of Migration	0.63	0.94	0.03	[42]
	Population Density (pop/km^2)	64.35	277	34	(List of Countries and Dependencies by population density, n.d.)[a]
	Urban population	80.2	83.4	82.3	(United Nations Development Programme (UNDP), 2017)
Health	Life Expectancy at Birth	70.21	80.74	77.20	(UNDP, 2021)[b]
Work	Employment in Agriculture	13	1	1.4	
Education	Education (Expected years of schooling)	14.86	17.31	16.28	
Gender	Gender inequality Index (GII)	0.31	0.09	0.18	
Development	Human Development Index (HDI)	0.76	0.93	0.92	
Well-Being	Subjective Well Being (SWB)	6.60	7.23	6.89	[43]
	Social Support	0.86	0.93	0.90	
	Pathogen Prevalence	37	26	29	[44]
Climate	Climate Demandingness	49	67	79	[45d]
Economic	Gini Index (GINI)	45.4	35.1	34.8	[46]
	GDP per capita (PPP)	10045.7	46510.3	70248.6	[47]
Religiosity	Religiosity	63.3	29.9	65	[48]
Hofstede Dimensions	Individualism (IND)	30	89	91	[49,50]
	Masculinity (MAL)	69	66	62	
	Uncertainty Avoidance (UA)	82	35	46	
	Long Term Orientation (LTO)	24	51	26	
	Indulgence (ID)	97	69	68	
Personality	Extraversion	2.44	2.48	2.48	[51 c]
	Agreeableness	3.15	3.30	3.42	
	Conscientiousness	2.80	2.85	3.00	
	Neuroticism	2.98	2.96	2.83	
	Openness	3.55	3.60	3.59	
GLOBE	Humane Orientation	3.98	3.72	4.17	[52]
	In-Group Collectivism	5.71	4.08	4.25	
	Power Distance (PD)	2.85	2.8	2.85	
	Trust	10.5	45.4	37	[53]
Morality	Civic Honesty (returned wallets)	21	56.6	52.8	[54]

	Morality as Cooperation	6.72	6.09	6.66	[55]
	Narcissism	3.64	3.1	4.71	
	Moral Identity	7.23	6.53	6.7	
	Honesty Humility	0.38	0.44	0.38	[56]

Note. [a]https://en.wikipedia.org/wiki/List_of_countries_and_dependencies_by_population_density
[b]https://hdr.undp.org/data-center/human-development-index#/indicies/HDI
[c]we eventually employed data based on: https://www.kaggle.com/tunguz/big-five-personality-test
[d] https://www.amazon.com/Climate-Affluence-Culture-Psychology/dp/0521517877?asin=0521517877&revisionId=&format=4&depth=1

21. Additional Analyses on Dyads in Study 7

In Study 7, participants in the *partner* and *partner+oath* treatments could be matched with another participant from the SAME or a DIFFERENT treatment. In total, 82 dyads consisted of participants from both the *partner* treatments, 85 dyads consisted of participants from both the *partner+oath* treatment, and 190 mixed dyads.

Supplementary Table 66 Overview of reported doubles per group or individual focusing on dyad composition in Study 7.

	Double		
Predictors	*Odds Ratios*	*CI*	*p*
(Intercept)	0.38	0.32 – 0.45	**<0.001**
Partner Dyad	1.20	0.80 – 1.78	0.380
Oath	0.72	0.57 – 0.91	**0.006**
Partner+Oath Dyad	1.24	0.83 – 1.84	0.290
Mixed Dyad	1.35	1.01 – 1.80	0.043
**Random Effects**			
σ^2	3.29		
τ_{00} ID	2.11		
ICC	0.39		
N ID	1199		
Observations	11990		
Marginal R^2 / Conditional R^2	0.010 / 0.397		

Supplementary Table 67. Overview of overall ratings per group or individuals focusing on dyad compositions in Study 7.

	Rating		
Predictors	*Odds Ratios*	*CI*	*p*

1|1 5	0.02	0.02 – 0.03	**<0.001**
1 5|2	0.05	0.05 – 0.06	**<0.001**
2|2 5	0.13	0.11 – 0.14	**<0.001**
2 5|3	0.22	0.20 – 0.25	**<0.001**
3|3 5	0.43	0.39 – 0.49	**<0.001**
3 5|4	0.81	0.72 – 0.90	**<0.001**
4|4 5	1.54	1.37 – 1.73	**<0.001**
4 5|5	2.61	2.33 – 2.93	**<0.001**
5|5 5	5.75	5.10 – 6.47	**<0.001**
5 5|6	9.92	8.75 – 11.23	**<0.001**
Partner Dyad	1.06	0.80 – 1.40	0.683
Oath	0.78	0.67 – 0.91	**0.002**
Partner+Oath Dyad	0.95	0.73 – 1.25	0.722
Mixed Dyad	0.87	0.71 – 1.06	0.165
**Random Effects**			
σ^2	3.29		
$\tau_{00\ ID}$	1.02		
ICC	0.24		
N ID	1199		
Observations	11990		
Marginal R^2 / Conditional R^2	0.003 / 0.239		

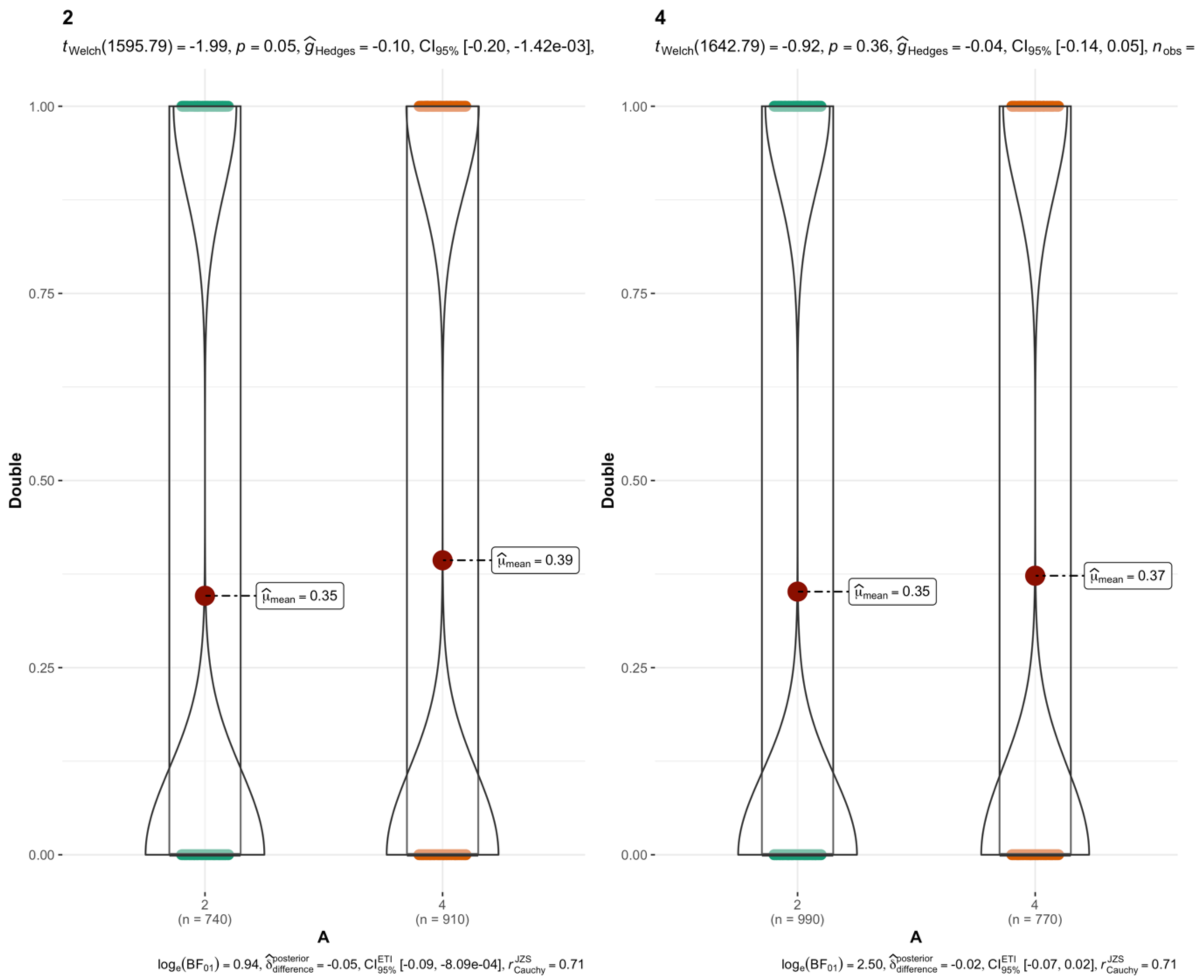

Supplementary Figure 22. Overview of double reports across the ten rounds for Player A (x-axis) and Player B (facet) in the *partner* (2) and *partner+oath* (4) treatments in Study 7.

22. Dishonesty Analyses

Supplementary Table 68. Overview of reports by treatment compared to levels of honest reporting using a Wilcoxon rank-sum test across the seven studies.

Study	Target/Task	Baseline	Partner	Oath	Partner+Oath
		r, p, n			
Study 1	Other	r = .22, p_{adj} < .001, n = 792	r = .27, p_{adj} < .001, n = 776	r = .12, p_{adj} = .003, n = 752	r = .27, p_{adj} < .001, n = 760
Study 1	Self	r = .38, p_{adj} < .001, n = 792	r = .43, p_{adj} < .001, n = 776	r = .33, p_{adj} < .001, n = 752	r = .39, p_{adj} < .001, n = 760
Study 2	Mind Shape	r = .14, p_{adj} < .001, n = 892	r = .15, p_{adj} < .001, n = 788	r = .04, p_{adj} > .999, n = 776	r = .10, p_{adj} = .034, n = 696
Study 2	Die Roll	r = .22, p_{adj} < .001, n = 768	r = .18, p_{adj} < .001, n = 704	r = .12, p_{adj} = .008, n = 696	r = .17, p_{adj} < .001, n = 656

Study 3	Mind Shape	$r = .43$, $p_{adj} < .001$, $n = 580$	$r = .35$, $p_{adj} < .001$, $n = 800$	$r = .24$, $p_{adj} < .001$, $n = 520$	$r = .26$, $p_{adj} < .001$, $n = 620$
Study 3	Die Roll	$r = .26$, $p_{adj} < .001$, $n = 650$	$r = .28$, $p_{adj} < .001$, $n = 550$	$r = .15$, $p_{adj} < .001$, $n = 650$	$r = .16$, $p_{adj} = .003$, $n = 470$
Study 4	Four Trials	$r = .49$, $p_{adj} < .001$, $n = 884$	$r = .53$, $p_{adj} < .001$, $n = 636$	$r = .37$, $p_{adj} < .001$, $n = 904$	$r = .38$, $p_{adj} < .001$, $n = 716$
Study 4	One-Shot	$r = .58$, $p_{adj} < .001$, $n = 211$	$r = .56$, $p_{adj} < .001$, $n = 207$	$r = .53$, $p_{adj} < .001$, $n = 180$	$r = .54$, $p_{adj} < .001$, $n = 158$
Study 5	Four Trials	$r = .35$, $p_{adj} < .001$, $n = 524$	$r = .48$, $p_{adj} < .001$, $n = 464$	$r = .24$, $p_{adj} < .001$, $n = 524$	$r = .43$, $p_{adj} < .001$, $n = 500$
Study 5	One-Shot	$r = .51$, $p_{adj} < .001$, $n = 123$	$r = .67$, $p_{adj} < .001$, $n = 115$	$r = .39$, $p_{adj} < .001$, $n = 126$	$r = .51$, $p_{adj} < .001$, $n = 115$
Study 6	Shared Payoff	$r = .62$, $p_{adj} < .001$, $n = 237$	$r = .60$, $p_{adj} < .001$, $n = 84$	$r = .62$, $p_{adj} < .001$, $n = 191$	$r = .54$, $p_{adj} < .001$, $n = 75$
Study 6	Double		$r = .59$, $p_{adj} < .001$, $n = 80$		$r = .69$, $p_{adj} < .001$, $n = 88$
Study 7	Report (Player A)	$r = .19$, $p_{adj} < .001$, $n = 8380$	$r = .26$, $p_{adj} < .001$, $n = 3560$	$r = .10$, $p_{adj} < .001$, $n = 8480$	$r = .21$, $p_{adj} < .001$, $n = 3440$
Study 7	Double (Dyad)	$r = .07$, $p_{adj} < .001$, $n = 4190$	$r = .19$, $p_{adj} < .001$, $n = 1670$	$r = .07$, $p_{adj} > .999$, $n = 4240$	$r = .16$, $p_{adj} < .001$, $n = 1800$

Note. r = rank biserial correlation. Wilcoxon rank-sum test/Mann-Whitney U test against H_0 (mu = 3.5; .16667 for Study 7 double) testing whether H_a is greater (i.e., higher reports). P-value adjustment via Bonferroni.

Supplementary References

1. Zickfeld, J. H., Karg, S. T., Engen, S. S., Michael, J. & Mitkidis, P. Committed Dishonesty: A Systematic Meta-Analysis of the Effect of Social Commitment on Dishonest Behavior. *PsyArXiv* (2022) doi:10.31234/osf.io/j47ng.
2. Harrell Jr, F. E. & Harrell Jr, M. F. E. Package 'hmisc'. *CRAN2018* **2019**, 235–236 (2019).
3. Weisel, O. & Shalvi, S. The collaborative roots of corruption. *Proceedings of the National Academy of Sciences* **112**, 10651–10656 (2015).
4. Tajfel, H., Billig, R., Bundy, C. & Flament, C. Social categorization and intergroup behavior". *European Journal of Social Psychology* **1**, 149–178 (1971).
5. Koch, A., Speckmann, F. & Unkelbach, C. Q-SpAM: How to Efficiently Measure Similarity in Online Research. *Sociological Methods & Research* 0049124120914937 (2020) doi:10.1177/0049124120914937.
6. Schild, C., Heck, D. W., Scigala, K. A. & Zettler, I. Revisiting REVISE: (Re)Testing unique and combined effects of REminding, VIsibility, and SElf-engagement manipulations on cheating behavior. *Journal of Economic Psychology* **75**, 102161 (2020).
7. Shu, L. L., Mazar, N., Gino, F., Ariely, D. & Bazerman, M. H. Signing at the beginning makes ethics salient and decreases dishonest selfreports in comparison to signing at the end. *Proceedings of the National Academy of Sciences* **109**, 15197–15200 (2012).
8. Jacquemet, N., Luchini, S., Rosaz, J. & Shogren, J. F. Truth telling under oath. *Management Science* **65**, 426–438 (2018).
9. Hong, Y. & Ratner, K. G. Minimal but not meaningless: Seemingly arbitrary category labels can imply more than group membership. *Journal of Personality and Social Psychology* **120**, 576 (2021).

10. Chen, Y. & Li, S. X. Group identity and social preferences. *American Economic Review* **99**, 431–57 (2009).

11. Jiang, T. *Other-regarding preferences and other-regarding cheating—Experimental evidence from China, Italy, Japan and the Netherlands*. (SSRN Working Paper, 2014).

12. Rong, R., Houser, D. & Dai, A. Y. Money or friends: Social identity and deception in networks. *European Economic Review* **90**, 56–66 (2016).

13. Jiang, T. Cheating in mind games: The subtlety of rules matters. *Journal of Economic Behavior & Organization* **93**, 328–336 (2013).

14. Gerlach, P., Teodorescu, K. & Hertwig, R. The truth about lies: A meta-analysis on dishonest behavior. *Psychological Bulletin* **145**, 1–44 (2019).

15. Fischbacher, U. & Föllmi-Heusi, F. Lies in disguise—an experimental study on cheating. *Journal of the European Economic Association* **11**, 525–547 (2013).

16. Chen, C. *et al.* Creativity in drawings of geometric shapes: A cross-cultural examination with the consensual assessment technique. *Journal of Cross-Cultural Psychology* **33**, 171–187 (2002).

17. Dehaene, S., Izard, V., Pica, P. & Spelke, E. Core knowledge of geometry in an Amazonian indigene group. *Science* **311**, 381–384 (2006).

18. Spelke, E. S. & Kinzler, K. D. Core knowledge. *Developmental Science* **10**, 89–96 (2007).

19. Aron, A., Aron, E. N. & Smollan, D. Inclusion of Other in the Self Scale and the structure of interpersonal closeness. *Journal of Personality and Social Psychology* **63**, 596–612 (1992).

20. Ashton, M. C. & Lee, K. The HEXACO-60: A short measure of the major dimensions of personality. *Journal of Personality Assessment* **91**, 340–345 (2009).

21. Tangney, J. P., Baumeister, R. F. & Boone, A. L. High Self-Control Predicts Good Adjustment, Less Pathology, Better Grades, and Interpersonal Success. *Journal of Personality* **72**, 271–322 (2004).
22. Conrads, J., Irlenbusch, B., Rilke, R. M. & Walkowitz, G. Lying and team incentives. *Journal of Economic Psychology* **34**, 1–7 (2013).
23. Wiltermuth, S. S. Cheating More when the Spoils Are Split. *Organizational Behavior and Human Decision Processes* **11**, 157–68 (2011).
24. Lakens, D. Performing high-powered studies efficiently with sequential analyses. *European Journal of Social Psychology* **44**, 701–710 (2014).
25. Pahl, R. & Pahl, M. R. Package 'GroupSeq'. (2018).
26. Molnar, A. SMARTRIQS: A Simple Method Allowing Real-Time Respondent Interaction in Qualtrics Surveys. *Journal of Behavioral and Experimental Finance* **22**, 161–169 (2019).
27. Kocher, M. G., Schudy, S. & Spantig, L. I lie? We lie! Why? Experimental evidence on a dishonesty shift in groups. *Management Science* **64**, 3995–4008 (2018).
28. Griskevicius, V. *et al.* When the economy falters, do people spend or save? Responses to resource scarcity depend on childhood environments. *Psychological science* **24**, 197–205 (2013).
29. Soraperra, I. *et al.* The bad consequences of teamwork. *Economics Letters* **160**, 12–15 (2017).
30. De Vries, R. E. The 24-item brief HEXACO inventory (BHI). *Journal of Research in Personality* **47**, 871–880 (2013).
31. Pahl, R., Ziegler, A. & König, I. R. GroupSeq: Designing clinical trials using group sequential designs. *The Newsletter of the R Project Volume 6/2, May 2006* **6**, 21 (2006).

32. Green, P. & MacLeod, C. J. SIMR: an R package for power analysis of generalized linear mixed models by simulation. *Methods Ecol Evol* **7**, 493–498 (2016).

33. Bates, D., Mächler, M., Bolker, B. & Walker, S. Fitting linear mixed-effects models using lme4. *Journal of Statistical Software* (2014).

34. Champely, S. *et al.* pwr: Basic functions for power analysis. (2017).

35. Brant, R. Assessing proportionality in the proportional odds model for ordinal logistic regression. *Biometrics* 1171–1178 (1990).

36. Abeler, J., Nosenzo, D. & Raymond, C. Preferences for Truth-Telling. *Econometrica* **87**, 1115–1153 (2019).

37. Leib, M., Köbis, N., Soraperra, I., Weisel, O. & Shalvi, S. Collaborative dishonesty: A meta-analytic review. *Psychological Bulletin* **147**, 1241 (2021).

38. Bellé, N. & Cantarelli, P. What causes unethical behavior? A meta-analysis to set an agenda for public administration research. *Public Administration Review* **77**, 327–339 (2017).

39. Lovakov, A. & Agadullina, E. R. Empirically Derived Guidelines for Effect Size Interpretation in Social Psychology. *European Journal of Social Psychology* **n/a**, (2021).

40. Zickfeld, J. H. *et al.* Tears evoke the intention to offer social support: A systematic investigation of the interpersonal effects of emotional crying across 41 countries. *Journal of Experimental Social Psychology* **95**, 104137 (2021).

41. Eriksson, K. *et al.* Perceptions of the appropriate response to norm violation in 57 societies. *Nat Commun* **12**, 1481 (2021).

42. Putterman, L. & Weil, D. N. Post-1500 population flows and the long-run determinants of economic growth and inequality. *The Quarterly journal of economics* **125**, 1627–1682 (2010).

43. Sachs, J. D., Layard, R. & Helliwell, J. F. *World Happiness Report 2019*. https://worldhappiness.report/ed/2019/ (2019).

44. Fincher, C. L. & Thornhill, R. Parasite-stress promotes in-group assortative sociality: The cases of strong family ties and heightened religiosity. *Behavioral and Brain Sciences* **35**, 61–79 (2012).

45. Van de Vliert, E. Climate, affluence and culture Cambridge University Press. *Cambridge UK* (2009).

46. World Bank, World Development Indicators. GINI Index. (2019).

47. World Bank, World Development Indicators. GDP per capita, PPP. (2019).

48. Joshanloo, M. Cultural religiosity as the moderator of the relationship between affective experience and life satisfaction: A study in 147 countries. *Emotion* **19**, 629 (2019).

49. Hofstede, G. Dimensionalizing cultures: The Hofstede model in context. *Online readings in psychology and culture* **2**, 8 (2011).

50. Taras, V., Steel, P. & Kirkman, B. L. Improving national cultural indices using a longitudinal meta-analysis of Hofstede's dimensions. *Journal of World Business* **47**, 329–341 (2012).

51. Allik, J. *et al.* Mean profiles of the NEO personality inventory. *Journal of Cross-Cultural Psychology* **48**, 402–420 (2017).

52. House, R. J., Hanges, P. J., Javidan, M., Dorfman, P. W. & Gupta, V. *Culture, leadership, and organizations: The GLOBE study of 62 societies*. (Sage publications, 2004).

53. Inglehart, R. *et al.* World Values Survey: Round Six-Country-Pooled Datafile 2010-2014. *JD Systems Institute, Madrid* (2014).

54. Cohn, A., Maréchal, M. A., Tannenbaum, D. & Zünd, C. L. Civic honesty around the globe. *Science* **365**, 70–73 (2019).

55. Azevedo, F. *et al.* Social and moral psychology of COVID-19 across 69 countries. *Scientific Data* **10**, 272 (2023).

56. Lee, K. & Ashton, M. C. Sex differences in HEXACO personality characteristics across countries and ethnicities. *Journal of Personality* **88**, 1075–1090 (2020).
