# Supplementary material for: Commitment to honesty oaths decreases dishonesty, but commitment to another individual does not affect dishonesty
Source: Commun Psychol. 2023 Oct 24;1:27. doi: 10.1038/s44271-023-00028-7 (PMC11332245; doi:10.1038/s44271-023-00028-7)
Supplement: Supplementary file 3 — Reporting Summary [file 44271_2023_28_MOESM3_ESM.pdf]

nature portfolio

Corresponding author(s): Janis Zickfeld

Last updated by author(s): Sep 5, 2023

Reporting Summary

Nature Portfolio wishes to improve the reproducibility of the work that we publish. This form provides structure for consistency and transparency in reporting. For further information on Nature Portfolio policies, see our Editorial Policies and the Editorial Policy Checklist.

Statistics

For all statistical analyses, confirm that the following items are present in the figure legend, table legend, main text, or Methods section.

n/a	Confirmed	
☐	☒	The exact sample size (*n*) for each experimental group/condition, given as a discrete number and unit of measurement
☐	☒	A statement on whether measurements were taken from distinct samples or whether the same sample was measured repeatedly
☐	☒	The statistical test(s) used AND whether they are one- or two-sided<br>*Only common tests should be described solely by name; describe more complex techniques in the Methods section.*
☐	☒	A description of all covariates tested
☐	☒	A description of any assumptions or corrections, such as tests of normality and adjustment for multiple comparisons
☐	☒	A full description of the statistical parameters including central tendency (e.g. means) or other basic estimates (e.g. regression coefficient) AND variation (e.g. standard deviation) or associated estimates of uncertainty (e.g. confidence intervals)
☐	☒	For null hypothesis testing, the test statistic (e.g. *F*, *t*, *r*) with confidence intervals, effect sizes, degrees of freedom and *P* value noted<br>*Give P values as exact values whenever suitable.*
☒	☐	For Bayesian analysis, information on the choice of priors and Markov chain Monte Carlo settings
☐	☒	For hierarchical and complex designs, identification of the appropriate level for tests and full reporting of outcomes
☐	☒	Estimates of effect sizes (e.g. Cohen's *d*, Pearson's *r*), indicating how they were calculated

Our web collection on statistics for biologists contains articles on many of the points above.

Software and code

Policy information about availability of computer code

Data collection	Qualtrics, SMARTRIQS (Molnar, 2019)
Data analysis	R (version 4.0.3; R Core Team, 2020) and the following packages: metafor (version 3.0-2; Viechtbauer, 2010), ordinal (version 2019.12-10; Christensen, 2015), MASS (version 7.3-53; Ripley et al., 2013), tidyverse (version 1.3.0; Wickham et al., 2019), ggpubr (version 0.4.0; Kassambara, 2017), TOSTER (version 0.3.4; Lakens, 2017), simr (version 1.0.6; Green & MacLeod, 2016), lme4 (version 1.1-32; Bates et al., 2015).

For manuscripts utilizing custom algorithms or software that are central to the research but not yet described in published literature, software must be made available to editors and reviewers. We strongly encourage code deposition in a community repository (e.g. GitHub). See the Nature Portfolio guidelines for submitting code & software for further information.

Data

Policy information about availability of data

All manuscripts must include a data availability statement. This statement should provide the following information, where applicable:

- Accession codes, unique identifiers, or web links for publicly available datasets
- A description of any restrictions on data availability
- For clinical datasets or third party data, please ensure that the statement adheres to our policy

The datasets generated by the survey research during and/or analyzed during the current study are available in the osf.io repository, https://osf.io/sxbfn/

Research involving human participants, their data, or biological material

Policy information about studies with human participants or human data. See also policy information about sex, gender (identity/presentation), and sexual orientation and race, ethnicity and racism.

Reporting on sex and gender	We collected self-reported gender across all studies. The supplementary materials report one analysis on gender.
Reporting on race, ethnicity, or other socially relevant groupings	We did not collect information on participant's race,ethnicity or other socially relevant groupings across the 7 studies.
Population characteristics	See below
Recruitment	Participants in study 1 and 2-7 were recruited via Prolific.co. All participants signed up at the platform that specified their place of residency as UK (Study 1, 2-4), Mexico (Study 5), and the US (Study 6-7) could participate in the current studies. There is a degree of self-selection as people sign up at Prolific.co to complete research studies. Study 2 recruited participants via a panel provider Toluna.com. The sample was originally intended to be representative of the population by gender, age, and region. However, due to a technical error some combinations were oversampled, which resulted in sampling additional participants to fill up the remaining combinations. There is also a possible self-selection bias as people actively need to be part of the panel.
Ethics oversight	Internal Review board Aarhus University (2021-103; 2022-013; 2022-073).

Note that full information on the approval of the study protocol must also be provided in the manuscript.

Field-specific reporting

Please select the one below that is the best fit for your research. If you are not sure, read the appropriate sections before making your selection.

☐ Life sciences ☒ Behavioural & social sciences ☐ Ecological, evolutionary & environmental sciences

For a reference copy of the document with all sections, see nature.com/documents/nr-reporting-summary-flat.pdf

Behavioural & social sciences study design

All studies must disclose on these points even when the disclosure is negative.

Study description	Quantitative experimental
Research sample	We recruited participants via the crowdworking website Prolific.co (Palan & Schitter, 2018) in Study 1 and Studies 3-7 and via the panel provider Toluna in Study 2. Studies 1-4 sampled participants in the UK, while Study 5 sampled participants in Mexico and Study 6-7 participants in the United States. All participants received a base study compensation according to the length of the study and were able to receive an additional bonus payment on top based on their performance in the game task (participants received a base payment of £2 in Study 1; 1200pts in Study 2, and £1 in Study 3-7; they could earn a bonus of up to £4 in Study 1, 1200pts in Study 2, £2 in Study 3-6, and £1.80 in Study 7). Studies 1 and 3-7 focused on convenience samples, while the sample in Study 2 can be considered representative according to age, gender, and region. After applying all exclusion criteria, we recruited a total of 7,566 participants across seven studies (3859 females, 3608 males, 76 non-binary, 23 not specified) ranging from 18 to 93 years (M = 41.3, SD = 15.4).
Sampling strategy	Convenience sampling.<br>We performed a-priori power analyses based on different effect sizes found across the seven studies. A detailed overview is provided in Table 1 and Supplementary Material Sections 1 & 2. For Study 1, we based the sample size on the smallest effect size (the effect of social commitment to individuals on honesty, g = -.22) as reported in the meta-analysis by Zickfeld et al. (2022). In Study 2, we focused on the meta-analytic effect from this meta-analysis for the specific paradigm used (for investment/effort tasks, g = -.19).7 Study 3 used the oath effect size obtained in Study 1 and 2. In Study 4 and 5 we used the effect size for the oath treatment from Study 3. In Study 6, we employed the effect size for the oath treatment from Study 4. Finally, for Study 7, we used our smallest effect size of interest (d = +/- .15). Power calculations were performed focusing on an ordinal response using the posamsize function of the hmisc package (version 4.4-1; Harrell Jr & Harrell Jr, 2019). Relative frequencies of responses were based on previous studies as detailed in Table 1. Based on these results, suggested sample sizes ranged between 1060 and 7408 participants when considering four treatments in total. Due to resource constraints and the fact that except for Study 6 all studies employed repeated measurements and therefore an increased number of observations, we registered a sample size of 800 in Study 1, 1000 in Study 2, and 500 in Study 3. For Studies 4-7, we registered a sequential analysis approach in order to adopt a more efficient way to conduct a high-powered study given the small effects of the previous studies and to save resources (Lakens, 2014). Based on the expected effect size, we set a maximum sample size at 1600 and registered four sequential analyses steps (i.e., analyzing the data at 400, 800, 1200, and 1600 participants) for Study 4-6, and set a maximum sample size of 2000 and four sequential steps (800, 1200, 1600, 2000 participants) for Study 7. As preregistered, recruitment was stopped once the main effects were statistically significant. In order to control for the Type-I-error rate (Lakens, 2014), we adjusted the alpha level at each step based on calculations using the GroupSeq package (version 1.4.0; Pahl et al., 2006; see Table 1 for more detailed information). In Study 4, we collected the full number of 1600 participants. Only in two cases, Study 5 and 6, we stopped data collections preliminarily, deviating from our registered plans in the following ways: In Study 5, we stopped during the third wave because we were not able to collect enough participants at the same

time due to a restricted participant pool, which was essential for the interactive nature of the task leading to a high number of drop-outs. In Study 6, we stopped after the second wave (i.e., 800 participants) although the main effects were not statistically significant. However, we realized that the effects were either tiny or in the opposite direction compared to the previous studies. We think that these changes were sensible and do not alter the overall patterns and findings in any substantial ways. In Study 7, we stopped data collection after 1600 participants according to our preregistered analysis plan, as our main effects were statistically significant.
As final sample sizes across all studies were smaller than the suggested sample sizes by our power analyses, we conducted post-hoc sensitivity analyses for all studies in order to investigate what effect size we could minimally detect at a power of 90% and 95% (see Supplement Section 2.1). Across studies, the minimum effect size we could detect at 90% power ranged between d = -.09 and -.19 and between d = -.10 and -.22 for 95% power. The minimum effect size was larger in Study 6 (90% d = -.30; 95% d = -.34) due to the fact that we employed a one-shot game. These effect sizes are typically considered as small in the literature (Lovakov & Agadullina, 2021) and are in the range of our smallest effect size of interest (d = +/- .15). In addition, the meta-analytic investigation across 7,576 participants helps us to increase our power to detect even smaller effect sizes, such that we can be certain that we have enough power to detect our smallest effect size of interest when combining all studies.

Data collection	Data were collected online using Qualtrics.
Timing	Study 1: 16/11/2021-23/11/2021; Study 2: 19/01/2022-07/02/2022; Study 3: 03/02/2022; Study 4: 24/03/2022- 28/03/2022; Study 5: 14/04/2022 - 21/04/2022; Study 6: 13/04/2022 - 14/04/2022; Study 7: 27/04/2023-29/04/2023
Data exclusions	Across studies, our exclusion criteria were: not consenting to starting the study (n = 119), not understanding the main task based on a probe item (n = 396), failing an attention check item (n = 42), being younger than 18 (n = 7), responding significantly faster or slower compared to the median response time as registered (n = 683), not correctly committing to the oath (by writing their name or pasting their participant ID, n = 67), being duplicates of prior participants based on the ID assigned by the crowdsourcing service (n = 52), or not being matched in the interaction task or the matched partner failing to respond (n = 21). These were preregistered. In Study 1 we also excluded participants with the wrong art preference (n = 15) or indicating the wrong group (n = 17).
Non-participation	As outlined above, n = 119 participants did not consent to take part in the study (across all 7 studies).
Randomization	Participants were randomly allocated to the different treatments in each study.

Reporting for specific materials, systems and methods

We require information from authors about some types of materials, experimental systems and methods used in many studies. Here, indicate whether each material, system or method listed is relevant to your study. If you are not sure if a list item applies to your research, read the appropriate section before selecting a response.

Materials & experimental systems

n/a	Involved in the study	
☒	☐	Antibodies
☒	☐	Eukaryotic cell lines
☒	☐	Palaeontology and archaeology
☒	☐	Animals and other organisms
☒	☐	Clinical data
☒	☐	Dual use research of concern
☒	☐	Plants

Methods

n/a	Involved in the study	
☒	☐	ChIP-seq
☒	☐	Flow cytometry
☒	☐	MRI-based neuroimaging
